# Genetic variation and structural diversity in major seed proteins among and within *Camelina* species

**DOI:** 10.1007/s00425-022-03998-w

**Published:** 2022-10-06

**Authors:** Dwayne Hegedus, Cathy Coutu, Branimir Gjetvaj, Abdelali Hannoufa, Myrtle Harrington, Sara Martin, Isobel A. P. Parkin, Suneru Perera, Janitha Wanasundara

**Affiliations:** 1 Agriculture and Agri-Food Canada, 107 Science Place, Saskatoon, SK S7N 0X2 Canada; 2 Department of Food and Bioproduct Sciences, University of Saskatchewan, Saskatoon, SK Canada; 3 Agriculture and Agri-Food Canada, London, ON Canada

**Keywords:** *Camelina sativa*, Cruciferin, Gene expression, Protein functionality, Protein modelling

## Abstract

**Main conclusion:**

Genetic variation in seed protein composition, seed protein gene expression and predictions of seed protein physiochemical properties were documented in *C. sativa* and other *Camelina* species.

**Abstract:**

Seed protein diversity was examined in six *Camelina* species (*C. hispida, C. laxa*, *C. microcarpa*, *C. neglecta*, *C. rumelica* and *C. sativa*). Differences were observed in seed protein electrophoretic profiles, total seed protein content and amino acid composition between the species. Genes encoding major seed proteins (cruciferins, napins, oleosins and vicilins) were catalogued for *C. sativa* and RNA-Seq analysis established the expression patterns of these and other genes in developing seed from anthesis through to maturation. Examination of 187 *C. sativa* accessions revealed limited variation in seed protein electrophoretic profiles, though sufficient to group the majority into classes based on high MW protein profiles corresponding to the cruciferin region. *C. sativa* possessed four distinct types of cruciferins, named CsCRA, CsCRB, CsCRC and CsCRD, which corresponded to orthologues in *Arabidopsis thaliana* with members of each type encoded by homeologous genes on the three *C. sativa* sub-genomes. Total protein content and amino acid composition varied only slightly; however, RNA-Seq analysis revealed that *CsCRA* and *CsCRB* genes contributed > 95% of the cruciferin transcripts in most lines, whereas *CsCRC* genes were the most highly expressed cruciferin genes in others, including the type cultivar DH55. This was confirmed by proteomics analyses. Cruciferin is the most abundant seed protein and contributes the most to functionality. Modelling of the *C. sativa* cruciferins indicated that each type possesses different physiochemical attributes that were predicted to impart unique functional properties. As such, opportunities exist to create *C. sativa* cultivars with seed protein profiles tailored to specific technical applications.

**Supplementary Information:**

The online version contains supplementary material available at 10.1007/s00425-022-03998-w.

## Introduction

Interest in *Camelina sativa* (camelina), grown in Europe in medieval times for food and fuel, stems from the need to diversify annual crop rotation portfolios with those that have smaller environmental footprints and the potential to produce valuable secondary products. It is compatible with practices used to produce contemporary oilseed crops, such as canola/oilseed rape and soybean, can be grown on marginal lands with fewer inputs and has higher tolerance to drought and cold (Vollman et al. 1996). It is also naturally resistant to several diseases (Sharma et al. 2002; Eynck et al. 2012) and insects (Deng et al. 2002; Henderson et al. 2004; Soroka et al. 2015) that afflict canola.

*Camelina sativa* seed comprises approximately 36–47% oil (Moser 2012) and 43% protein (Zubr 2003). While it is being aggressively marketed as a diesel and aviation fuel feedstock (Li and Mupondwa 2014), the high levels of polyunsaturated fatty acids, in particular α-linolenic acid (38% total fatty acid), make it an attractive source of ω3 fatty acids in food and feed. α-linolenic acid is the precursor for the essential long chain polyunsaturated fatty acids eicosapentanoic acid (20:5 ω3) and docosahexanoic acid (22:6 ω3) that have human health benefits. Farmed fish species, such as salmon and cod, can convert α-linolenic acid to these longer chain polyunsaturated fatty acids when camelina oil is substituted for fish oil in their diet (Hixson et al. 2014; Hixson and Parrish 2014). This was attributed to the induction of two genes encoding fatty acyl elongases in the livers of fish fed diets containing only camelina oil (Xue et al. 2014, 2015). Other studies have reported increased ω3 fatty acid levels in chicken meat (Ariza et al. 2010) and eggs (Kakani et al. 2012), as well as in milk (Szumacher-Strabel et al. 2011) when camelina meal is incorporated into the diet at fairly low levels. Complete replacement of fish oil with camelina oil in farmed fish diets seems possible as this has no impact on weight gain, fillet sensory quality (Hixson et al. 2014) or the ability to mount an immune response (Booman et al. 2014), though some differences in tissue lipid composition (Hixson et al. 2014) and intestinal function (Morias et al. 2012) have been noted.

Camelina meal is also being considered as a protein source in farmed fish, poultry and livestock. Atlantic cod (*Gadus morhua*) tolerated up to 24% inclusion of camelina meal in place of fish meal in their diets without affecting weight gain (Hixson et al. 2016a). Salmonids were more sensitive to fish meal replacement and tolerated up to 5% (Atlantic salmon, *Salmo salar*) (Hixson et al. 2016b) and 14% (rainbow trout, *Oncorhynchus mykiss*) (Ye et al. 2016) camelina meal in their diets without ill effects. In cod, high inclusion rates were associated with increased expression of appetite-stimulating hormones and decreased expression of appetite-suppressing hormones indicating that the meal is affecting nutritional quality or palatability (Tuziak et al. 2014). In broiler chickens, low energy and nitrogen utilisation from camelina-based meals was attributed to high jejunal digesta viscosity, likely due to high levels of seed coat mucilage remaining in the meal, and to the presence of glucosinolates which can affect palatability (Pekel et al. 2015). Conversely, in growing pigs the ileal digestibility of crude protein from camelina expeller cake was only slightly less than the comparable canola product and was recommended for use in swine diets (Almeida et al. 2013). In cattle, the amount of undegraded protein in the rumen differed among meals from ten camelina genotypes (Colombini et al. 2014), but was generally higher than for canola meal. With the exception of glucosinolates, the levels of anti-nutritional factors including phytic acid, condensed tannins and sinapine, were lower in the camelina meals than canola meal. The essential amino acid composition of camelina meal is comparable to that from canola, soybean and flax meals (Zubr 2003); however, differences in amino acid profiles among camelina lines have been reported (Colombini et al. 2014). Of particular significance are the essential amino acids lysine and methionine as they cannot be synthesised de novo by animals and must be provided in the diet, though methionine can be converted to cysteine. Both are limiting in plant-based diets, most notably in cereals and some legumes (Ufaz and Galili 2008), and are added as supplements to feeds at a significant cost to fish (Wilson and Halver 1986), poultry (Kidd et al. 1998) and swine (Brinegar et al. 1950) production.

Camelina breeding is still in its infancy, but release of the camelina genome (Kagale et al. 2014) and transcriptome data (Liang et al. 2013; Nguyen et al. 2013; Mudalkar et al. 2014; Kagale et al. 2016) will facilitate rapid advances in crop improvement. As in other Brassicaceae, the major seed proteins in camelina are of the 2S albumins (napin) and 12S globulins (cruciferin), with transcript data indicating that there are 8 and 17 expressed members of these gene families, respectively, in *C. sativa* cv. Sunesson (Nguyen et al. 2013). The napin dimer possesses four disulphide bonds and consequently these proteins are rich in cysteine, while cruciferins tend to have higher levels of lysine. Oleosins are amphiphilic proteins with well-separated hydrophilic and hydrophobic domains; an attribute that allows them to interact with both lipid and water. While less abundant than cruciferin or napin, they play a major role in seed lipid accumulation and stabilisation of oil bodies, as well as other aspects of plant development (D’Andrea 2016). Manipulation of amino acid levels is possible through mutation (Kita et al. 2010; Marsolais et al. 2010) or down-regulation (Schmidt et al. 2011) of the major seed storage protein genes.

To date, there has been no broad examination of *C. sativa* seed protein or seed amino acid content diversity. To this end, we established seed protein profiles for six *Camelina* species and 187 *C. sativa* accessions from a global diversity collection held at the Plant Gene Resources Center for Canada (pgrc.agr.gc.ca). Amino acid content was determined for representatives from each major seed protein profile group and transcriptomic analysis was conducted to catalogue the expressed seed protein genes from the most diverse lines. These studies established that there is potential to select or engineer *C. sativa* lines with altered seed protein and/or amino acid profiles that may be more useful in food/feed or technical applications.

## Materials and methods

### Plant materials

A list of Camelina species, accessions and their source is provided in Suppl. Table S1a. Another 187 *C. sativa* accessions were obtained from PGRC (Agriculture & Agri-Food Canada, Saskatoon) (Suppl. Table S1b). *C. sativa* DH55 is a doubled haploid line for which the genome sequence is available (Kagale et al. 2014).

### Seed protein extraction and separation

Seeds of *C. hispida* var. *hispida*, *C. hispida* var. *grandiflora, C. sativa*, *C. laxa*, *C. neglecta*, *C. microcarpa* (4x and 6x) and *C. rumelica* were generated at the Agriculture and Agri-Food Canada, Ottawa Research and Development Centre under controlled conditions within a growth chamber with randomised individual position and re-randomisation of position every two weeks. Self-incompatible taxa were hand pollinated to induce seed set. Seeds of *C. sativa* lines obtained from PGRC were generated at the Agriculture and Agri-Food Canada, Saskatoon Research and Development Centre. Plants were grown in 6-inch pots in a soilless medium (Stringam 1971) in a growth chamber with a photoperiod of 16 h and light/dark temperatures of 20 °C/16 °C. At maturity, water was withheld and plants allowed to dry, at which point seed was collected from the entire plant and seed from each plant kept separate.

Seeds (30 mg) from individual plants grown at the same time and under the same conditions, each representing one biological replicate, were ground under liquid nitrogen using a Helix grinder (Helix Technologies Inc., French Lick, IN, USA). The material was suspended in 1.2 ml of lysis buffer (7 M urea, 2 M thiourea, 19 mM Tris–HCl, 14 mM Tris-base, 0.2% Triton X-100) with 8% Complete mini EDTA-free protease inhibitor (Roche Diagnostics, Laval, Canada), 1.5 mg/ml DNase I (Roche Diagnostics) in dilution buffer (10 mM Tris–Cl pH 7.5, 150 mM NaCl, 1 mM MgCl_2_), and 0.01 mg/ml bovine pancreas RNase A (Sigma-Aldrich, Oakville, Canada) added just prior to use (Withana-Gamage et al. 2013a). Soluble proteins were isolated by centrifugation at 10,000 g for 20 min. Disulfide bonds were reduced by incubation for 30 min at 4 °C with 1.0 mM DTT when required. Protein concentrations were determined using a Qubit 2.0 Fluorometer (Thermo Fisher Scientific, Nepean, Canada).

An Experion Pro260 analysis kit (Bio-Rad Laboratories, Mississauga, Canada) was used to determine the relative proportion of each protein based on size from the seed extracts. Fresh, not frozen, protein samples were adjusted to 0.5 µg/µl and treated according to the manufacturer’s protocol (Experion Pro260 Analysis kit, Bio-Rad Laboratories). In brief, gel solution, gel-stain solution, Pro260 ladder and sample buffer were prepared with Experion Pro260 analysis kit reagents. Note only the Pro260 ladder was heated to 100 °C; the samples were heated to 65 °C to prevent thiourea in the buffer from denaturing the proteins. Experion Pro260 chip micro-channels were used to separate proteins on an Experion automated electrophoresis station (Bio-Rad Laboratories). The resulting electropherograms were analysed using the percentage determination function in the Experion software which calculates each protein peak as a percent of the total protein within the sample.

### Amino acid analysis

Seeds (3 g) from individual plants grown at the same time and under the same conditions, each representing one biological replicate, were defatted using hexane based on the methods of Troeng (1955) and Barthet and Daun (2004). Seeds were placed in sealed, steel tubes with 3 ball bearings and 25 ml of hexane (Sigma-Aldrich). Samples were ground for 45 min using an Eberbach shaker followed by filtration to remove oils and hexanes. Defatted meal was air-dried overnight followed by storage at − 20 °C. Total nitrogen content of the defatted meal was determined using a Flash EA 112 Series N/Protein 2000 Organic Elemental Analyzer (Thermo Fischer Scientific). This system uses a dynamic flash combustion system coupled with a gas chromatographic separation system based on the AOAC Method 972.43 (1999). Approximately, 15 mg of defatted meal from each sample (biological replicate) was analysed in triplicate (technical replicates). The nitrogen to protein conversion factor used was 6.25 (Mariotti 2008; AACC Method 46–18.01 1999). Moisture levels in the defatted meal were determined as weight loss upon drying to stability at 105 °C for 24 h in a forced air oven (AACC Method 44–01.01 1999). Approximately, 700 mg of defatted camelina meal was dried for each sample.

Amino acid profiles were analysed following the procedure of AOAC Method 994.12 (2005) and Tuan and Phillips (1997). Tryptophan was quantified following method of Nielsen and Hurrell (1985). For *C. sativa* lines from the PGRC repository, protein hydrolysis was conducted using a microwave, acid hydrolysis method modified from Lill et al. (2007) and Kabaha et al. (2011). Acid hydrolysis converts asparagine and glutamine into aspartic acid and glutamic acid, respectively; therefore, these amino acids are quantified together. Separation and quantification of amino acids was performed using a high-performance liquid chromatography (HPLC) system (Waters Alliance 2695) equipped with a Waters 2475 fluorescence detector with excitation wavelength of 250 nm, emission wavelength of 395 nm. Amino acids were resolved using a multistep gradient elution with an injection volume of 5 μl. Response peaks were recorded with the software Empower (Waters Corporation, Brossard, Canada). Pre-column derivatization using AccQ-Fluor (Waters Corporation) was done for all samples, except tryptophan which was diluted prior to application. For all amino acids except cysteine, methionine and tryptophan, 5 mg of protein basis was hydrolysed with 6 M HCL (Optima grade, Thermo Fisher Scientific) with 1% phenol using a CEM Discover SPD Microwave digester (ramp time 5.5 min, hold at 195 °C for 10 min, maximum pressure at 140 psi and maximum power at 300 W). Hydrolysates were neutralised with sodium hydroxide, filtered through a 0.45 μm Phenex RC syringe filter and applied to a Waters Oasis HLB C18 Cartridge. Flow through and washes were collected. Cysteine and methionine were determined as cystic acid and methionine sulfone after oxidation with performic acid followed by microwave hydrolysis with 6 M HCl, then neutralised and filtered as described. Tryptophan was determined by hydrolysing 10 mg of protein in 4.2 M NaOH in a 10 ml quartz hydrolysis tube with a teflon liner using a CEM Discover SPD Microwave digester (ramp time 6.0 min, hold at 215 °C for 20 min, with maximum pressure set at 140 psi and maximum power at 300 W). Hydrolysed samples were neutralised with HCl and filtered prior to application on a Waters Oasis HLB C18 Cartridge. The flow-through and washes were collected. Samples were stored at -20 °C prior to dilution and HPLC analysis. DL 2-aminobutyric acid and DL 5-methyl-tryptophan (Sigma-Aldrich) were used as internal standards. For experiments with *Camelina* species, amino acid analysis was conducted as described above, except the hydrolysis was performed as follows. Defatted meal was placed into 10 ml Pyrex screw cap vials with protein equivalents of 5 mg (nitrogen to protein conversion factor of 6.25). Hydrolysis was done in 2 ml of 6 M HCl (Optima grade, Thermo Fisher Scientific) with 1% (w/v) phenol for 24 h at 110 °C, with the exception of cysteine and methionine which were oxidised to cystic acid and methionine sulfone prior to hydrolysis in 6 M HCl. Tryptophan was not assessed.

Amino acids were reported as % w/w (weight of the specific amino acid/weight of all amino acids recovered X-100). For samples from each biological replicate, representing single plants grown at the same time and under the same conditions, amino acid and nitrogen analysis were performed in triplicate (technical replicates) and moisture determination as a single reading. Technical replications of the same sample presenting a large coefficient of variation (> 10) were repeated. Statistical differences between biological replicates were identified using JMP 13 software. A one-way analysis of variance (ANOVA) and the multiple comparison Tukey honestly significant difference (HSD) test were used to identify and rank significant differences (*P* ≤ 0.05).

### RNA-Seq analysis

*C. sativa* DH55 flower buds along the main raceme were marked at anthesis and developing bolls taken every 4 days from anthesis to seed maturity (40 days). RNA was isolated separately from samples from each time point. Buds from lines identified as belonging to one of three protein profile groups, either Group 1 (CN113733 and CN30476), Group 2 (CN30477and CN45816), or Group 3 (CN111331 and CN114265), were also marked at anthesis and bolls sampled similarly; however, prior to RNA isolation, equal amounts (by weight) of material from each time point were pooled into a single sample representing an average developmental profile for each line. This allowed the suite of seed protein genes expressed in each line to be compared, although it was not possible to determine when they were expressed. RNA isolation was performed similar to Suzuki et al. (2004) with volumes modified to allow extraction in 1.5 ml tubes. RNA was quantified on a Qbit using the BR RNA kit (Invitrogen/Thermo Fisher Scientific), and library generation (Truseq stranded mRNA kit) and Illumina sequencing (800,000–1,000,000 reads per sample) were performed by the National Research Council of Canada DNA Services Lab (Saskatoon, Canada). Reads were trimmed for adapters and quality using Trimmomatic 0.30, with a phred 33 quality score cutoff of 15 used for leading, trailing, and sliding window (4 bp) trimming, discarding any reads with under 55 bp remaining after trimming. CLC Genomics Workbench 11.0.1 was used to run RNAseq Analysis (version 2.1), which mapped the reads to the genome and calculated the transcripts per million (TPM). Quantile normalisation was applied to improve between-sample comparisons.

### Proteomics analysis

Seed protein was solubilised in non-reducing protein loading buffer (2% SDS, 10% glycerol, 0.01% bromophenol blue in 60 mM Tris–HCl buffer, pH 6.8) and separated by electrophoresis on 12% SDS-PAGE gels. A high molecular weight region (49–54 kDa) was cut from the gel and subjected to LS-MS/MS analysis at the Genome BC Proteomics Centre, University of Victoria, Canada, as per the following procedure. Trypsin digests were performed as previously described (Loiselle et al. 2005). Briefly, the gel slice was cut into 1 mm cubes and transferred to a Genomics Solutions Progest (DigiLab Inc., Holliston, MA, USA) perforated digestion tray. The gel pieces were de-stained (methanol/water/acetic acid, 50/45/5, by vol.) prior to reduction with 10 mM dithiothreitol and alkylation with 100 mM iodoacetamide. Modified sequencing-grade porcine trypsin solution (20 ng/µl) (Promega, Madison, WI, USA) was added at an enzyme/protein ratio of 1:50. Proteins were then digested for 5 h at 37 °C prior to collection of the tryptic digests and acid extraction of the gel slices (acetonitrile/water/formic acid, 50/40/10, by vol.). The samples were then lyophilised and stored at − 80 °C prior to analysis.

The peptide digest was separated by on-line reverse-phase chromatography using an EASY-nLC II system (Thermo Fisher Scientific) with a reverse-phase Magic C-18AQ pre-column (100 µm I.D., 2 cm length, 5 µm, 100 Å) and reverse-phase nano-analytical column Magic C-18AQ (75 µm I.D., 15 cm length, 5 µm, 100 Å) (Michrom BioResources Inc., Auburn, AL, USA) both prepared in-house, at a flow rate of 300 nl/min. The chromatography system was coupled on-line with an LTQ Orbitrap Velos mass spectrometer equipped with a Nanospray II source (Thermo Fisher Scientific). Solvents were A: 2% acetonitrile, 0.1% formic acid; B: 90% acetonitrile, 0.1% formic acid. After pre-column (~ 10 µl, 249 bar) and nanocolumn (~ 6 µl, 249 bar) equilibration, samples were separated by gradient elution (0 min: 5% B; 45 min: 45% B; 2 min: 80% B; hold 8 min: 80% B). The LTQ Orbitrap Fusion (Thermo Fisher Scientific) parameters were as follows: nano-electrospray ion source with spray voltage 2.1 kV, capillary temperature 225 °C. Survey MS1 scan m/z range 400–2,000 profile mode, resolution 60,000 FWHM at 400 m/z with AGC target 1E6, and one microscan with maximum inject time of 500 ms. Lock mass Siloxane 445.120024 for internal calibration with preview mode for FTMS master scans: on, injection waveforms: on, monoisotopic precursor selection: on; rejection of charge state: 1. The samples were analysed by the following methods: (1) top 15 FTMS/IT-CID method with the fifteen most intense ions charge state 2–4 exceeding 5000 counts were selected for CID ion trap MS/MS fragmentation (ITMS scans 2–16) with detection in centroid mode. Dynamic exclusion settings were: repeat count: 2; repeat duration: 15 s; exclusion list size: 500; exclusion duration: 60 s with a 10 ppm mass window. The CID activation isolation window was: 2 Da; AGC target: 1E4; maximum inject time: 100 ms; activation time: 10 ms; activation Q: 0.250; and normalised collision energy 35%.

A database was generated based on the published proteome of *C. sativa* (Kagale et al. 2014, 2016) and common contaminant sequences (human keratin and porcine trypsin) added. All cruciferin, napin, vicilin, and oleosin sequences were manually curated prior to inclusion in the database. The following sequences were corrected: napins (Csa11g017000, Csa12g024720, Csa12g024730), cruciferins (Csa14g004960, Csa03g005050, Csa11g015240), vicilins (Csa19g031870, Csa01g025880, Csa01g025890, Csa16g016660, Csa05g038120) and oleosin (Csa12g079570). All seed protein sequences were deposited in Genbank (accessions OL404969-OL405008). Tandem mass spectra were extracted, charge state deconvoluted and deisotoped by Proteome Discoverer version 1.4. All MS/MS samples were analysed using Mascot version 1.4.1.14 (Matrix Science, London, UK). Mascot was set up to search with a fragment ion mass tolerance of 0.60 Da and a parent ion tolerance of 8.0 PPM. Carbamidomethyl of cysteine was specified as a fixed modification. Deamidation of asparagine and glutamine, oxidation of methionine and propionamide of cysteine were specified as variable modifications. Scaffold (version Scaffold_4.8.4, Proteome Software Inc., Portland, OR, USA) was used to validate MS/MS based peptide and protein identifications. Peptide identifications were accepted if they could be established at greater than 95.0% probability by the Scaffold Local FDR algorithm. Protein identifications were accepted if they could be established at greater than 95.0% probability and contained at least 2 identified peptides. Protein probabilities were assigned by the Protein Prophet algorithm (Nesvizhskii et al. 2003). Proteins that contained similar peptides and could not be differentiated based on MS/MS analysis alone were grouped to satisfy the principles of parsimony. Proteins sharing significant peptide evidence were grouped into clusters.

### Phylogenetic analysis

Phylogenetic analysis was conducted using MEGA version 6.06 (Tamura et al. 2013). Sequences were aligned using MUSCLE with parameters set at gap opening penalty 10, gap extension penalty 0.2 and gap separation distance 4 for protein alignments and gap opening penalty 15, gap extension penalty 6.66, transition weight 0.5 for DNA alignments. Maximum likelihood trees were constructed using the best substitution model for each data set with 500 bootstrap iterations.

### Protein modelling

The Swiss Model First Approach (Waterhouse et al. 2018) was used to identify the best template and to generate an initial structure for each cruciferin. The SWISS-MODEL template library (SMTL version 2020-05-20, PDB release 2020-05-15) (Bienert et al. 2017) was searched for evolutionary-related structures matching the target sequence using default settings (http://swissmodel.expasy.org). The best template, PDB 3KGL.1.A, was found with HHblits and identified as a homo-trimer. The template structure was obtained from X-ray crystallography with a resolution of 2.98 angstroms. A structural alignment was calculated and the fit adjusted to the template using Swiss PDB Viewer, SPDBV (https://spdbv.vital-it.ch). The resultant structurally aligned SPDBV project files were submitted to Swiss Model workspace. Loops were constructed for untemplated regions and adjacent residues with low root mean square differences (RMSD) using the Scan Loop Data Base for realistic loop options. When an acceptable loop was not identified, the residues associated with the loop were submitted for modelling to the DaReUS-Loop server (https://bioserv.rpbs.univ-paris-diderot.fr/services/DaReUS-Loop). Energy minimization of the structure was done after loop selection. Energy minimization computations (bonds, angles, torsion, improper, non-bonded and electrostatic) were conducted with the GROMOS96 module in Swiss PDB Viewer. Model quality was reviewed using QMEAN and GMQE from Swiss Model, Ramachandran plot statistics were calculated using ProCheck (https://servicesn.mbi.ucla.edu/PROCHECK) and Z-Score from ProSA (https://prosa.services.came.sbg.ac.at/prosa.php). RMSD of the final structure was calculated for the structurally-aligned residues against the template 3KGL.1.A using Swiss PDB Viewer (van Gunstern 1996).

Electrostatic surface potentials of the molecules were calculated using the default settings in the APBS electrostatic plugin (Dolinsky et al. 2007). The molecule was prepared using PDB2PQR workflow to add missing side chains and hydrogen atoms, to assign partial charges and radii, and to remove ligands. The electrostatic map was calculated with the grid spacing set to 0.5 with molecular surface visualisation set at ± 5 on the solvent-excluded surface (Connolly surface). The protein dielectric constant was set at 2, the solvent dielectric constant at 78, and the temperature at 310 K. Hydrophobicity was ranked using the Eisenberg scale (Eisenberg et al. 1984). Models were coloured using the color_h pyMol script (https://pymolwiki.org/index.php/Color_h).

ClustalW was used for multiple sequence alignments. Evolutionary sequence conservation was determined using the ConSurf server (https://consurf.tau.ac.il/) (Landau et al. 2005). Phosphorylation sites were identified using Net Phos 2.0 (http://www.cbs.dtu.dk/services/NetPhos-2.0). PyMol (https://pymolwiki.org/index.php/FindSurfaceResidues) was used to colour each of the identified sites. Surface accessible phosphorylation sites on the trimer were identified using the find surface residues feature in PyMol. The cutoff to define exposed or not exposed residues was set at 2.0 squared Angstroms. CAST-P (computed atlas of surface topography of proteins) was used to calculate the main pocket of the trimer. Pocket volume, area, circumference, openings and sum of mouth areas were reported using Connolly solvent-excluded surface area, which is the contact surface created when a sphere of size 1.4 angstroms is rolled over the model.

## Results

### Seed protein profile diversity in Camelina species

**Fig. 1:**
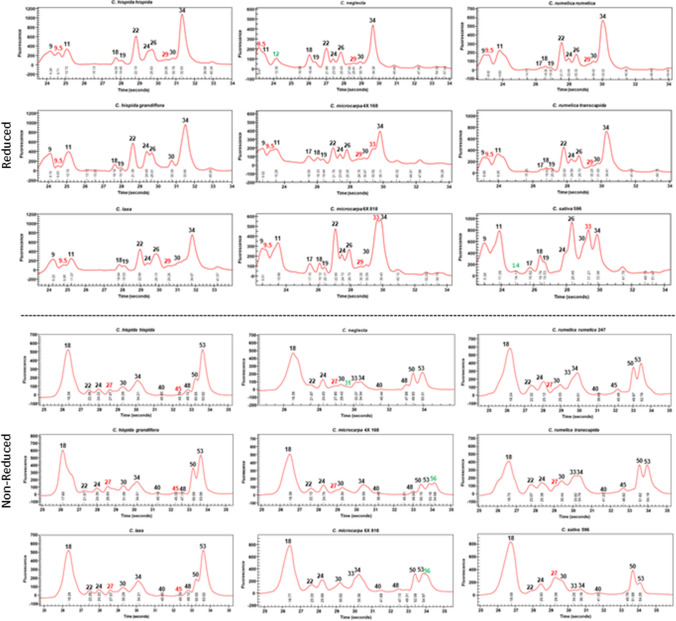
Seed protein profiles from various *Camelina* species. Traces were generated by capillary electrophoretic separation of total seed protein under reducing (upper panel) and non-reducing (lower panel) conditions. Commons peaks (black numbers), peaks differing between species (red numbers) and peaks unique to a species (green numbers)

Total seed protein from lines representing the spectrum of *Camelina* species (Suppl. Table S1) was separated by capillary electrophoresis under reducing (with β-ME) and non-reducing conditions (without β-ME) (Fig. 1; Suppl. Table S2). While many of the major peaks were in common between the species, a scheme to differentiate them based on unique peaks and patterns specific to each was developed (Suppl. Fig. S1). The *C. sativa*/*C. microcarpa* 4X/*C. microcarpa* 6X/*C. rumelica rumelica*/*C. rumelica transcapida* group could be differentiated from the *C. neglecta*, *C. laxa*/*C. hispida hispida*/*C. hispida grandiflora* group by the presence or absence of a 17 kDa peak under reducing conditions. *C. sativa* could then be differentiated by the presence of a 14 kDa peak and *C. rumelica rumelica*/*C. rumelica transcapida* differentiated from *C. microcarpa* 4X/*C. microcarpa* 6X by the presence or absence of a 33 kDa peak. *C. microcarpa* 4X exhibited a 54 kDa peak under non-reducing conditions, while *C. microcarpa* 6X did not. *C. neglecta* could be differentiated from *C. laxa*/*C. hispida hispida*/*C. hispida grandiflora* by a 12 kDa peak under reducing conditions and the latter further differentiated by 33 and 29 kDa peaks.

### Protein and amino acid content in meal from Camelina species

The percent protein of defatted meal varied considerably between species, but generally less so between accessions of the same species (Table 1). Meal from the *C. microcarpa* 4X lines exhibited the lowest protein content, approximately 31%, while meal from *C. hispida hispida*, *C. laxa*, *C. rumelica transcapida* and lines within the *C. rumelica rumelica* and *C. sativa* groups approached or exceeded 40%. Amino acid content in the meal also varied significantly within and between species (Table 2). Of the essential amino acids most often added as supplements to feeds, lysine levels varied from a low of 4.77% (w/w) in meal from *C. rumelica rumelica* 609 to a high of 5.74% in *C. sativa* 1063 meal. Meal from the *C. sativa* lines generally had higher levels of lysine. Of the sulphur-containing amino acids, methionine was highest in meal from *C. rumelica rumelica* 609 and lowest in *C. microcarpa* 6X 198 meal, while cysteine was highest in *C. rumelica rumelica* 247 meal, but lowest in *C. rumelica rumelica* 1034 meal. Interestingly, histidine levels were significantly higher in the meal from *C. rumelica rumelica* 1034 (4.77%), which was almost twice that found in meal from the other species. Serine content was highest (5.39%) in *C. sativa* 605 meal, but lowest (4.43%) in meal from another *C. sativa* line, 252. Threonine was also lowest (3.83%) in meal from *C. sativa* line 1662, but exceeded 4.5% in other *C. sativa* lines and other *Camelina* species.

**Table 1:** Protein content in meal from various *Camelina* species

Species	Line	Protein^1^ (%)	SD	Significance category^2^
*C. hispida grandiflora*	248	36.17	3.71	>BCDEFGHI
*C. hispida hispida*	240	39.40	1.42	ABCD>>>>>
*C. laxa*	612	37.39	0.68	ABCDEFG>>
*C. neglecta*	246	34.06	2.66	>>>DEFGHI
*C. microcarpa* 4X	168	31.54	0.12	>>>>>>>HI
	718	31.42	0.98	>>>>>>>>I
	965	31.19	1.86	>>>>>>GHI
*C. microcarpa* 6X	198	32.94	0.89	>>>>>FGHI
	818	33.69	0.47	>>>>EFGHI
*C. rumelica rumelica*	247	39.24	0.84	ABCD>>>>>
	609	37.96	1.32	ABCDEF>>>
	1022	37.01	1.37	ABCDEFGH>
	1034	36.97	1.90	ABCDEFGH>
	1255	37.42	0.66	ABCDEFG>>
*C. rumelica transcapida*	245	40.03	2.99	ABC>>>>>>
*C. sativa*	239	41.70	0.49	AB>>>>>>>
	252	40.43	3.24	ABC>>>>>>
	596	35.78	1.07	>>CDEFGHI
	605	39.16	1.17	ABCDE>>>>
	621	41.68	0.87	AB>>>>>>>
	1044	40.72	1.21	ABC>>>>>>
	1062	39.91	0.86	ABC>>>>>>
	1063	41.83	2.03	A>>>>>>>>
	1662	42.49	0.20	A>>>>>>>>

^1^Mean ± SD (*n* = 3 biological replicates each with 3 technical replicates)

^2^Letters denote significant differences (*P* = 0.05). Tukey–Kramer comparison for least squares means

**Table 2:**
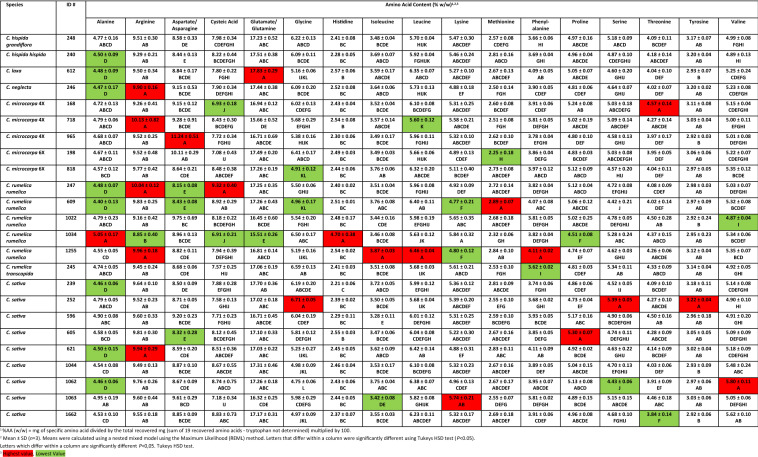
Amino acid content in meal from various *Camelina* species

### Seed protein profile diversity in C. sativa

As variation in seed protein profile was observed with the nine *C. sativa* accessions examined above, the analysis was extended to include a global collection of 187 *C. sativa* lines from the PGRC. Lines could be classified based on the similarity of seed protein profiles under reducing or non-reducing conditions. It should be noted that while classification of the lines based on protein profiles generated under the two conditions was generally in agreement, some lines were placed into different groups dependent upon the condition under which the seed protein was separated. This allowed for an even finer level of discrimination when both data sets were considered. A complete list of the lines tested with accompanying capillary electrophoresis electropherograms can be found in Suppl. Table S3.

**Fig. 2:**
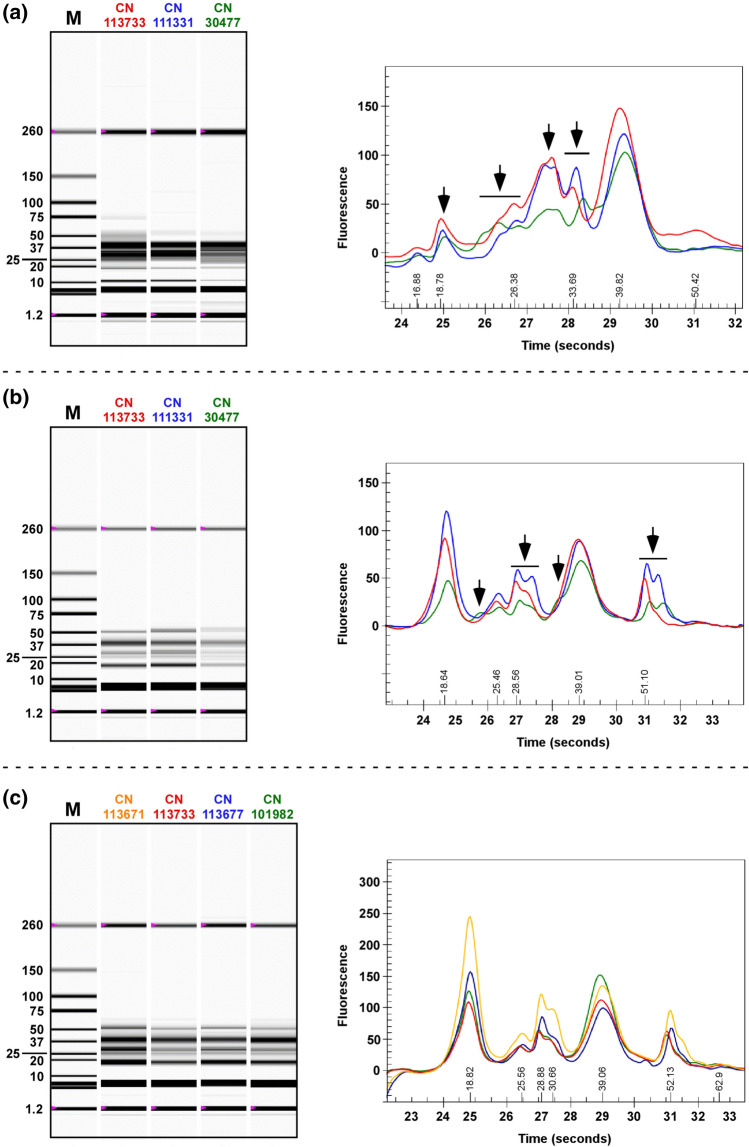
Seed protein profiles from *C. sativa* accessions. Virtual digital gels (left-hand side) and traces (right-hand side) were generated by capillary electrophoretic separation of total seed protein under reducing (**a**) and non-reducing (**b** and **c**) conditions. **a** and **b** *C. sativa* lines representing the three main profiles (profile 1—CN113733, profile 2—CN30477 and profile 3—CN111331). Arrows denote differences between profiles. **c** Variation among four lines exhibiting seed protein profile 1

Under reducing conditions, seven different profiles were noted with the majority of the lines exhibiting one of three profiles as exemplified by lines CN113733, CN111311 and CN30477 (Fig. 2). Lines with these profiles exhibited several unique protein peaks or patterns between 22 and 36 kDa (Fig. 2a). Three distinct profiles were observed under non-reducing conditions with the pattern of proteins ranging from 49 to 54 kDa being one of the more distinguishing features (Fig. 2b). Profile 1 (e.g. CN113733) had a single peak ca. 51. kDa with a small higher molecular weight (MW) shoulder. Profile 2 (e.g. CN30477) was distinguishable by a unique peak at ca. 23 kDa, by a peak at ca. 36 kDa appearing as a shoulder on a common higher MW peak at ca. 39 kDa, and by two smaller, broad peaks of relatively equal abundance at ca. 52 and 55 kDa. Lines exhibiting Profile 3 (e.g. CN111331) were similar to Profile 1, but had two large peaks at ca. 51 and 54 kDa. Lines in the same category often showed slight differences in the ratio of proteins, but the profiles were very similar (Fig. 2c).

### Protein and amino acid content in meal from diverse C. sativa accessions

Percent protein in defatted meal was found to vary considerably among the *C. sativa* lines; however, this did not correlate with protein profiles (Table 3). Meal from line CN113733 had the highest protein content (53.71%), while meal from line CN111331 had the lowest (43.26%). It should be noted that the average meal protein content among these lines (49.49%) was higher than in the nine accessions examined above (40.41%). This likely reflects the different locations and conditions under which the plants were propagated for these experiments.

**Table 3:** Protein content in meal from *C. sativa* lines with various seed protein profiles

Species	Protein Profile	Line	Protein^1^ (%)	SE	Significance category^2^
*C. sativa*	1	CN113733	53.71	0.24	A
		CN30476	47.27	0.66	C
	2	CN30477	49.55	0.74	BC
		CN45816	51.77	0.25	AB
	3	CN111331	43.26	0.39	D
		CN114265	51.44	0.97	AB

^1^Mean ± SE (*n* = 4, except for CN111331 where *n* = 3)

^2^Letters denote significant differences (*P* = 0.05). Tukey–Kramer comparison for least squares means

The amino acid content in meal from the lines representing the three seed protein profiles was also examined to estimate the extent of diversity for this trait among the lines in the PGRC collection (Table 4). While a correlation between seed protein profile and amino acid content was not observed, the lines examined exhibited significant differences in meal amino acid content. Of the essential amino acids required by monogastric animals, methionine (converted to cysteine), threonine and lysine are often lacking in plant-based diets. In this regard, meal from lines CN113733, CN30476 and CN111331 had significantly higher levels (ca. 7–8% more) of lysine, while less variation was found for methionine and threonine levels. Meal from line CN30477 had generally higher levels of essential aliphatic amino acids, namely leucine, isoleucine and valine, than the other lines, while meal from line CN114265 had significantly higher levels of cysteine. Meal from line CN45816 had significantly higher levels of glutamic acid (ca. 4–7% more), but lower levels of hydroxyl amino acids (serine, threonine and tyrosine) as did meal from line CN114265. Meal from line CN30476 had the highest levels of serine and threonine.

**Table 4:** Amino acid content in meal from *C. sativa* lines with various seed protein profiles

Amino Acid	Amino acid content (% w/w) per accession^1,2^						Average
	Seed protein profile 1		Seed protein profile 2		Seed protein profile 3		
	CN113733	CN30476	CN30477	CN45816	CN111331	CN114265	
Alanine	4.74 ± 0.12 B	4.89 ± 0.08 A	4.72 ± 0.06 BC	4.61 ± 0.11 C	5.01 ± 0.11 A	4.63 ± 0.11 BC	4.76 ± 0.16
Arginine	9.82 ± 0.29 AB	9.46 ± 0.32 C	9.59 ± 0.16 BC	9.98 ± 0.31 A	9.56 ± 0.24 BC	9.78 ± 0.31 ABC	9.69 ± 0.32
Aspartate/ Asparagine	9.45 ± 0.14 AB	9.4 ± 0.24 AB	9.59 ± 0.11 A	9.26 ± 0.45 BC	9.49 ± 0.21 AB	9.09 ± 0.22 C	9.38 ± 0.29
Cysteic Acid	3.46 ± 0.21 B	3.38 ± 0.28 B	3.13 ± 0.27 B	3.37 ± 0.62 B	3.27 ± 0.32 B	3.95 ± 0.58 A	3.44 ± 0.48
Glutamate/ Glutamine	17.68 ± 0.33 BC	17.93 ± 0.21 B	17.89 ± 0.12 B	18.63 ± 0.52 A	17.45 ± 0.47 C	17.98 ± 0.3 B	17.93 ± 0.46
Glycine	5.17 ± 0.03 C	5.41 ± 0.05 B	5.5 ± 0.05 B	5.49 ± 0.06 AB	5.64 ± 0.18 A	5.53 ± 0.18 AB	5.45 ± 0.17
Histidine	2.73 ± 0.06 A	2.69 ± 0.07 AB	2.61 ± 0.06 BC	2.67 ± 0.04 AB	2.55 ± 0.1 C	2.66 ± 0.11 AB	2.66 ± 0.09
Isoleucine	3.77 ± 0.09 B	3.71 ± 0.09 B	4.05 ± 0.09 A	3.81 ± 0.16 B	3.77 ± 0.08 B	3.72 ± 0.11 B	3.81 ± 0.16
Leucine	6.93 ± 0.14 AB	6.83 ± 0.11 B	7.04 ± 0.12 A	6.85 ± 0.12 B	6.85 ± 0.14 B	6.85 ± 0.13 B	6.9 ± 0.14
Lysine	5.81 ± 0.08 A	5.86 ± 0.09 A	5.42 ± 0.1 B	5.55 ± 0.07 B	5.8 ± 0.14 A	5.52 ± 0.16 B	5.66 ± 0.21
Methionine	1.84 ± 0.16 AB	1.77 ± 0.16 B	1.86 ± 0.18 AB	1.75 ± 0.2 B	1.85 ± 0.2 AB	2.02 ± 0.27 A	1.85 ± 0.21
Phenylalanine	4.36 ± 0.07 AB	4.37 ± 0.15 AB	4.42 ± 0.05 A	4.26 ± 0.15 B	4.36 ± 0.16 AB	4.33 ± 0.13 AB	4.36 ± 0.13
Proline	5.53 ± 0.09 A	5.39 ± 0.04 B	5.26 ± 0.06 C	5.46 ± 0.16 AB	5.55 ± 0.13 A	5.49 ± 0.11 AB	5.44 ± 0.14
Serine	4.57 ± 0.09 C	4.78 ± 0.09 A	4.59 ± 0.09 BC	4.52 ± 0.13 C	4.71 ± 0.07 AB	4.54 ± 0.09 C	4.62 ± 0.13
Threonine	3.89 ± 0.05 ABC	3.98 ± 0.11 A	3.94 ± 0.06 AB	3.81 ± 0.13 BC	3.95 ± 0.07 AB	3.81 ± 0.1 C	3.9 ± 0.11
Tryptophan	1.38 ± 0.07 A	1.23 ± 0.08 B	1.32 ± 0.08 AB	1.25 ± 0.13 B	1.25 ± 0.09 AB	1.31 ± 0.14 AB	1.29 ± 0.11
Tyrosine	3.2 ± 0.04 C	3.28 ± 0.04 AB	3.35 ± 0.02 A	3.18 ± 0.12 C	3.23 ± 0.07 BC	3.21 ± 0.06 C	3.25 ± 0.08
Valine	5.67 ± 0.11 AB	5.64 ± 0.16 AB	5.74 ± 0.1 A	5.55 ± 0.19 B	5.69 ± 0.1 AB	5.55 ± 0.18 B	5.64 ± 0.15

^1^%AA (w/w) = mg of specific amino acid divided by the total recovered mg (sum of 19 recovered amino acids–tryptophan not determined) multiplied by 100

^2^ Mean ± SD (*n* = 4 except for CN111331 where *n* = 3). Letters within a row denote significant differences (*P* = 0.05). Tukey–Kramer comparison for least squares means

### Genes encoding major seed storage proteins in C. sativa

**Fig. 3:**
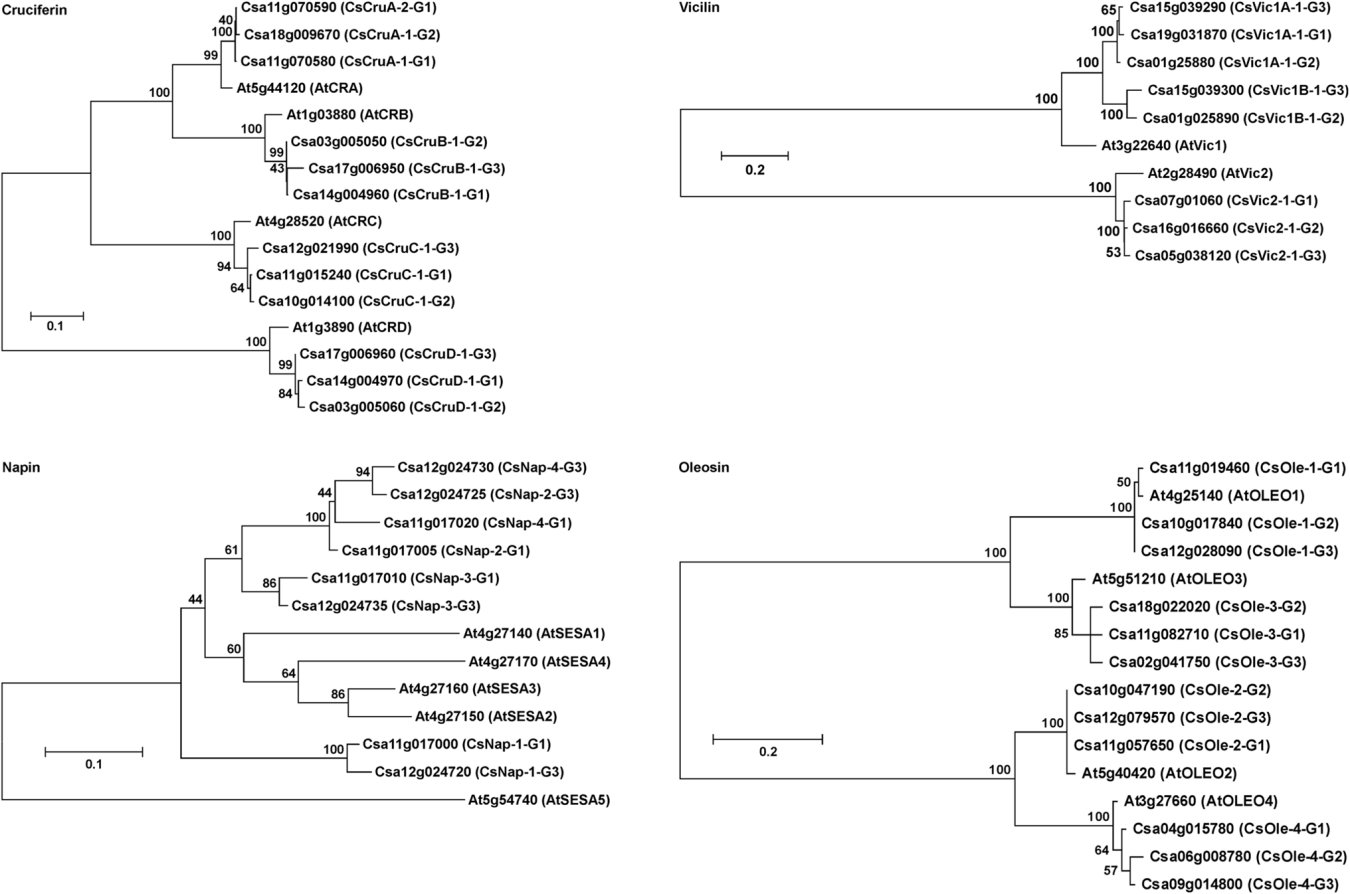
Phylogenetic analysis of major *C. sativa* seed proteins. Maximum likelihood trees were constructed using the best substitution model for each data set with 500 bootstrap iterations. Numbers beside nodes indicate percentage of trees agreeing with the consensus

Examination of the *C. sativa* DH55 genome sequence (Kagale et al. 2014) identified genes encoding major seed proteins, namely cruciferin, napin, vicilin and oleosin, which were then annotated according to their relationship to the presumed *A. thaliana* orthologues and location of the gene on a specific *C. sativa* sub-genome (Suppl. Table S4). Twelve genes encoded the main Brassicaceae seed storage protein, cruciferin, of which five were located on sub-genome I (G1), four on sub-genome II (G2) and three on sub-genome III (G3). Phylogenetic comparison to the four genes encoding cruciferin in *A. thaliana* (*AtCRA*, *AtCRB*, *AtCRC* and At1g03890) revealed that two tandemly linked genes on G1 (Csa11g070580 and Csa11g070590) and one of the genes on G2 (Csa18g009670) were most similar to *AtCRA* and were named accordingly (Fig. 3; Suppl. Fig. S2a). A *CRA* orthologue was not found on any of the *C. sativa* G3 chromosomes, while single orthologues of *AtCRB* and *AtCRC* were found on each of the three sub-genomes. The phylogenetic analysis also revealed genes encoding a fourth type of cruciferin in *C. sativa*, hereafter referred as *CsCruD*, which was most similar to the cruciferin encoded by the *A. thaliana* At1g03890 locus. Single *CsCruD* orthologues were found on each of the *C. sativa* sub-genomes, each linked in tandem to a *CsCruB* gene, which is similar to the arrangement in the *A. thaliana* genome.

Vicilin is a cupin-domain protein similar in structure to cruciferin. In total, eight genes encoding vicilin-like proteins were identified in the *C. sativa* DH55 genome (Suppl. Table S4). Phylogenetic analysis revealed that five of the *C. sativa *vicilins formed two related subgroups that were most similar to the *A. thaliana* vicilin AtPAP85 (also known as vicilin 1); accordingly, these vicilins were denoted CsVic1A and CsVic1B (Fig. 3; Suppl. Fig. S2b). The *CsVic1A* subgroup contained homeologues from all three sub-genomes (Csa19g031870, Csa1g025880 and Csa15g039290), while the *CsVic1B* subgroup included a gene on G3 (Csa15g039300) and a gene on G2 (Csa01g025890), but was missing a G1 homeologue. The two tandem *Vic1* genes on G2 represent both subgroups, as did the two tandemly linked genes on G3. The remaining vicilin genes (Csa07g016060, Csa16g016660 and Csa05g038120) were most similar to *A. thaliana* vicilin *AtVCL22* (denoted herein as vicilin 2) with homeologues present on each of the three *C. sativa* sub-genomes.

The original annotation of the *C. sativa* DH55 genome identified five genes encoding the 2S albumin, napin (Kagale et al. 2014); however, a transcriptomic study indicated that as many as eight genes might exist (Nguyen et al. 2013). As this did not correspond with the expectation of gene number based on the genomic prediction, the assembly of the genomic regions containing the napin genes was re-examined. This revealed that three of the genes that had been previously annotated as single genes by Kagale et al. (2014) were in fact closely related genes linked in tandem and had been misassembled. In agreement with the previous transcriptomic study, eight genes encoding napin were identified after separation of the tandem genes, four of which were in a cluster on G1 and four in a cluster on G3. Phylogenetic analysis revealed that the *C. sativa* napins were most similar to AtSESA1, AtSESA2, AtSESA3, and AtSESA4, which are also closely related and linked in tandem on *A. thaliana* chromosome 4, and distinct from the other *A. thaliana* napin, AtSESA5, which is encoded by a gene on chromosome 5 (Fig. 3; Suppl. Fig. S2c). Each of the eight *C. sativa* proteins could be paired to one of the eight napins reported in the earlier transcriptomic study (Nguyen et al. 2013) (Suppl. Fig. S3); however, the *C. sativa* proteins were renamed according to their genomic locations as per cruciferin and vicilin (Suppl. Table S4). No genes encoding napin were found on G2. The first two genes in the tandem series on G1 (Csa11g017000) and G3 (Csa12g024720) appear to be homeologues based on phylogenetic analysis; however, the other paralogues in each tandem series appear to have arisen through separate gene duplication events (Suppl. Fig. S2c) and the fact that there are four in each cluster appears to be coincidental.

Oleosins possess hydrophilic and hydrophobic domains that allow them to organise storage triglycerides into the oil bodies commonly found in cells of oilseed embryos. In total, 12 *C. sativa* genes were found to encode oleosins comprising three homeologues related to each of the genes encoding the four major *A. thaliana* oil body-associated oleosins (OLEO1 to 4) (Fig. 3; Suppl. Fig. S2d). *C. sativa* orthologues of members of the extended oleosin-like family were also identified.

### Temporal expression of C. sativa seed storage protein genes through seed development

RNA-Seq analysis was conducted with *C. sativa* DH55 developing bolls from anthesis to seed maturity (40 days) to ascertain the expression profile of genes encoding seed proteins (Table 5). Transcripts derived from all of the genes encoding the two major seed storage proteins, namely cruciferin (12) and napin (8), were identified. Both sets exhibited similar expression patterns with a sharp increase in expression detected between 8–12 days after anthesis (daa) and a sharp decline between 28–32 daa. Members of the tandem napin clusters on G1 and G3 differed greatly in their levels of expression, but not in their temporal patterns. The three homeologous genes encoding cruciferin CsCruD isoforms were expressed at lower levels than those encoding CsCruA, CsCruB or CsCruC suggesting that CsCruD may contribute less to overall seed protein composition. There was some evidence for genome partitioning with respect to the level of expression of homeologous genes encoding CsCruA, CsCruB or CsCruC.

The expression of the homeologous genes encoding CsVic1A on G1 (Csa19g031870), G2 (Csa01g025880) and G3 (Csa15g039290) increased sharply at 12 daa and high levels of transcripts were detected throughout seed development. The expression of the gene encoding CsVic1B on G3 (Csa15g039300) increased more gradually until 28 daa before declining sharply, while few transcripts were detected from its homeologous partner on G2 (Csa01g025890). Temporal patterns were also apparent in the expression of genes encoding oleosins. In general, the expression of genes encoding oleosins increased between 8 and 12 daa, though those encoding CsOle-1 were induced slightly earlier. Transcript levels from homeologous genes encoding CsOle-3 declined after 20 daa, while the expression of genes encoding CsOle-1, CsOle-2 and CsOle-4 remained elevated or continued to increase until the seeds were mature (40 daa). Of the other proteins known to contribute to seed protein composition, many genes encoding dehydrins or members of various late embryo abundant (LEA) protein families were also expressed at high levels during the later stages of seed development as expected (Suppl. Table S5).

### Comparison of the high molecular weight proteome in diverse C. sativa accessions

The feature that most distinguished the *C. sativa* accessions was a high molecular weight region (49–55 kDa) appearing under non-reducing conditions, therefore, proteomics analysis of this region was conducted with two lines representing each of the three major seed protein profiles observed under non-reducing conditions, namely Profile 1 (CN113733 and CN30476), Profile 2 (CN30477 and CN45816) and Profile 3 (CN111331 and CN114265) (Suppl. Fig. S4). As expected, the most abundant proteins within this fraction were cruciferins (Suppl. Table S6) of which all four types were represented. Across all lines, CsCruA (MW 52 kDa) was the most abundant cruciferin and approximately three times more so than CsCruB (MW 51 kDa). The level of CsCruD (MW 50 kDa) was low, but relatively similar among the lines, while the amount of CsCruC (MW 55 kDa) varied extensively. Higher levels of CsCruC were present in line CN45816, while lines CN113733 and CN111331 had 10–12 times less. The relative abundance of the cruciferin isoforms did not fully explain the differences in protein profiles in this region; however, other proteins of similar MW were found in this fraction, including a group of nitrile specifier proteins that were even more abundant than CsCruD.

### Comparison of the seed transcriptome in diverse C. sativa accessions

To examine the genetic basis underlying the different seed protein profiles among the *C. sativa* accessions, RNA-Seq analysis (Suppl. Table S7) was also conducted with these lines. Lines from the same seed protein profile groups did not exhibit seed protein gene expression patterns that were indicative of a specific group, although differences in temporal patterns could not be evaluated since bolls from all stages of development were pooled in this experiment. Genetic variation existed in the overall patterns between the lines and in comparison to the collective profile for *C. sativa* DH55 which has an electrophoretic protein profile similar to Profile 3 (Table 5).

The napin genes encoding CsNap-1, CsNap-3, CsNap-4 on the G1 and G3 sub-genomes were expressed at the highest levels, while genes encoding CsNap-2 were expressed at appreciably lower levels (ca. 10–50%) than the other *CsNap* genes in all of the lines. This pattern was similar to that observed with *C. sativa* DH55, although in this line *CsNap-1-G1* was expressed at a lower level and *CsNap-2-G1* at high levels. Notably, in DH55 *CsNap-2-G3* was induced much later and for a shorter period of time than the other napin genes (Table 5), which may have also contributed to the lower overall transcript levels in the other *C. sativa* lines.

The expression pattern of genes encoding CsCruA and CsCruB was similar in all *C. sativa* lines, including DH55. *CsCruA-2-G1* and *CsCruA-1-G2* were expressed at comparable levels and approximately twice that of *CsCruA-1-G1*, while the expression of the *CruB* genes was in the following order, *CsCruB-1-G3* > *CsCruB-1-G1* > *CsCruB-1-G2*. The pattern of *CruC* expression was markedly different between the lines. CN45816 and DH55 (Table 5; Suppl. Table S7) exhibited very high levels of *CsCruC-1-G3* expression (in fact, the highest of all of the cruciferin genes), high levels of *CsCruC-1-G1* expression and lower levels of *CsCruC-1-*G2 expression. Conversely, CN30476 and CN114265 expressed mainly *CsCruC-1-G3* and only at lower levels, while the CN113733, CN30477 and CN111331 possessed few or no *CruC* transcripts. As in DH55, the expression of genes encoding CsCruD was also low in the other *C. sativa* lines when compared to genes encoding CsCruA and CsCruB. Proteomic analysis of the high MW protein region, of which cruciferin was the most abundant member, confirmed these patterns (Suppl. Table S6).

The expression of vicilin genes was similar to DH55 with higher levels of expression detected from genes encoding CsVic1A and with comparatively little contribution from those encoding CsVic1B. The genes encoding CsVic2 on sub-genomes G1 and G3 were expressed at approximately 30% the level of the genes encoding CsVic1A, with the *CsVic2* gene on G2 contributing few transcripts. Genes encoding oleosins CsOle-1, CsOle-2 and CsOle-4 were expressed at higher levels than those encoding CsOle-3. This was similar to the pattern in DH55, though it should be noted that expression of genes encoding CsOle-3 declined as seed development progressed, while the expression of genes encoding the other oleosins continued to increase throughout (Table 5).

## Structural diversity of *C. sativa* cruciferins

In its natural form, cruciferin exists as a hexamer with a stochastic composition dependent on the availability of individual protomers (subunits). The functional properties of cruciferin are, therefore, an average of the functional properties of the subunits contributing to the whole. As variation was observed in the expression of genes encoding CruC and in actual cruciferin composition in the meal, the structure and potential functional properties of *C. sativa* cruciferins were examined.

**Fig. 4:**
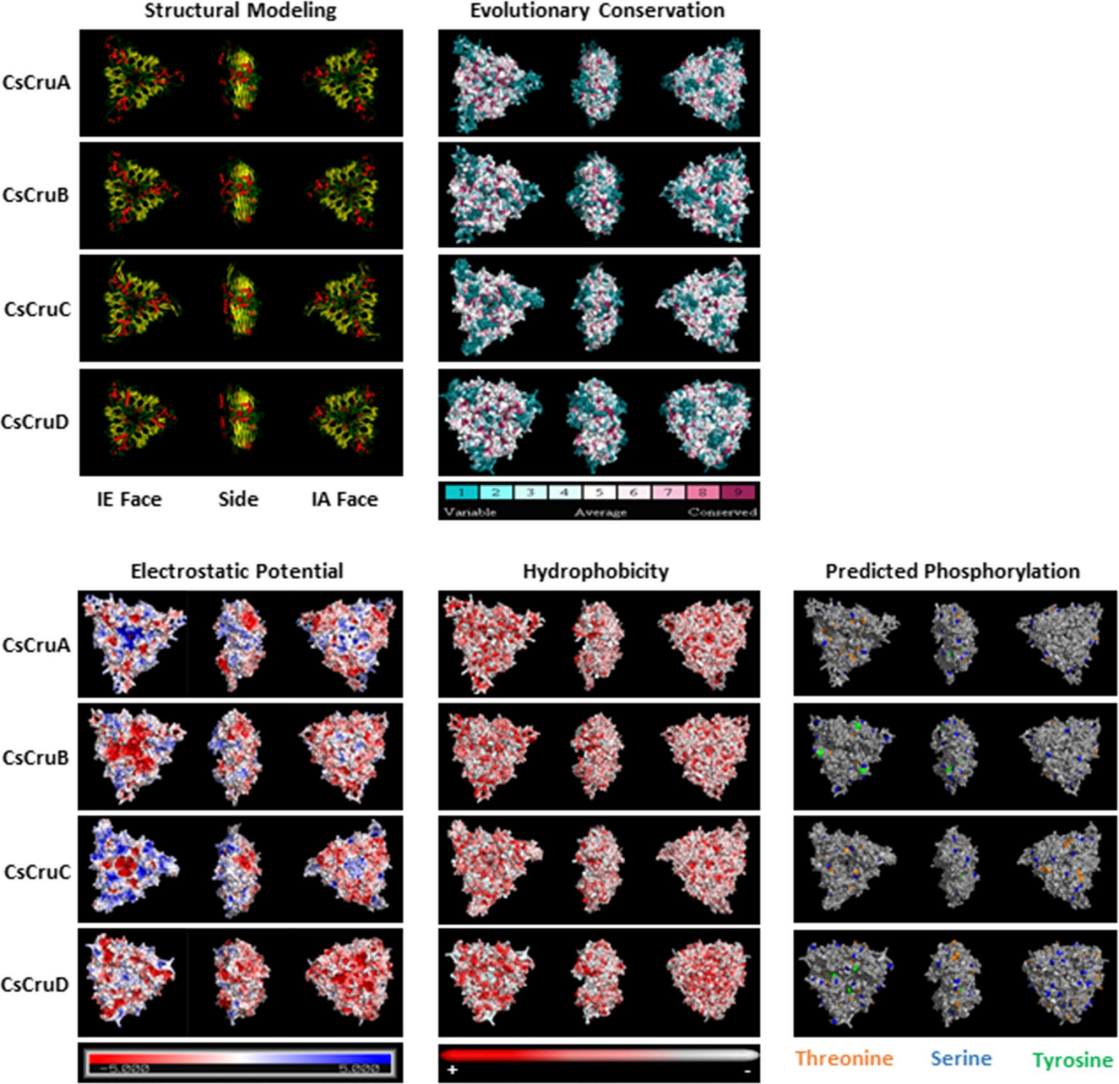
Structural modelling, evolutionary conservation, surface hydrostatic potential, surface hydrophobicity and predicted phosphorylation of *C. sativa* cruciferins. Structural modelling panel: yellow = β-sheet, red = α-helix and green = loops. IE–interchain interacting face. IA–intrachain interacting face. One representative from each cruciferin type is shown: CsCRA (CRA-1-G1), CsCRB (CRB-1-G1), CsCRC (CRC-1-G1) and CsCRD (CsCRD-1-G1)

**Fig. 5:**
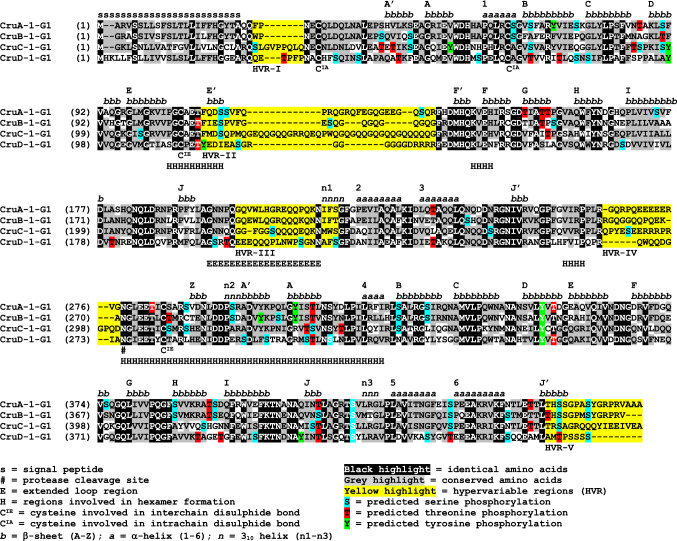
Alignment and features associated with *C. sativa* cruciferins

Homology models of *C. sativa* cruciferins representing each of the four main classes (CsCruA, CsCruB, CsCruC and CsCruD) were constructed using the *B. napus* procruciferin (Cru2/3a, PDB 3KGL) as a template (Fig. 4: Suppl. Fig. S5). The *C. sativa* cruciferins had a reasonable degree of sequence identity with the *B. napus* template: 86.9% (CsCruA), 74.3% (CsCruB), 61.6% (CsCruC) and 51% (CsCruD). The difference between CsCruC and the template was largely attributed to an extended hypervariable region (HVR) II (Fig. 5; Suppl. Fig. S6), while CsCruD is phylogenetically distinct from the other cruciferins. Nonetheless, each of the *C. sativa* cruciferins possessed a highly conserved core structure consisting of two jelly roll β-barrels and two extended helix regions comprised of 27 β-sheets, six α-helices and three 3_10_-helices, which is typical of cupin domains associated with 11S and 7S globulins (Tandang-Silvas et al. 2010). The HVR regions cannot be resolved by crystallography as they do not possess ordered secondary structures, such as β-sheets or α-helices, and likely form loops protruding from the core (Adachi et al. 2001; Tandang-Silvas et al. 2010). To account for this, the energy minimization approach used by Withana-Gamage et al. (2011) to model *A. thaliana* cruciferin loops was employed; however, models were first constructed for those loops that had a similar modelled loop in the Scan Loop Data Base. The DaReUS-Loop server was used to construct loops for those without an acceptable template in the database. Only then were stereochemical alterations made to minimise energy based on the GROMOS 96 force field calculations. Several parameters indicated that the *C. sativa* cruciferin models were of high quality and geometrically correct (Suppl. Table S8). *G*-factor scores based on torsion angles and covalent bond geometry ranged from − 0.09 to − 0.16 which was well within the generally regarding acceptable value range of 0 to − 0.5. Ramachandran plots showed that the sum of the percentage of residues in the core, allowed and additionally-allowed regions was 100% for CsCruA, CsCruB and CsCruD and 99.96% for CsCruB. Qualitative Model Energy ANalysis (QMEAN) scores, a composite measure of several geometric parameters (Benkert et al. 2008) with 0 considered as a good model and values < − 0.4 generally considered poor, ranged from − 0.98 to − 0.27. *Z*-scores, a measure of overall model quality based on the deviation of the total energy of the structure with respect to an energy distribution derived from random conformations (Benkert et al. 2011), ranged from 6.73 to 7.11. These scores were similar to models of *A. thaliana* cruciferins (Withana-Gamage et al. 2011) and within the range observed for models of proteins of similar size. RMSD derived by superimposing the *C. sativa* cruciferin models on the template indicated close alignment of the backbone with RMSD values all below 0.5 Å.

Alignment of the *C. sativa* cruciferins indicated a high degree of variability between CsCruA, CsCruB, CsCruC and CsCruD in each of the five HVRs (Fig. 5; Suppl. Fig. S6); these are also referred to as disordered regions due to their inability to be modelled or resolved by crystallographic methods (Adachi et al. 2001, 2003). HVR-I and HVR-V reside at the amino- and carboxy-terminus of the mature cruciferin, respectively, with the differences between the *C. sativa* cruciferin types attributed to amino acids with various properties. The three major solvent-exposed loops are represented by HVR-I, HVR-III (a.k.a. the extended loop region) and HVR-IV and were replete with charged (glutamate, arginine and lysine) and polar (asparagine, glutamine and serine) amino acids. The CsCruC paralogues had the longest HVR-II regions, although this was much shorter than that found within *A. thaliana* CruC (Suppl. Fig. S6). Hexamer formation proceeds by interaction of the interchain disulphide bond-containing (IE) faces of two trimers after proteolytic processing at the β-cleavage site (Fig. 5) which permits movement of HVR-IV to the periphery of the protein and exposes the trimer-interacting regions. The four trimer-interacting regions were highly conserved in CsCruA, CsCruB and CsCruC (Fig. 5); however, several differences were noted in CsCruD, in particular in polar and charged residues important for hydrogen bond and ionic interactions between the trimers (Adachi et al. 2001, 2003; Tandang-Silvas et al. 2010). This suggests that while CsCruD may form trimers, its participation may lead to hexamers with less stable structures. HVR-II and HVR-V remain on the IE face and their high degree of variability contributes to the lower degree of evolutionary conservation, as well as variation in electrostatic potential and hydrophobicity (Fig. 4) which may also influence the stability of trimer-trimer interactions. Additional cysteine residues not predicted to be involved in inter- or intrachain disulphide bond formation were present in CsCruB and CsCruC (Fig. 5; Suppl. Fig. S6), which could promote interactions with other proteins/molecules or inter-subunit disulphide bond exchanges (Shimada et al. 1980; Inquello et al. 1993).

In the context of functional properties (i.e. the properties that proteins confer in multi-component systems), the physicochemical properties of native cruciferin are directly related to the nature of the surface-exposed residues (Withana-Gamage et al. 2013a, 2013b, 2015, 2020). CsCruD had the highest percentage of negatively charged amino acids (11.1%; total net charge − 14) and the lowest isoelectric point (4.99) of the *C. sativa*, *A. thaliana* and *B. napus* cruciferins (Table 6). CsCruD had a grand average hydropathicity (GRAVY) value of − 0.375, making it the least hydrophilic of all the cruciferins examined, while CsCruC was the most hydrophilic cruciferin (GRAVY = − 0.627) and was comparable to *A. thaliana* CruC. This suggests that CsCruC would be the most soluble in aqueous solution, while CsCruD would be the least soluble. The spatial arrangement of hydrophilic and hydrophobic residues on the exposed surfaces was also markedly different for the cruciferin types. The intrachain disulphide bond-containing (IA) faces of CsCruB and CsCruC had negatively charged peripheries with a positively charged central region, while the IA face of CsCruD was dominated by negatively charged amino acids (Fig. 4). As expected, the IA face of all cruciferins were generally hydrophilic; however, in CsCruA, CsCruB and CsCruC, hydrophobic residues tended to occur in small clusters, while those in CsCruD were more evenly distributed across its surface (Fig. 4).

**Table 5:**
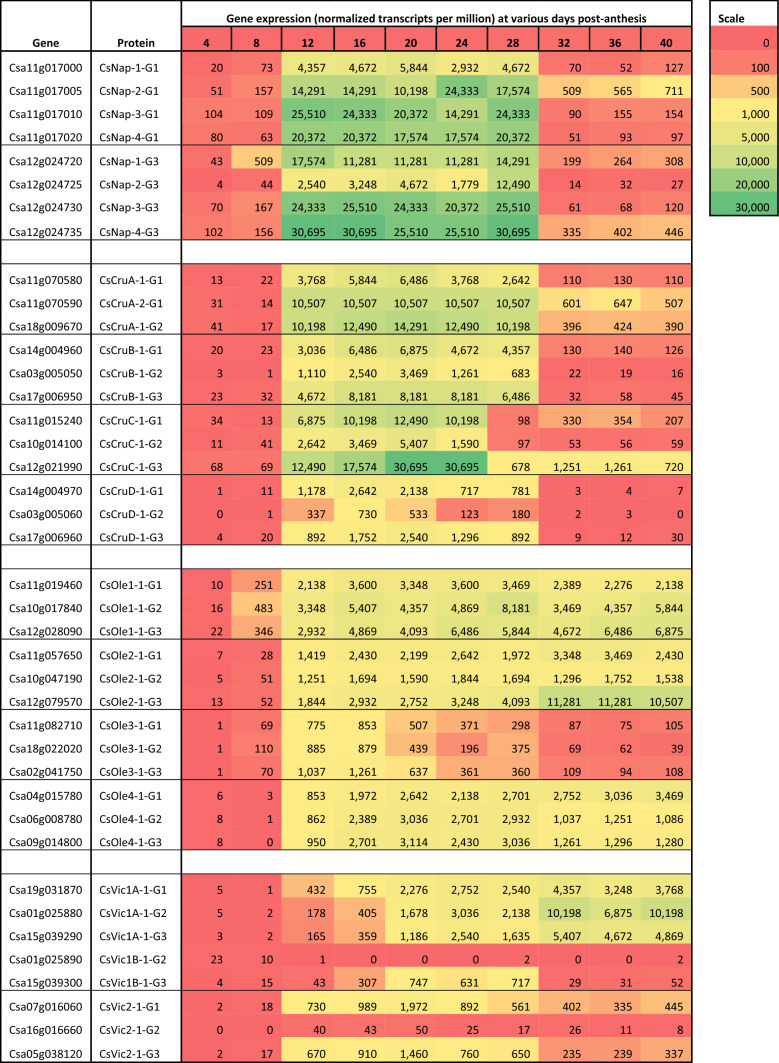
Expression of *C. sativa* cv. DH55 genes encoding seed storage proteins

**Table 6:** Properties of *B. napus*, *A. thaliana* and *C. sativa* cruciferins

Property*	Cruciferin							
	*B. napus* 3KGL	*A. thaliana*			*C. sativa*			
		CRA	CRB	CRC	CruA	CruB	CruC	CruD
Protomer								
Formula	C_2247_H_3515_N_671_O_696_S_8_	C_2200_H_3442_N_658_O_670_S_8_	C_2118_H_3322_ N_616_O_636_S_15_	C_2436_H_3814_ N_734_O_756_S_12_	C_2178_H_3408_N_644_O_664_S_7_	C_2116_H_3310_ N_618_O_647_S_16_	C_2288_H_3610_ N_688_O_710_S_13_	C_1975_H_3057_ N_565_O_612_S_10_
Amino acids	466	449	432	501	445	435	469	405
M_r_ (kDa)	51.3	50.1	48.1	55.9	49.5	48.3	52.5	44.8
pI	6.6	7.26	6.36	6.36	6.41	5.96	6.51	4.99
Negative residues	43 (9.2%)	45 (10.0%)	42 (9.7%)	45 (9.0%)	46 (10.3%)	40 (9.2%)	45 (9.6%)	45 (11.1%)
Positive residues	41 (8.8%)	45 (10.0%)	39 (9.0%)	42 (8.4%)	43 (9.7%)	34 (7.8%)	43 (9.2%)	32 (7.9%)
GRAVY	− 0.557	− 0.562	− 0.432	− 0.691	− 0.487	− 0.46	− 0.627	− 0.375
Total charge	0	− 2	− 5	− 2	− 5	− 8	− 1	− 14
Trimer								
Total pockets	−	228	270	283	221	247	260	214
Central pocket volume (Å^3^)	−	17,419.4	9959.7	5092.9	8709.5	17,173.2	4178.7	3070.3
Central pocket area (Å)	−	10,024.4	6755.4	3133.1	4799.9	8821.3	2369.6	1741.2
Central pocket circumference (Å)	−	896.1	449.1	218.4	251.9	733.9	86.4	14.4
Central pocket openings	−	28	15	1	6	15	1	1
Central pocket mouth area (Å)	−	1695.1	762.2	577.7	624.8	1856.0	275.5	12.8

*M_*r*_ molecular weight, *pI* isoelectric point, total number of negatively charged residues (Asp + Glu), total number of positively charged residues (Arg + Lys), GRAVY–grand average hydropathy value according to Kyte and Doolittle (1982). Negative scores indicate increasing hydrophilicity, positive scores indicate increasing hydrophobicity

Phosphorylation of cruciferin was first noted in *A. thaliana* (Wan et al. 2007) and now appears to be a general occurrence in seed and vegetative storage proteins (Mouzo et al. 2018). Phosphorylation of serine, threonine or tyrosine was predicted to occur on 23–37 residues in the *C. sativa* cruciferin forms (Fig. 5; Suppl. Fig. S6; Suppl. Table S9). These were predicted to occur within the core structure, on the IE face and on the surface (IA face, periphery and in solvent accessible cavities) (Fig. 4) indicating that this post-translational modification may influence protein folding, subunit interactions, as well as surface-active properties.

An important property for proteins used as food ingredients is their ability to bind/sequester small molecules, such as pigments and flavours. This is related to number, size and chemical properties of pockets in the tertiary and quaternary structure that are accessible to the solvent. The total number of pockets (1.4 Å probe) in the *C. sativa* cruciferin trimers ranged from 214 (CsCruD) to 260 (CsCruC) (Table 6). A larger central pocket forms when the protomers associate to form the trimer and is accessible via an opening on the IE face. The size of this pocket size is also a measure of packing efficiency. In homomeric form, CsCruB had the largest pocket volume (17,173.2 Å^3^), twice that of CsCruA (8709.5 Å^3^) and four-five times that of CsCruC (4178.7 Å^3^) and CruD (3070.3 Å^3^) (Table 6). The CruB central pocket was also the most accessible with a mouth opening area of 1856.0 Å^2^ with 15 individual openings (orientations through which a water molecule may pass). CsCruD had the smallest pocket volume and was also the least accessible with a mouth opening area of only 12.8 Å^2^ with one opening. CsCruC had a similar pocket volume with a wider mouth area 275.5 Å^2^; however, this was accessible by only a single opening.

## Discussion

Current interest in *C. sativa* is mainly centred around oil and its use in bio-fuels (Li and Mupondwa 2014) or as a supplement in animal and fish feeds (Hixson et al. 2014; Hixson and Parrish 2014); however, utilisation of its meal protein (Colombini et al. 2014; Pekel et al. 2015; Hixson et al. 2016a, 2016b) will be necessary to achieve maximal commercial exploitation and valorization. *C. sativa* seed comprises about 43% protein (Zubr 2003), but little or nothing is known about other closely related *Camelina* species. The current study established that *Camelina* species exhibit different seed protein profiles and these differences can separate genotypes representing them. The percent protein in defatted meal also varied between species and less so between lines within the same species. Meal from *C. microcarpa* had the lowest protein content, 31%, while meal from *C. hispida hispida*, *C. laxa*, *C. rumelica transcapida* and some *C. rumelica rumelica* and *C. sativa* lines all reached or exceeded 40%. This is slightly higher than the 38% reported for canola meal, but less than the 46% for soybean meal (So and Duncan 2021), which have been bred for oil and protein content, respectively.

Lysine and methionine are not synthesised de novo by animals and must be obtained from their diets. These are also limiting in wholly plant-based diets and are often added as supplements to feeds used for monogastric animals, such as fish (Wilson and Halver 1986), poultry (Kidd et al. 1998) and swine (Brinegar et al. 1950). Meals derived from Cruciferous oilseeds generally have higher levels of lysine and methionine than cereals, with *C. sativa* exhibiting a reasonably-balanced essential amino acid profile. Like protein content, amino acid content in the meal also varied between *Camelina* species. Lysine levels were lowest in meal from *C. rumelica rumelica* (4.77%) and highest in most *C. sativa* lines (up to 5.74% in line 1063). Histidine was highest in the meal from *C. rumelica rumelica* (4.77% in line 1034), almost twice that found in meals from any of the other *Camelina* species. Interestingly, the amino acid composition of the two major seed proteins, napin and cruciferin, would account for only about one-half of the total lysine and histidine (Suppl. Table S10) indicating that unincorporated/free amino acids or other proteins of lesser abundance are major contributors to the overall meal amino acid profile. Variation in meal amino acid composition was observed between lines within a species. Methionine and cysteine were highest in meal from *C. rumelica rumelica* lines 609 (2.89%) and 247 (9.32%), respectively, but lowest in *C. rumelica rumelica* line 1034. Serine content was highest in meal from *C. sativa* line 605 meal (5.39%), but lowest in line 252 (4.43%). Threonine was also lowest in meal from *C. sativa* line 1662 (3.83%); however, other *C. sativa* lines exceeded 4.5% similar to other *Camelina* species. This analysis clearly demonstrates that variation among *C. sativa* lines and in related species exists, which could be accessed to develop lines producing meals with amino acid compositions that are better suited for monogastric diets. However, it remains to be demonstrated whether adequate levels of several or all limiting essential amino acids can be achieved in the same genetic background as regulatory mechanisms governing carbon/nitrogen partitioning may not permit this. With respect to essential amino acids, canola meal has comparable levels of histidine (3.39%), isoleucine (3.47), leucine (6.19%), phenyalanine (4.06%), and threonine (4.27), slightly lower levels of lysine (5.92%), and lower levels of cysteine (2.29%), methionine (1.94%), tyrosine (2.50%) and valine (4.97) (Wanasundara et al. 2016) than were found in lines from the various *Camelina* species examined here. It should be noted that differences in analytical techniques must be considered in such comparisons and significant variation in protein and amino acid content has been reported in canola meal from different crushing plants (Le Thanh et al. 2019).

For the most part, variation in seed protein profile between *C. sativa* lines was limited in the 187 accessions examined, which is in keeping with genotypic analyses (Singh et al. 2015; Luo et al. 2019; Chaudhary et al. 2020). This may be attributed to the notion that *C. sativa* is a recent allopolyploid where most homeologous genes are expressed and little sub-genome fractionation has occurred (Kagale et al. 2014, 2016). Despite this, most of the lines could be placed into one of three classes based on differences in the electrophoretic profile of high molecular weight proteins consisting mainly of cruciferin. *C. sativa* possesses 12 genes encoding cruciferin, with each of the three sub-genomes having a contingent of homeologues (Kagale et al. 2014). The 12 *C. sativa* cruciferins are phylogenetically related to the four *A. thaliana* cruciferins, namely AtCRA (At5g44120), AtCRB (At1g03880), AtCRC (At4g28520), and AtCRD (At1g03890). A *CRA* orthologue is not present on any of the *C. sativa* sub-genome G3 chromosomes; however, a tandem duplication occurs on G1 chromosome 11 yielding *CsCruA-1-G1* (Csa11g070580) and *CsCruA-2-G1* (Csa11g070590). Interestingly, the *CsCruB* and *CsCruD* paralogues are also closely linked on each of the sub-genomes, similar to that in *A. thaliana*, even though they are the two most distantly related cruciferins. This signature is suggestive of a duplication event that occurred in a progenitor genome with sufficient time for divergence before the original triplication event that gave rise to the ancestor of both *A. thaliana* and *C. sativa*. It is especially interesting that this arrangement has been maintained through subsequent genome polyploidization and fractionation events in *C. sativa*. The situation with the organisation of napin genes is equally compelling. The *A. thaliana* genome contains 5 genes encoding napin, four of these are linked in tandem on chromosome 4 and are closely related, while the fifth is present on chromosome 5. *Camelina sativa* also has two clusters of four napin genes, one on G1 and the other on G3; no napin genes occur on any G2 chromosomes. This arrangement, however, appears to be coincidental as phylogenetic comparisons between the genes within the *A. thaliana* and *C. sativa* napin clusters indicate that each evolved through a different duplicative route. When the napin gene or gene cluster was lost from G2 might be resolved by examination of genomes from other *Camelina* species (Chaudhary et al. 2020). Two genes encoding vicilin 1 lie in tandem on both G2 and G3, while a single gene is present on G1. This genomic arrangement and phylogenetic analysis suggest that these two sub-genomes are more closely related to one another than to G1, a notion which is supported by genotypic data (Chaudhary et al. 2020).

RNA-Seq analysis of seven *C. sativa* lines revealed that the same homeologues/paralogues encoding napins, oleosins and vicilins were expressed and at similar levels; however, the expression of cruciferin homeologues/paralouges differed widely between lines in some instances. In the *C. sativa* type strain DH55, genes encoding cruciferins were mainly expressed from the 12th to the 28th day post-anthesis. The general pattern of expression according to transcript levels was *CsCruC* > *CsCruA* > *CsCruB* > *CsCruD*. This same relative expression profile is also present in *A. thaliana* (TAIR; https://www.arabidopsis.org/) and, thus, appeared to be evolutionarily conserved and possibly of functional importance. However, upon examination of six additional *C. sativa* lines, only CN45816 shared this pattern with DH55. In the other five lines, genes encoding CsCruA and CsCruB contributed the majority of the transcripts with those encoding CsCruC and CsCruD providing only a minor component. These general patterns were confirmed by proteomic analysis. The differences in the abundance of cruciferin isoforms/types between the lines has significant consequences as cruciferin is the most abundant seed storage protein and, as such, is the principal contributor to the physiochemical and nutritional properties of meal protein. Cruciferin is a hexamer with the degree of heterogeneity determined by the stoichiometry of the various protomers. While this serves to homogenise the physiochemical properties of individual cruciferin types (Withana-Gamage et al. 2011, 2013a, 2013b, 2015, 2020), it is conceivable that *C. sativa* lines could be selected that produce meals or globulin isolates with properties suited to specific applications. Reduction in the expression of the entire napin gene family via RNA interference (Nguyen et al. 2013) and targeted disruption of homeologous genes encoding CsCruC (Lyzenga et al. 2019) have been successful in altering *C. sativa* seed protein composition and, by inference, the physiochemical properties of the meal. Vicilins are similar to cruciferins in that they are bicupin-domain globulins; however, they remain as trimers similar to the 7S globulins in legumes (Shewry et al. 1995). In *A. thaliana*, the genes encoding vicilins 1 and 2 are expressed at low levels during seed development (TAIR; https://www.arabidopsis.org/) and these proteins likely contribute little to seed protein composition. Conversely, genes encoding CsVic1A were expressed at levels comparable to those encoding CsCruB and moreso than those encoding CsCruC in many of the *C. sativa* lines. Interestingly, neither the *A. thaliana* nor the *C. sativa* vicilin 2 proteins were predicted to contain a signal peptide and are, therefore, unlikely to be deposited within protein storage vacuoles.

Given the sequence and structural similarity between *A. thaliana and C. sativa* cruciferin isoforms, it may be assumed that they share similar physiochemical properties. Cruciferins and other 11S/12S globulins contain two conserved β-barrel or cupin domains; however, the five hypervariable regions confer different properties on individual isoforms (Tandang-Silvas et al. 2010). As noted with *A. thaliana* cruciferins (Withana-Gamage et al. 2011), HVR-I and HVR-III are located on the solvent-exposed surface of the IA face in the hexamer, while HVR-IV moves to the periphery after cleavage at the β-site. In both *A. thaliana* and *C. sativa*, CruC possesses an extended, glutamine-rich, HVR-II within the alpha subunit. In specialised *A. thaliana* lines producing homomeric cruciferins, AtCRC was found to form a compact and less hydrophobic hexamer than either homomeric AtCRA or AtCRB. This resulted in increased thermostability and reduced susceptibility to hydrolysis by pepsin, but altered its ability to form heat-induced gels and to stabilise oil-in-water emulsions (Withana-Gamage et al. 2013a, 2013b, 2015, 2020). Furthermore, reduced proteolytic susceptibility is one of several factors that contribute to the antigenic potential of cupin-like proteins (Mills et al. 2002) making elimination of CsCruC in *C. sativa* an attractive goal (Lyzenga et al. 2019). Homomeric AtCRA and AtCRB formed strong heat-induced gels (Withana-Gamage et al. 2015) and possessed good ability to stabilise oil-in-water emulsions over a wide pH range (Withana-Gamage et al. 2020). Structural features that facilitate flavour or small molecule binding, such as the size of the central pocket and mouth opening (Guichard 2006), were most prominent in CsCruB followed by CsCruA. CsCruD has an unusual HVR-IV that is rich in arginine rather than glutamine residues as in other cruciferin types. Its IA face (solvent-exposed) is dominated by negatively charged amino acids with a more even distribution of hydrophobic residues suggesting that it may possess unique properties. CruD also presents an enigma. It is expressed at very low levels compared to genes encoding other cruciferins. It also possesses alterations in polar and charged residues important for interaction between trimers (Adachi et al. 2001, 2003; Tandang-Silvas et al. 2010), suggesting that it may destabilise hexamers when present. While this may seem counter-intuitive, seed storage proteins must be both stable and be rapidly mobilised during seed germination. Following imbibition, globulin mobilisation is achieved through the sequential hydrolysis of a limited number of internal sites by metallo-endopeptidases followed by a more general degradation by cysteine proteases (Muntz et al. 2001; Tan-Wilson and Wilson 2011). Slight structural instability introduced by CruD may assist in this process when this minor isoform is present and may explain why it remains in *A. thaliana* and *C. sativa*, as well as in other Brassicaceae.

In conclusion, the wealth of information on seed protein diversity in *Camelina* species provided in this work will initially be useful in breeding/engineering lines with higher protein content and amino acid profiles suitable for animal and, possibly, human diets. The plant protein industry is already moving in this direction and beyond, with particular interest in purified protein isolates, mainly albumins (napins) and globulins (cruciferins), for specific food applications (So and Duncan 2021). In the future, knowledge of the genes and their expression patterns that underlie the protein profiles will permit the creation of specialised *C. sativa* lines that, for example, produce homogeneous cruciferins with properties tailored to specific applications. Indeed, targeted disruption of entire cruciferin gene families, notably CsCruC, has already been demonstrated in *C. sativa* (Lyzenga et al. 2019). It is only a matter of time before this is applied to other oilseed species.

### *Author contribution statement*

DD, SM, IAP, AH and JW conceived, designed and funded the research. BG, MH and SP conducted experiments. CC analysed data. DD, IAP and JW wrote the manuscript. All authors read and approved the manuscript.

## Supplementary Information

Below is the link to the electronic supplementary material.

Supplementary file1 (DOCX 90 KB)

Supplementary file2 (XLSX 31 KB)

Supplementary file3 (XLSX 25 KB)

Supplementary file4 (DOCX 7072 KB)

Supplementary file5 (XLSX 25 KB)

Supplementary file6 (XLSX 51 KB)

Supplementary file7 (XLSX 76 KB)

Supplementary file8 (XLSX 29 KB)

Supplementary file9 (XLSX 22 KB)

Supplementary file10 (XLSX 18 KB)

Supplementary file11 (PDF 235 KB)

Supplementary file12 (PDF 609 KB)

Supplementary file13 (PDF 68 KB)

Supplementary file14 (PDF 68 KB)

Supplementary file15 (PDF 83 KB)

Supplementary file16 (PDF 74 KB)

Supplementary file17 (PDF 347 KB)

Supplementary file18 (PDF 255 KB)

Supplementary file19 (PDF 3533 KB)

## Abbreviations

daa: Days after anthesis
G1: Sub-genome I
G2: Sub-genome II
G3: Sub-genome III
HVR: Hypervariable region
IA: Intrachain disulphide bond-containing
IE: Interchain disulphide bond-containing
PGRC: Plant Gene Resources Center
RMSD: Root mean square difference

## Declarations

### Data availability statement

The datasets generated during and/or analysed during the current study are deposited in publicly available repositories as indicated or available from the corresponding author upon reasonable request.
