# Supplementary material for: Genetic variation and structural diversity in major seed proteins among and within Camelina species
Source: Planta. 2022 Oct 6;256(5):93. doi: 10.1007/s00425-022-03998-w (PMC9537204; doi:10.1007/s00425-022-03998-w)
Supplement: Supplementary file 1 — Supplementary file1 (DOCX 90 KB) [file 425_2022_3998_MOESM1_ESM.docx]

**Supplemental Table S10**. Properties and amino acid composition of *C. sativa* mature cruciferins and napins.

Csa11g070580_CruA-1-G1 (mature)

| **Analysis** | **Entire Protein** |
| --- | --- |
| Length | 446 aa |
| Molecular Weight | 49571.57 |
| 1 microgram = | 20.173 pMoles |
| Molar Extinction coefficient | 40450 |
| 1 A[280] corr. to | 1.23 mg/ml |
| A[280] of 1 mg/ml | 0.82 AU |
| Isoelectric Point | 6.41 |
| Charge at pH 7 | -2.34 |

| **Amino Acid(s)** | **Number count** | **% by weight** | **% by frequency** |
| --- | --- | --- | --- |
| Charged (RKHYCDE) | 112 | 30.03 | 25.11 |
| Acidic (DE) | 46 | 11.24 | 10.31 |
| Basic (KR) | 43 | 12.42 | 9.64 |
| Polar (NCQSTY) | 130 | 29.75 | 29.15 |
| Hydrophobic (AILFWV) | 150 | 32.87 | 33.63 |
| A Ala | 31 | 4.80 | 6.95 |
| C Cys | 4 | 0.84 | 0.90 |
| D Asp | 21 | 4.85 | 4.71 |
| E Glu | 25 | 6.39 | 5.61 |
| F Phe | 21 | 6.02 | 4.71 |
| G Gly | 37 | 4.82 | 8.30 |
| H His | 10 | 2.69 | 2.24 |
| I Ile | 21 | 4.78 | 4.71 |
| K Lys | 12 | 3.05 | 2.69 |
| L Leu | 35 | 7.97 | 7.85 |
| M Met | 3 | 0.78 | 0.67 |
| N Asn | 26 | 5.96 | 5.83 |
| P Pro | 26 | 5.20 | 5.83 |
| Q Gln | 43 | 10.91 | 9.64 |
| R Arg | 31 | 9.38 | 6.95 |
| S Ser | 30 | 5.47 | 6.73 |
| T Thr | 18 | 3.72 | 4.04 |
| V Val | 37 | 7.53 | 8.30 |
| W Trp | 5 | 1.77 | 1.12 |
| Y Tyr | 9 | 2.83 | 2.02 |

Csa11g070590_CruA-2-G1 (mature)

| **Analysis** | **Entire Protein** |
| --- | --- |
| Length | 446 aa |
| Molecular Weight | 49576.59 |
| 1 microgram = | 20.171 pMoles |
| Molar Extinction coefficient | 40450 |
| 1 A[280] corr. to | 1.23 mg/ml |
| A[280] of 1 mg/ml | 0.82 AU |
| Isoelectric Point | 6.36 |
| Charge at pH 7 | -2.42 |

| **Amino Acid(s)** | **Number count** | **% by weight** | **% by frequency** |
| --- | --- | --- | --- |
| Charged (RKHYCDE) | 111 | 29.76 | 24.89 |
| Acidic (DE) | 46 | 11.24 | 10.31 |
| Basic (KR) | 43 | 12.42 | 9.64 |
| Polar (NCQSTY) | 131 | 30.02 | 29.37 |
| Hydrophobic (AILFWV) | 150 | 32.87 | 33.63 |
| A Ala | 31 | 4.80 | 6.95 |
| C Cys | 4 | 0.84 | 0.90 |
| D Asp | 21 | 4.85 | 4.71 |
| E Glu | 25 | 6.39 | 5.61 |
| F Phe | 21 | 6.02 | 4.71 |
| G Gly | 37 | 4.82 | 8.30 |
| H His | 9 | 2.42 | 2.02 |
| I Ile | 21 | 4.78 | 4.71 |
| K Lys | 12 | 3.05 | 2.69 |
| L Leu | 35 | 7.97 | 7.85 |
| M Met | 3 | 0.78 | 0.67 |
| N Asn | 26 | 5.96 | 5.83 |
| P Pro | 26 | 5.20 | 5.83 |
| Q Gln | 44 | 11.17 | 9.87 |
| R Arg | 31 | 9.38 | 6.95 |
| S Ser | 29 | 5.29 | 6.50 |
| T Thr | 19 | 3.93 | 4.26 |
| V Val | 37 | 7.53 | 8.30 |
| W Trp | 5 | 1.77 | 1.12 |
| Y Tyr | 9 | 2.83 | 2.02 |

Csa18g009670_CruA-1-G2 (mature)

| **Analysis** | **Entire Protein** |
| --- | --- |
| Length | 446 aa |
| Molecular Weight | 49705.72 |
| 1 microgram = | 20.118 pMoles |
| Molar Extinction coefficient | 40450 |
| 1 A[280] corr. to | 1.23 mg/ml |
| A[280] of 1 mg/ml | 0.81 AU |
| Isoelectric Point | 6.41 |
| Charge at pH 7 | -2.34 |

| **Amino Acid(s)** | **Number count** | **% by weight** | **% by frequency** |
| --- | --- | --- | --- |
| Charged (RKHYCDE) | 112 | 29.91 | 25.11 |
| Acidic (DE) | 46 | 11.21 | 10.31 |
| Basic (KR) | 43 | 12.35 | 9.64 |
| Polar (NCQSTY) | 134 | 30.60 | 30.04 |
| Hydrophobic (AILFWV) | 148 | 32.49 | 33.18 |
| A Ala | 29 | 4.48 | 6.50 |
| C Cys | 4 | 0.84 | 0.90 |
| D Asp | 21 | 4.84 | 4.71 |
| E Glu | 25 | 6.37 | 5.61 |
| F Phe | 21 | 6.01 | 4.71 |
| G Gly | 36 | 4.68 | 8.07 |
| H His | 10 | 2.69 | 2.24 |
| I Ile | 21 | 4.77 | 4.71 |
| K Lys | 13 | 3.29 | 2.91 |
| L Leu | 35 | 7.95 | 7.85 |
| M Met | 3 | 0.78 | 0.67 |
| N Asn | 26 | 5.95 | 5.83 |
| P Pro | 25 | 4.99 | 5.61 |
| Q Gln | 45 | 11.39 | 10.09 |
| R Arg | 30 | 9.05 | 6.73 |
| S Ser | 30 | 5.46 | 6.73 |
| T Thr | 20 | 4.13 | 4.48 |
| V Val | 37 | 7.51 | 8.30 |
| W Trp | 5 | 1.77 | 1.12 |
| Y Tyr | 9 | 2.82 | 2.02 |

Csa17g006950_CruB-1-G1 (mature)

| **Analysis** | **Entire Protein** |
| --- | --- |
| Length | 436 aa |
| Molecular Weight | 48380.86 |
| 1 microgram = | 20.669 pMoles |
| Molar Extinction coefficient | 43820 |
| 1 A[280] corr. to | 1.10 mg/ml |
| A[280] of 1 mg/ml | 0.91 AU |
| Isoelectric Point | 5.96 |
| Charge at pH 7 | -5.36 |

| **Amino Acid(s)** | **Number count** | **% by weight** | **% by frequency** |
| --- | --- | --- | --- |
| Charged (RKHYCDE) | 97 | 26.41 | 22.25 |
| Acidic (DE) | 40 | 10.12 | 9.17 |
| Basic (KR) | 34 | 9.99 | 7.80 |
| Polar (NCQSTY) | 137 | 32.08 | 31.42 |
| Hydrophobic (AILFWV) | 139 | 31.64 | 31.88 |
| A Ala | 26 | 4.12 | 5.96 |
| C Cys | 6 | 1.29 | 1.38 |
| D Asp | 14 | 3.31 | 3.21 |
| E Glu | 26 | 6.80 | 5.96 |
| F Phe | 19 | 5.58 | 4.36 |
| G Gly | 39 | 5.21 | 8.94 |
| H His | 10 | 2.76 | 2.29 |
| I Ile | 20 | 4.67 | 4.59 |
| K Lys | 11 | 2.86 | 2.52 |
| L Leu | 37 | 8.63 | 8.49 |
| M Met | 10 | 2.65 | 2.29 |
| N Asn | 34 | 7.99 | 7.80 |
| P Pro | 26 | 5.32 | 5.96 |
| Q Gln | 43 | 11.18 | 9.86 |
| R Arg | 23 | 7.13 | 5.28 |
| S Ser | 24 | 4.49 | 5.50 |
| T Thr | 23 | 4.87 | 5.28 |
| V Val | 31 | 6.46 | 7.11 |
| W Trp | 6 | 2.18 | 1.38 |
| Y Tyr | 7 | 2.25 | 1.61 |

Csa14g004960_CruB-1-G2 (mature)

| **Analysis** | **Entire Protein** |
| --- | --- |
| Length | 434 aa |
| Molecular Weight | 48164.74 |
| 1 microgram = | 20.762 pMoles |
| Molar Extinction coefficient | 43820 |
| 1 A[280] corr. to | 1.10 mg/ml |
| A[280] of 1 mg/ml | 0.91 AU |
| Isoelectric Point | 6.50 |
| Charge at pH 7 | -1.54 |

| **Amino Acid(s)** | **Number count** | **% by weight** | **% by frequency** |
| --- | --- | --- | --- |
| Charged (RKHYCDE) | 97 | 26.52 | 22.35 |
| Acidic (DE) | 39 | 9.88 | 8.99 |
| Basic (KR) | 37 | 10.87 | 8.53 |
| Polar (NCQSTY) | 136 | 31.93 | 31.34 |
| Hydrophobic (AILFWV) | 140 | 31.97 | 32.26 |
| A Ala | 27 | 4.30 | 6.22 |
| C Cys | 6 | 1.30 | 1.38 |
| D Asp | 15 | 3.57 | 3.46 |
| E Glu | 24 | 6.31 | 5.53 |
| F Phe | 19 | 5.61 | 4.38 |
| G Gly | 38 | 5.10 | 8.76 |
| H His | 8 | 2.22 | 1.84 |
| I Ile | 20 | 4.69 | 4.61 |
| K Lys | 13 | 3.40 | 3.00 |
| L Leu | 38 | 8.91 | 8.76 |
| M Met | 10 | 2.67 | 2.30 |
| N Asn | 34 | 8.03 | 7.83 |
| P Pro | 25 | 5.14 | 5.76 |
| Q Gln | 42 | 10.97 | 9.68 |
| R Arg | 24 | 7.47 | 5.53 |
| S Ser | 25 | 4.69 | 5.76 |
| T Thr | 22 | 4.68 | 5.07 |
| V Val | 30 | 6.28 | 6.91 |
| W Trp | 6 | 2.19 | 1.38 |
| Y Tyr | 7 | 2.26 | 1.61 |

Csa03g005050_CruB-1-G3 (mature)

| **Analysis** | **Entire Protein** |
| --- | --- |
| Length | 434 aa |
| Molecular Weight | 48178.77 |
| 1 microgram = | 20.756 pMoles |
| Molar Extinction coefficient | 43820 |
| 1 A[280] corr. to | 1.10 mg/ml |
| A[280] of 1 mg/ml | 0.91 AU |
| Isoelectric Point | 6.50 |
| Charge at pH 7 | -1.54 |

| **Amino Acid(s)** | **Number count** | **% by weight** | **% by frequency** |
| --- | --- | --- | --- |
| Charged (RKHYCDE) | 97 | 26.52 | 22.35 |
| Acidic (DE) | 39 | 9.87 | 8.99 |
| Basic (KR) | 37 | 10.86 | 8.53 |
| Polar (NCQSTY) | 136 | 31.93 | 31.34 |
| Hydrophobic (AILFWV) | 140 | 31.99 | 32.26 |
| A Ala | 27 | 4.30 | 6.22 |
| C Cys | 6 | 1.30 | 1.38 |
| D Asp | 15 | 3.57 | 3.46 |
| E Glu | 24 | 6.31 | 5.53 |
| F Phe | 19 | 5.61 | 4.38 |
| G Gly | 38 | 5.10 | 8.76 |
| H His | 8 | 2.22 | 1.84 |
| I Ile | 21 | 4.92 | 4.84 |
| K Lys | 13 | 3.39 | 3.00 |
| L Leu | 38 | 8.90 | 8.76 |
| M Met | 10 | 2.67 | 2.30 |
| N Asn | 34 | 8.02 | 7.83 |
| P Pro | 25 | 5.14 | 5.76 |
| Q Gln | 42 | 10.96 | 9.68 |
| R Arg | 24 | 7.47 | 5.53 |
| S Ser | 25 | 4.69 | 5.76 |
| T Thr | 22 | 4.68 | 5.07 |
| V Val | 29 | 6.07 | 6.68 |
| W Trp | 6 | 2.19 | 1.38 |
| Y Tyr | 7 | 2.26 | 1.61 |

Csa11g015240_CruC-1-G1 (mature)

| **Analysis** | **Entire Protein** |
| --- | --- |
| Length | 470 aa |
| Molecular Weight | 52640.60 |
| 1 microgram = | 18.997 pMoles |
| Molar Extinction coefficient | 46970 |
| 1 A[280] corr. to | 1.12 mg/ml |
| A[280] of 1 mg/ml | 0.89 AU |
| Isoelectric Point | 6.51 |
| Charge at pH 7 | -1.52 |

| **Amino Acid(s)** | **Number count** | **% by weight** | **% by frequency** |
| --- | --- | --- | --- |
| Charged (RKHYCDE) | 115 | 29.33 | 24.47 |
| Acidic (DE) | 45 | 10.45 | 9.57 |
| Basic (KR) | 43 | 11.71 | 9.15 |
| Polar (NCQSTY) | 155 | 34.51 | 32.98 |
| Hydrophobic (AILFWV) | 141 | 29.08 | 30.00 |
| A Ala | 26 | 3.79 | 5.53 |
| C Cys | 5 | 0.99 | 1.06 |
| D Asp | 17 | 3.70 | 3.62 |
| E Glu | 28 | 6.74 | 5.96 |
| F Phe | 14 | 3.79 | 2.98 |
| G Gly | 45 | 5.53 | 9.57 |
| H His | 8 | 2.03 | 1.70 |
| I Ile | 29 | 6.23 | 6.17 |
| K Lys | 12 | 2.87 | 2.55 |
| L Leu | 33 | 7.09 | 7.02 |
| M Met | 8 | 1.95 | 1.70 |
| N Asn | 30 | 6.49 | 6.38 |
| P Pro | 24 | 4.52 | 5.11 |
| Q Gln | 63 | 15.07 | 13.40 |
| R Arg | 31 | 8.84 | 6.60 |
| S Ser | 25 | 4.30 | 5.32 |
| T Thr | 18 | 3.51 | 3.83 |
| V Val | 34 | 6.52 | 7.23 |
| W Trp | 5 | 1.67 | 1.06 |
| Y Tyr | 14 | 4.15 | 2.98 |

Csa10g014100_CruC-1-G2 (mature)

| **Analysis** | **Entire Protein** |
| --- | --- |
| Length | 461 aa |
| Molecular Weight | 51689.57 |
| 1 microgram = | 19.346 pMoles |
| Molar Extinction coefficient | 46850 |
| 1 A[280] corr. to | 1.10 mg/ml |
| A[280] of 1 mg/ml | 0.91 AU |
| Isoelectric Point | 8.30 |
| Charge at pH 7 | 2.57 |

| **Amino Acid(s)** | **Number count** | **% by weight** | **% by frequency** |
| --- | --- | --- | --- |
| Charged (RKHYCDE) | 113 | 29.49 | 24.51 |
| Acidic (DE) | 42 | 9.91 | 9.11 |
| Basic (KR) | 44 | 12.22 | 9.54 |
| Polar (NCQSTY) | 151 | 34.26 | 32.75 |
| Hydrophobic (AILFWV) | 137 | 28.84 | 29.72 |
| A Ala | 25 | 3.71 | 5.42 |
| C Cys | 4 | 0.81 | 0.87 |
| D Asp | 17 | 3.77 | 3.69 |
| E Glu | 25 | 6.13 | 5.42 |
| F Phe | 14 | 3.86 | 3.04 |
| G Gly | 45 | 5.63 | 9.76 |
| H His | 9 | 2.33 | 1.95 |
| I Ile | 28 | 6.12 | 6.07 |
| K Lys | 12 | 2.92 | 2.60 |
| L Leu | 32 | 7.00 | 6.94 |
| M Met | 8 | 1.99 | 1.74 |
| N Asn | 30 | 6.61 | 6.51 |
| P Pro | 24 | 4.61 | 5.21 |
| Q Gln | 61 | 14.86 | 13.23 |
| R Arg | 32 | 9.29 | 6.94 |
| S Ser | 25 | 4.38 | 5.42 |
| T Thr | 17 | 3.38 | 3.69 |
| V Val | 33 | 6.45 | 7.16 |
| W Trp | 5 | 1.70 | 1.08 |
| Y Tyr | 14 | 4.23 | 3.04 |

Csa12g021990_CruC-1-G3 (mature)

| **Analysis** | **Entire Protein** |
| --- | --- |
| Length | 470 aa |
| Molecular Weight | 53096.88 |
| 1 microgram = | 18.833 pMoles |
| Molar Extinction coefficient | 48130 |
| 1 A[280] corr. to | 1.10 mg/ml |
| A[280] of 1 mg/ml | 0.91 AU |
| Isoelectric Point | 6.36 |
| Charge at pH 7 | -2.42 |

| **Amino Acid(s)** | **Number count** | **% by weight** | **% by frequency** |
| --- | --- | --- | --- |
| Charged (RKHYCDE) | 119 | 30.25 | 25.32 |
| Acidic (DE) | 47 | 10.82 | 10.00 |
| Basic (KR) | 44 | 11.95 | 9.36 |
| Polar (NCQSTY) | 157 | 34.74 | 33.40 |
| Hydrophobic (AILFWV) | 139 | 28.58 | 29.57 |
| A Ala | 24 | 3.47 | 5.11 |
| C Cys | 4 | 0.79 | 0.85 |
| D Asp | 18 | 3.89 | 3.83 |
| E Glu | 29 | 6.93 | 6.17 |
| F Phe | 14 | 3.76 | 2.98 |
| G Gly | 41 | 5.00 | 8.72 |
| H His | 9 | 2.27 | 1.91 |
| I Ile | 30 | 6.39 | 6.38 |
| K Lys | 11 | 2.61 | 2.34 |
| L Leu | 32 | 6.82 | 6.81 |
| M Met | 8 | 1.94 | 1.70 |
| N Asn | 31 | 6.65 | 6.60 |
| P Pro | 24 | 4.49 | 5.11 |
| Q Gln | 63 | 14.96 | 13.40 |
| R Arg | 33 | 9.34 | 7.02 |
| S Ser | 26 | 4.44 | 5.53 |
| T Thr | 18 | 3.48 | 3.83 |
| V Val | 34 | 6.47 | 7.23 |
| W Trp | 5 | 1.66 | 1.06 |
| Y Tyr | 15 | 4.41 | 3.19 |

Csa17g006960_CruD-1-G1 (mature)

| **Analysis** | **Entire Protein** |
| --- | --- |
| Length | 432 aa |
| Molecular Weight | 47805.38 |
| 1 microgram = | 20.918 pMoles |
| Molar Extinction coefficient | 53110 |
| 1 A[280] corr. to | 0.90 mg/ml |
| A[280] of 1 mg/ml | 1.11 AU |
| Isoelectric Point | 4.98 |
| Charge at pH 7 | -13.67 |

| **Amino Acid(s)** | **Number count** | **% by weight** | **% by frequency** |
| --- | --- | --- | --- |
| Charged (RKHYCDE) | 104 | 28.72 | 24.07 |
| Acidic (DE) | 49 | 12.44 | 11.34 |
| Basic (KR) | 35 | 10.47 | 8.10 |
| Polar (NCQSTY) | 129 | 30.46 | 29.86 |
| Hydrophobic (AILFWV) | 148 | 33.57 | 34.26 |
| A Ala | 35 | 5.61 | 8.10 |
| C Cys | 4 | 0.87 | 0.93 |
| D Asp | 21 | 5.03 | 4.86 |
| E Glu | 28 | 7.41 | 6.48 |
| F Phe | 20 | 5.95 | 4.63 |
| G Gly | 36 | 4.86 | 8.33 |
| H His | 6 | 1.68 | 1.39 |
| I Ile | 22 | 5.19 | 5.09 |
| K Lys | 10 | 2.63 | 2.31 |
| L Leu | 30 | 7.08 | 6.94 |
| M Met | 8 | 2.15 | 1.85 |
| N Asn | 26 | 6.18 | 6.02 |
| P Pro | 20 | 4.14 | 4.63 |
| Q Gln | 37 | 9.73 | 8.56 |
| R Arg | 25 | 7.84 | 5.79 |
| S Ser | 29 | 5.48 | 6.71 |
| T Thr | 23 | 4.93 | 5.32 |
| V Val | 34 | 7.17 | 7.87 |
| W Trp | 7 | 2.57 | 1.62 |
| Y Tyr | 10 | 3.26 | 2.31 |

Csa14g004970_CruD-1-G2 (mature)

| **Analysis** | **Entire Protein** |
| --- | --- |
| Length | 429 aa |
| Molecular Weight | 47472.03 |
| 1 microgram = | 21.065 pMoles |
| Molar Extinction coefficient | 53110 |
| 1 A[280] corr. to | 0.89 mg/ml |
| A[280] of 1 mg/ml | 1.12 AU |
| Isoelectric Point | 5.09 |
| Charge at pH 7 | -11.68 |

| **Amino Acid(s)** | **Number count** | **% by weight** | **% by frequency** |
| --- | --- | --- | --- |
| Charged (RKHYCDE) | 102 | 28.41 | 23.78 |
| Acidic (DE) | 47 | 12.02 | 10.96 |
| Basic (KR) | 35 | 10.54 | 8.16 |
| Polar (NCQSTY) | 130 | 30.89 | 30.30 |
| Hydrophobic (AILFWV) | 144 | 32.86 | 33.57 |
| A Ala | 35 | 5.65 | 8.16 |
| C Cys | 4 | 0.88 | 0.93 |
| D Asp | 20 | 4.82 | 4.66 |
| E Glu | 27 | 7.20 | 6.29 |
| F Phe | 20 | 5.99 | 4.66 |
| G Gly | 36 | 4.90 | 8.39 |
| H His | 6 | 1.69 | 1.40 |
| I Ile | 19 | 4.52 | 4.43 |
| K Lys | 10 | 2.65 | 2.33 |
| L Leu | 29 | 6.89 | 6.76 |
| M Met | 10 | 2.70 | 2.33 |
| N Asn | 26 | 6.22 | 6.06 |
| P Pro | 20 | 4.17 | 4.66 |
| Q Gln | 37 | 9.80 | 8.62 |
| R Arg | 25 | 7.89 | 5.83 |
| S Ser | 29 | 5.52 | 6.76 |
| T Thr | 24 | 5.18 | 5.59 |
| V Val | 34 | 7.22 | 7.93 |
| W Trp | 7 | 2.59 | 1.63 |
| Y Tyr | 10 | 3.28 | 2.33 |

Csa03g005060_CruD-1-G3 (mature)

| **Analysis** | **Entire Protein** |
| --- | --- |
| Length | 429 aa |
| Molecular Weight | 47390.99 |
| 1 microgram = | 21.101 pMoles |
| Molar Extinction coefficient | 53110 |
| 1 A[280] corr. to | 0.89 mg/ml |
| A[280] of 1 mg/ml | 1.12 AU |
| Isoelectric Point | 4.97 |
| Charge at pH 7 | -13.68 |

| **Amino Acid(s)** | **Number count** | **% by weight** | **% by frequency** |
| --- | --- | --- | --- |
| Charged (RKHYCDE) | 100 | 27.84 | 23.31 |
| Acidic (DE) | 47 | 12.07 | 10.96 |
| Basic (KR) | 33 | 9.92 | 7.69 |
| Polar (NCQSTY) | 130 | 30.98 | 30.30 |
| Hydrophobic (AILFWV) | 148 | 33.86 | 34.50 |
| A Ala | 35 | 5.66 | 8.16 |
| C Cys | 4 | 0.88 | 0.93 |
| D Asp | 19 | 4.59 | 4.43 |
| E Glu | 28 | 7.48 | 6.53 |
| F Phe | 20 | 6.00 | 4.66 |
| G Gly | 36 | 4.90 | 8.39 |
| H His | 6 | 1.69 | 1.40 |
| I Ile | 22 | 5.24 | 5.13 |
| K Lys | 10 | 2.65 | 2.33 |
| L Leu | 30 | 7.14 | 6.99 |
| M Met | 8 | 2.17 | 1.86 |
| N Asn | 26 | 6.23 | 6.06 |
| P Pro | 20 | 4.18 | 4.66 |
| Q Gln | 38 | 10.08 | 8.86 |
| R Arg | 23 | 7.27 | 5.36 |
| S Ser | 29 | 5.53 | 6.76 |
| T Thr | 23 | 4.97 | 5.36 |
| V Val | 34 | 7.23 | 7.93 |
| W Trp | 7 | 2.59 | 1.63 |
| Y Tyr | 10 | 3.29 | 2.33 |

Csa11g017000.1a _CsNap-1-G1 (mature)

| **Analysis** | **Entire Protein** |
| --- | --- |
| Length | 115 aa |
| Molecular Weight | 13189.52 |
| 1 microgram = | 75.818 pMoles |
| Molar Extinction coefficient | 7930 |
| 1 A[280] corr. to | 1.66 mg/ml |
| A[280] of 1 mg/ml | 0.60 AU |
| Isoelectric Point | 9.49 |
| Charge at pH 7 | 7.87 |

| **Amino Acid(s)** | **Number count** | **% by weight** | **% by frequency** |
| --- | --- | --- | --- |
| Charged (RKHYCDE) | 33 | 32.65 | 28.70 |
| Acidic (DE) | 7 | 6.48 | 6.09 |
| Basic (KR) | 15 | 16.59 | 13.04 |
| Polar (NCQSTY) | 44 | 39.22 | 38.26 |
| Hydrophobic (AILFWV) | 29 | 23.44 | 25.22 |
| A Ala | 8 | 4.68 | 6.96 |
| C Cys | 8 | 6.36 | 6.96 |
| D Asp | 3 | 2.62 | 2.61 |
| E Glu | 4 | 3.86 | 3.48 |
| F Phe | 3 | 3.25 | 2.61 |
| G Gly | 6 | 2.95 | 5.22 |
| H His | 2 | 2.04 | 1.74 |
| I Ile | 3 | 2.58 | 2.61 |
| K Lys | 3 | 2.88 | 2.61 |
| L Leu | 9 | 7.74 | 7.83 |
| M Met | 1 | 0.98 | 0.87 |
| N Asn | 3 | 2.60 | 2.61 |
| P Pro | 11 | 8.31 | 9.57 |
| Q Gln | 25 | 23.97 | 21.74 |
| R Arg | 12 | 13.71 | 10.43 |
| S Ser | 4 | 2.76 | 3.48 |
| T Thr | 3 | 2.34 | 2.61 |
| V Val | 5 | 3.84 | 4.35 |
| W Trp | 1 | 1.34 | 0.87 |
| Y Tyr | 1 | 1.19 | 0.87 |

Csa11g017000b_CsNap-2-G1 (mature)

| **Analysis** | **Entire Protein** |
| --- | --- |
| Length | 115 aa |
| Molecular Weight | 13176.53 |
| 1 microgram = | 75.893 pMoles |
| Molar Extinction coefficient | 2240 |
| 1 A[280] corr. to | 5.88 mg/ml |
| A[280] of 1 mg/ml | 0.17 AU |
| Isoelectric Point | 9.76 |
| Charge at pH 7 | 8.86 |

| **Amino Acid(s)** | **Number count** | **% by weight** | **% by frequency** |
| --- | --- | --- | --- |
| Charged (RKHYCDE) | 32 | 31.90 | 27.83 |
| Acidic (DE) | 6 | 5.52 | 5.22 |
| Basic (KR) | 15 | 16.79 | 13.04 |
| Polar (NCQSTY) | 47 | 41.77 | 40.87 |
| Hydrophobic (AILFWV) | 24 | 19.18 | 20.87 |
| A Ala | 5 | 2.92 | 4.35 |
| C Cys | 8 | 6.36 | 6.96 |
| D Asp | 3 | 2.62 | 2.61 |
| E Glu | 3 | 2.90 | 2.61 |
| F Phe | 2 | 2.17 | 1.74 |
| G Gly | 7 | 3.45 | 6.09 |
| H His | 2 | 2.04 | 1.74 |
| I Ile | 4 | 3.45 | 3.48 |
| K Lys | 2 | 1.92 | 1.74 |
| L Leu | 7 | 6.03 | 6.09 |
| M Met | 3 | 2.94 | 2.61 |
| N Asn | 3 | 2.60 | 2.61 |
| P Pro | 11 | 8.31 | 9.57 |
| Q Gln | 27 | 25.91 | 23.48 |
| R Arg | 13 | 14.87 | 11.30 |
| S Ser | 6 | 4.14 | 5.22 |
| T Thr | 2 | 1.56 | 1.74 |
| V Val | 6 | 4.61 | 5.22 |
| W Trp | 0 | 0.00 | 0.00 |
| Y Tyr | 1 | 1.19 | 0.87 |

Csa11g017010_CsNap-3-G1 (mature)

| **Analysis** | **Entire Protein** |
| --- | --- |
| Length | 112 aa |
| Molecular Weight | 12779.19 |
| 1 microgram = | 78.252 pMoles |
| Molar Extinction coefficient | 2240 |
| 1 A[280] corr. to | 5.70 mg/ml |
| A[280] of 1 mg/ml | 0.18 AU |
| Isoelectric Point | 9.54 |
| Charge at pH 7 | 7.95 |

| **Amino Acid(s)** | **Number count** | **% by weight** | **% by frequency** |
| --- | --- | --- | --- |
| Charged (RKHYCDE) | 30 | 30.66 | 26.79 |
| Acidic (DE) | 5 | 4.79 | 4.46 |
| Basic (KR) | 13 | 14.94 | 11.61 |
| Polar (NCQSTY) | 43 | 39.18 | 38.39 |
| Hydrophobic (AILFWV) | 26 | 22.00 | 23.21 |
| A Ala | 5 | 3.01 | 4.46 |
| C Cys | 8 | 6.56 | 7.14 |
| D Asp | 2 | 1.80 | 1.79 |
| E Glu | 3 | 2.99 | 2.68 |
| F Phe | 4 | 4.47 | 3.57 |
| G Gly | 7 | 3.56 | 6.25 |
| H His | 3 | 3.15 | 2.68 |
| I Ile | 4 | 3.55 | 3.57 |
| K Lys | 2 | 1.98 | 1.79 |
| L Leu | 7 | 6.21 | 6.25 |
| M Met | 3 | 3.03 | 2.68 |
| N Asn | 3 | 2.68 | 2.68 |
| P Pro | 12 | 9.35 | 10.71 |
| Q Gln | 23 | 22.74 | 20.54 |
| R Arg | 11 | 12.97 | 9.82 |
| S Ser | 5 | 3.56 | 4.46 |
| T Thr | 3 | 2.42 | 2.68 |
| V Val | 6 | 4.76 | 5.36 |
| W Trp | 0 | 0.00 | 0.00 |
| Y Tyr | 1 | 1.23 | 0.89 |

Csa11g017020_CsNap-4-G1 (mature)

| **Analysis** | **Entire Protein** |
| --- | --- |
| Length | 116 aa |
| Molecular Weight | 13225.44 |
| 1 microgram = | 75.612 pMoles |
| Molar Extinction coefficient | 2240 |
| 1 A[280] corr. to | 5.90 mg/ml |
| A[280] of 1 mg/ml | 0.17 AU |
| Isoelectric Point | 9.87 |
| Charge at pH 7 | 8.86 |

| **Amino Acid(s)** | **Number count** | **% by weight** | **% by frequency** |
| --- | --- | --- | --- |
| Charged (RKHYCDE) | 32 | 31.94 | 27.59 |
| Acidic (DE) | 6 | 5.50 | 5.17 |
| Basic (KR) | 15 | 16.90 | 12.93 |
| Polar (NCQSTY) | 48 | 42.27 | 41.38 |
| Hydrophobic (AILFWV) | 25 | 19.90 | 21.55 |
| A Ala | 6 | 3.49 | 5.17 |
| C Cys | 8 | 6.34 | 6.90 |
| D Asp | 3 | 2.61 | 2.59 |
| E Glu | 3 | 2.89 | 2.59 |
| F Phe | 3 | 3.24 | 2.59 |
| G Gly | 8 | 3.93 | 6.90 |
| H His | 2 | 2.03 | 1.72 |
| I Ile | 4 | 3.43 | 3.45 |
| K Lys | 1 | 0.96 | 0.86 |
| L Leu | 6 | 5.15 | 5.17 |
| M Met | 2 | 1.95 | 1.72 |
| N Asn | 3 | 2.59 | 2.59 |
| P Pro | 10 | 7.53 | 8.62 |
| Q Gln | 27 | 25.79 | 23.28 |
| R Arg | 14 | 15.94 | 12.07 |
| S Ser | 7 | 4.81 | 6.03 |
| T Thr | 2 | 1.56 | 1.72 |
| V Val | 6 | 4.59 | 5.17 |
| W Trp | 0 | 0.00 | 0.00 |
| Y Tyr | 1 | 1.18 | 0.86 |

Csa12g024720a_CsNap-1-G3 (mature)

| **Analysis** | **Entire Protein** |
| --- | --- |
| Length | 115 aa |
| Molecular Weight | 13195.47 |
| 1 microgram = | 75.784 pMoles |
| Molar Extinction coefficient | 7930 |
| 1 A[280] corr. to | 1.66 mg/ml |
| A[280] of 1 mg/ml | 0.60 AU |
| Isoelectric Point | 9.69 |
| Charge at pH 7 | 8.78 |

| **Amino Acid(s)** | **Number count** | **% by weight** | **% by frequency** |
| --- | --- | --- | --- |
| Charged (RKHYCDE) | 33 | 32.67 | 28.70 |
| Acidic (DE) | 7 | 6.39 | 6.09 |
| Basic (KR) | 16 | 17.73 | 13.91 |
| Polar (NCQSTY) | 45 | 40.07 | 39.13 |
| Hydrophobic (AILFWV) | 28 | 22.57 | 24.35 |
| A Ala | 8 | 4.67 | 6.96 |
| C Cys | 8 | 6.36 | 6.96 |
| D Asp | 4 | 3.49 | 3.48 |
| E Glu | 3 | 2.89 | 2.61 |
| F Phe | 3 | 3.25 | 2.61 |
| G Gly | 6 | 2.95 | 5.22 |
| H His | 1 | 1.02 | 0.87 |
| I Ile | 3 | 2.58 | 2.61 |
| K Lys | 3 | 2.88 | 2.61 |
| L Leu | 8 | 6.88 | 6.96 |
| M Met | 1 | 0.98 | 0.87 |
| N Asn | 3 | 2.60 | 2.61 |
| P Pro | 11 | 8.30 | 9.57 |
| Q Gln | 26 | 24.92 | 22.61 |
| R Arg | 13 | 14.85 | 11.30 |
| S Ser | 5 | 3.45 | 4.35 |
| T Thr | 2 | 1.56 | 1.74 |
| V Val | 5 | 3.84 | 4.35 |
| W Trp | 1 | 1.34 | 0.87 |
| Y Tyr | 1 | 1.19 | 0.87 |

Csa12g024720b_CsNap-2-G3 (mature)

| **Analysis** | **Entire Protein** |
| --- | --- |
| Length | 116 aa |
| Molecular Weight | 13280.53 |
| 1 microgram = | 75.298 pMoles |
| Molar Extinction coefficient | 2240 |
| 1 A[280] corr. to | 5.93 mg/ml |
| A[280] of 1 mg/ml | 0.17 AU |
| Isoelectric Point | 9.60 |
| Charge at pH 7 | 7.87 |

| **Amino Acid(s)** | **Number count** | **% by weight** | **% by frequency** |
| --- | --- | --- | --- |
| Charged (RKHYCDE) | 35 | 34.88 | 30.17 |
| Acidic (DE) | 8 | 7.39 | 6.90 |
| Basic (KR) | 16 | 17.97 | 13.79 |
| Polar (NCQSTY) | 45 | 39.62 | 38.79 |
| Hydrophobic (AILFWV) | 24 | 18.66 | 20.69 |
| A Ala | 6 | 3.48 | 5.17 |
| C Cys | 8 | 6.31 | 6.90 |
| D Asp | 3 | 2.60 | 2.59 |
| E Glu | 5 | 4.79 | 4.31 |
| F Phe | 2 | 2.15 | 1.72 |
| G Gly | 8 | 3.91 | 6.90 |
| H His | 2 | 2.02 | 1.72 |
| I Ile | 3 | 2.56 | 2.59 |
| K Lys | 1 | 0.95 | 0.86 |
| L Leu | 6 | 5.13 | 5.17 |
| M Met | 3 | 2.92 | 2.59 |
| N Asn | 2 | 1.72 | 1.72 |
| P Pro | 10 | 7.50 | 8.62 |
| Q Gln | 26 | 24.75 | 22.41 |
| R Arg | 15 | 17.02 | 12.93 |
| S Ser | 6 | 4.11 | 5.17 |
| T Thr | 2 | 1.55 | 1.72 |
| V Val | 7 | 5.34 | 6.03 |
| W Trp | 0 | 0.00 | 0.00 |
| Y Tyr | 1 | 1.18 | 0.86 |

Csa12g024730a_CsNap-3-G3 (mature)

| **Analysis** | **Entire Protein** |
| --- | --- |
| Length | 117 aa |
| Molecular Weight | 13322.51 |
| 1 microgram = | 75.061 pMoles |
| Molar Extinction coefficient | 2240 |
| 1 A[280] corr. to | 5.95 mg/ml |
| A[280] of 1 mg/ml | 0.17 AU |
| Isoelectric Point | 9.60 |
| Charge at pH 7 | 7.87 |

| **Amino Acid(s)** | **Number count** | **% by weight** | **% by frequency** |
| --- | --- | --- | --- |
| Charged (RKHYCDE) | 35 | 34.74 | 29.91 |
| Acidic (DE) | 8 | 7.36 | 6.84 |
| Basic (KR) | 16 | 17.90 | 13.68 |
| Polar (NCQSTY) | 46 | 40.42 | 39.32 |
| Hydrophobic (AILFWV) | 25 | 19.26 | 21.37 |
| A Ala | 7 | 4.05 | 5.98 |
| C Cys | 8 | 6.29 | 6.84 |
| D Asp | 3 | 2.59 | 2.56 |
| E Glu | 5 | 4.77 | 4.27 |
| F Phe | 2 | 2.14 | 1.71 |
| G Gly | 9 | 4.38 | 7.69 |
| H His | 2 | 2.01 | 1.71 |
| I Ile | 4 | 3.40 | 3.42 |
| K Lys | 1 | 0.95 | 0.85 |
| L Leu | 6 | 5.11 | 5.13 |
| M Met | 2 | 1.94 | 1.71 |
| N Asn | 2 | 1.71 | 1.71 |
| P Pro | 9 | 6.72 | 7.69 |
| Q Gln | 27 | 25.60 | 23.08 |
| R Arg | 15 | 16.95 | 12.82 |
| S Ser | 6 | 4.09 | 5.13 |
| T Thr | 2 | 1.55 | 1.71 |
| V Val | 6 | 4.56 | 5.13 |
| W Trp | 0 | 0.00 | 0.00 |
| Y Tyr | 1 | 1.17 | 0.85 |

Csa12g024730b_CsNap-4-G3 (mature)

| **Analysis** | **Entire Protein** |
| --- | --- |
| Length | 113 aa |
| Molecular Weight | 12945.36 |
| 1 microgram = | 77.248 pMoles |
| Molar Extinction coefficient | 2240 |
| 1 A[280] corr. to | 5.78 mg/ml |
| A[280] of 1 mg/ml | 0.17 AU |
| Isoelectric Point | 9.76 |
| Charge at pH 7 | 8.95 |

| **Amino Acid(s)** | **Number count** | **% by weight** | **% by frequency** |
| --- | --- | --- | --- |
| Charged (RKHYCDE) | 33 | 33.50 | 29.20 |
| Acidic (DE) | 6 | 5.62 | 5.31 |
| Basic (KR) | 15 | 17.09 | 13.27 |
| Polar (NCQSTY) | 42 | 37.82 | 37.17 |
| Hydrophobic (AILFWV) | 25 | 20.63 | 22.12 |
| A Ala | 5 | 2.98 | 4.42 |
| C Cys | 8 | 6.48 | 7.08 |
| D Asp | 3 | 2.67 | 2.65 |
| E Glu | 3 | 2.95 | 2.65 |
| F Phe | 3 | 3.31 | 2.65 |
| G Gly | 7 | 3.51 | 6.19 |
| H His | 3 | 3.11 | 2.65 |
| I Ile | 4 | 3.51 | 3.54 |
| K Lys | 2 | 1.95 | 1.77 |
| L Leu | 7 | 6.14 | 6.19 |
| M Met | 3 | 2.99 | 2.65 |
| N Asn | 2 | 1.77 | 1.77 |
| P Pro | 12 | 9.23 | 10.62 |
| Q Gln | 23 | 22.46 | 20.35 |
| R Arg | 13 | 15.13 | 11.50 |
| S Ser | 5 | 3.51 | 4.42 |
| T Thr | 3 | 2.39 | 2.65 |
| V Val | 6 | 4.70 | 5.31 |
| W Trp | 0 | 0.00 | 0.00 |
| Y Tyr | 1 | 1.21 | 0.88 |
