# Supplementary material for: Genetic variation and structural diversity in major seed proteins among and within Camelina species
Source: Planta. 2022 Oct 6;256(5):93. doi: 10.1007/s00425-022-03998-w (PMC9537204; doi:10.1007/s00425-022-03998-w)
Supplement: Supplementary file 4 — Supplementary file4 (DOCX 7072 KB) [file 425_2022_3998_MOESM4_ESM.docx]

**Supplemental Table S3. Seed protein profiles from *C. sativa* accessions.**

| Accession | Reduced  Profile  Number | Non-Reduced  Profile  Number | Reduced digital gel | Reduced protein trace (10-75 kDa) | Non-reduced digital gel | Non-reduced protein trace (10-75 kDa) |
| --- | --- | --- | --- | --- | --- | --- |
| CN 101980 | 4 | 1 |   |  |   |  |
| CN 101981 | 1 | 1 |  |  |   |  |
| CN 101982 | 4 | 2 |  |  |  |  |
| CN 101983 | 1 | 1 |  |  |  |  |
| CN 101984 | 1 | 2 ? |  |  |  |  |
| CN 101985 | 4 | 1 |  |  |  |  |
| CN 101986 | 4 | 2 |  |  |  |  |
| CN 101987 | 4 | 1 |  |  |  |  |
| CN 101988 | 4 | 2 |  |  |  |  |
| CN 101989 | 4 | 2 ? |  |  |  |  |
| CN 101990 | 4 | 1 |  |  |  |  |
| CN 111330 | 5 | 2 |  |  |  |  |
| CN 111331 | 7 | 1 |  |  |  |  |
| CN 111332 | 2 | 1 |  |  |  |  |
| CN 111333 | 2 | 1 |  |  |  |  |
| CN 111334 | 2 | 1 |  |  |  |  |
| CN 111335 | 2 | 1 |  |  |  |  |
| CN 111336 | 2 | 1 |  |  |  |  |
| CN 113651 | 4 | 2 |  |  |  |  |
| CN 113652 | 6 | 1 |  |  |  |  |
| CN 113653 | 4 | 1 |  |  |  |  |
| CN 113654 | 4 | 1 |  |  |  |  |
| CN 113655 | 6 | 3 |  |  |  |  |
| CN 113656 | 1 | 1 |  |  |  |  |
| CN 113657 | ** | ? |  |  |  |  |
| CN 113658 | 1 | 1 |  |  |  |  |
| CN 113659 | 4 | 2 |  |  |  |  |
| CN 113660 | 4b | 1 |  |  |  |  |
| CN 113661 | 4 | 3 |  |  |  |  |
| CN 113662 | 4 | 2 |  |  |  |  |
| CN 113663 | 4b | 2 |  |  |  |  |
| CN 113664 | 6 | 3 |  |  |  |  |
| CN 113665 | 6 | 2 |  |  |  |  |
| CN 113666 | 4 | 1 |  |  |  |  |
| CN 113667 | 4 | 1 |  |  |  |  |
| CN 113668 | 4 | 1 |  |  |  |  |
| CN 113669 | 1 | 1 |  |  |  |  |
| CN 113670 | 4 | 1 |  |  |  |  |
| CN 113671 | 6 | 3 |  |  |  |  |
| CN 113672 | 4 | 1 |  |  |  |  |
| CN 113673 | 4 | 1 |  |  |  |  |
| CN 113674 | 4 | 2 |  |  |  |  |
| CN 113675 | 4 | 2 |  |  |  |  |
| CN 113676 | 1 | 1 |  |  |  |  |
| CN 113677 | 6 | 3 |  |  |  |  |
| CN 113678 | 4 | 1 |  |  |  |  |
| CN 113679 | 4 | 1 |  |  |  |  |
| CN 113680 | 4 | 1 |  |  |  |  |
| CN 113681 | 4 | 2 |  |  |  |  |
| CN 113682 | 4 | 1 |  |  |  |  |
| CN 113683 | 4 | 2 |  |  |  |  |
| CN 113684 | ** | 2 |  |  |  |  |
| CN 113685 | 4 | 1 |  |  |  |  |
| CN 113686 | 4 | 1 |  |  |  |  |
| CN 113687 | ** | 1 |  |  |  |  |
| CN 113688 | 4 | 2 |  |  |  |  |
| CN 113689 | 6 | 3 |  |  |  |  |
| CN 113690 | 6 | 3 |  |  |  |  |
| CN 113691 | 4 | 1 |  |  |  |  |
| CN 113692 | 5 | 2 |  |  |  |  |
| CN 113693 | 4 | 2 |  |  |  |  |
| CN 113694 | 5 | 2 |  |  |  |  |
| CN 113695 | 4 | 2 |  |  |  |  |
| CN 113696 | 1 | 1 |  |  |  |  |
| CN 113697 | 1 | 1 |  |  |  |  |
| CN 113698 | 1 | 1 |  |  |  |  |
| CN 113699 | 4 | 1 |  |  |  |  |
| CN 113700 | 1 | 1 |  |  |  |  |
| CN 113701 | 1 | 1 |  |  |  |  |
| CN 113702 | 4 | 2 |  |  |  |  |
| CN 113703 | 5 | 1 |  |  |  |  |
| CN 113704 | 4 | 1 |  |  |  |  |
| CN 113705 | 6 | 1 |  |  |  |  |
| CN 113706 | 5 | 1 |  |  |  |  |
| CN 113707 | 4 | 1 |  |  |  |  |
| CN 113708 | 5 | 1 |  |  |  |  |
| CN 113709 | 1 | 3 |  |  |  |  |
| CN 113710 | 4 | 1 |  |  |  |  |
| CN 113711 | 4 ? | 1 |  |  |  |  |
| CN 113712 | 1 | 1 |  |  |  |  |
| CN 113713 | 1 | 1 |  |  |  |  |
| CN 113714 | 1 | 1 |  |  |  |  |
| CN 113715 | 4 | 1 |  |  |  |  |
| CN 113716 | 1 | 1 |  |  |  |  |
| CN 113717 | 1 | 3 |  |  |  |  |
| CN 113718 | 1 | 3 |  |  |  |  |
| CN 113719 | 1 | 1 ? |  |  |  |  |
| CN 113720 | 4 | 3 |  |  |  |  |
| CN 113721 | 5 | 1 |  |  |  |  |
| CN 113722 | 4 | 1 |  |  |  |  |
| CN 113723 | 1 ? | 1 |  |  |  |  |
| CN 113724 | 4 | 1 |  |  |  |  |
| CN 113725 | 5 | 2 |  |  |  |  |
| CN 113726 | 1 | 1 ? |  |  |  |  |
| CN 113727 | 4 | 1 ? |  |  |  |  |
| CN 113728 | 1 | 1 |  |  |  |  |
| CN 113729 | 4 | 1 |  |  |  |  |
| CN 113730 | 1 | 1 |  |  |  |  |
| CN 113731 | 1 | 1 |  |  |  |  |
| CN 113732 | 1 | 1 |  |  |  |  |
| CN 113733 | 1 | 2 ? |  |  |  |  |
| CN 113734 | 5 | 1 ? |  |  |  |  |
| CN 113735 | 4 | 2 |  |  |  |  |
| CN 113736 | 5 | 1 ? |  |  |  |  |
| CN 113737 | 2 | 1 |  |  |  |  |
| CN 113738 | 1 | 1 |  |  |  |  |
| CN 113739 | 4 | 1 |  |  |  |  |
| CN 113740 | 4 | 1 |  |  |  |  |
| CN 113741 | 4 | 1 |  |  |  |  |
| CN 113742 | 5 | 2 |  |  |  |  |
| CN 113743 | 4 | 1 |  |  |  |  |
| CN 113744 | 4 | 1 |  |  |  |  |
| CN 113745 | 5 | 2 ? |  |  |  |  |
| CN 113746 | 1 | 1 ? |  |  |  |  |
| CN 113747 | 1 | 1 |  |  |  |  |
| CN 113748 | 1 | 1 |  |  |  |  |
| CN 113749 | 5b | 2 |  |  |  |  |
| CN 113750 | 1 | 2 |  |  |  |  |
| CN 113751 | 1 | 1 |  |  |  |  |
| CN 113752 | 1 | 1 |  |  |  |  |
| CN 113753 | 5 | 2 |  |  |  |  |
| CN 113754 | 4 | 2 |  |  |  |  |
| CN 113755 | 4 | 2 |  |  |  |  |
| CN 113756 | 1 | 2 |  |  |  |  |
| CN 113757 | 1 | 2 |  |  |  |  |
| CN 113758 | 1 | 2 |  |  |  |  |
| CN 113759 | 1 | 2 |  |  |  |  |
| CN 113760 | 4 | 1 |  |  |  |  |
| CN 114242 | 4 | 1 |  |  |  |  |
| CN 114243 | 4 | 1 |  |  |  |  |
| CN 114244 | 4 | 2 |  |  |  |  |
| CN 114245 | 1 | 1 |  |  |  |  |
| CN 114246 | 1 | 1 | - |  |  |  |
| CN 114247 | 1 | 1 |  |  |  |  |
| CN 114248 | 2 | 1 ? |  |  |  |  |
| CN 114249 | 4 | 1 |  |  |  |  |
| CN 114250 | 5 | 2 |  |  |  |  |
| CN 114251 | 5b | 2 |  |  |  |  |
| CN 114252 | 5 | 2 |  |  |  |  |
| CN 114253 | 5 | 2 |  |  |  |  |
| CN 114254 | 5 | 1 |  |  |  |  |
| CN 114255 | 5 | 2 |  |  |  |  |
| CN 114256 | 5 | 2 |  |  |  |  |
| CN 114257 | 1 | 1 |  |  |  |  |
| CN 114258 | 1 | 1 |  |  |  |  |
| CN 114259 | 5 | 1 |  |  |  |  |
| CN 114260 | 5b | 2 |  |  |  |  |
| CN 114261 | 5 | 1 |  |  |  |  |
| CN 114262 | 1 | 1 |  |  |  |  |
| CN 114263 | 4 | 1 |  |  |  |  |
| CN 114264 | 7 | 3 |  |  |  |  |
| CN 114265 | 7 | 3 |  |  |  |  |
| CN 114266 | 4 | 1 |  |  |  |  |
| CN 114267 | 1 | 2 |  |  |  |  |
| CN 114268 | 4 | 2 |  |  |  |  |
| CN 114269 | 4b | 1 |  |  |  |  |
| CN 114270 | 5 | 2 |  |  |  |  |
| CN 114271 | 1 | 1 |  |  |  |  |
| CN 114272 | 5 | 2 |  |  |  |  |
| CN 114273 | 4 | 1 |  |  |  |  |
| CN 114274 | 6 | 3 |  |  |  |  |
| CN 114275 | 1 | 1 |  |  |  |  |
| CN 114276 | 5b | 2 |  |  |  |  |
| CN 114277 | 4b | 1 |  |  |  |  |
| CN 114278 | 7 | 1 |  |  |  |  |
| CN 114279 | 2** | ? |  |  |  |  |
| CN 114280 | 2** | ? |  |  |  |  |
| CN 114281 | 4b | 1 |  |  |  |  |
| CN 114282 | 7 | 3 |  |  |  |  |
| CN 114283 | 7 | 3 |  |  |  |  |
| CN 30475 | 1 | 1 |  |  |  |  |
| CN 30476 | 1 | 1 |  |  |  |  |
| CN 30477 | 2 | 1 |  |  |  |  |
| CN 30478 | 1 | 1 |  |  |  |  |
| CN 30479 | 1 | 2 |  |  |  |  |
| CN 45816 | 2 | 1 |  |  |  |  |
| L-009-16 | 1 | 1 ? |  |  |  |  |
| L-010-16 | 1 | 2 |  |  |  |  |
| L-035-16 | 1 | 1 |  |  |  |  |
| L-037-16 | 1 | 1 |  |  |  |  |
| L-046-16 | 6 | 1 |  |  |  |  |
| L-050-16 | 7 | 3 |  |  |  |  |
| L-051-16 | 2 | 1 |  |  |  |  |
| L-051-16 | 1 | 1 |  |  |  |  |
| L-054-16 | 1 | 1 |  |  |  |  |
| L-083-16 | 5 | 2 |  |  |  |  |
| L-114-16 | 2 | 1 |  |  |  |  |
