# Supplementary material for: Genetic variation and structural diversity in major seed proteins among and within Camelina species
Source: Planta. 2022 Oct 6;256(5):93. doi: 10.1007/s00425-022-03998-w (PMC9537204; doi:10.1007/s00425-022-03998-w)
Supplement: Supplementary file 11 — Supplementary file11 (PDF 235 KB) [file 425_2022_3998_MOESM11_ESM.pdf]

Supplemental Fig. S6. Alignment of *C. sativa* and *A. thaliana* cruciferin protomers.

```
                    AtCRA    (1) MA--RVSSLLSFCLTLLILFHGYAAQQGQ-QGQQFPNECQLDQLNALEPSHVLKSEAGRIEVWDHHAPQLRCSGVSFARYIIESKGLYLPSFFNTAKLSF
Csa11g070580.1_CsCruA-1-G1   (1) MA--RVSSLLSFSLTLLIFFHGYTAQQ-------FPNECQLDQLNALEPSHVLKSEAGRIEVWDHHAPQLRCSGVSFARYVIESKGLYLPSFVNTAKLSF
Csa11g070590.1_CsCruA-2-G1   (1) MA--RVSSLLSFSLTLLIFFHGYTAQQ-------FPNECQLDQLNALEPSHVLKSEAGRIEVWDHHAPQLRCSGVSFTRYVIESKGLYLPSFVNTAKLSF
Csa18g009670.1_CsCruA-1-G2   (1) MA--RV---FSFSLTLLIFFHCYTAQQ-------FPNECQLDQLNALEPSHVLKSEAGRIEVWDHHAPQLRCSGVSFTRYVIESKGLYLPSFVNTAKLSF
                    AtCRB    (1) MG--RVSSIISFSLTLLILFNGYTAQQ-------WPNECQLDQLNALEPSQIIKSEGGRIEVWDHHAPQLRCSGFAFERFVIEPQGLFLPTFLNAGKLTF
Csa03g005050.1_CsCruB-1-G3   (1) MG--RTSSIVSFSLTLLILFHGYTAQQ-------WPNECQLDQLNALEPSQVIQSEGGRIEVWDHHAPQLRCSGFAFERFVIEPQGLYLPTFMNAGKLTF
Csa14g004960.1_CsCruB-1-G2   (1) MG--RASSIVSFSLTLLILFHGYTAQQ-------WPNECQLDQLNALEPSQVIQSEGGRIEVWDHHAPQLRCSGFAFERFVIEPQGLYLPTFMNAGKLTF
Csa17g006950.1_CsCruB-1-G1   (1) MG--RASSIVSFSLTLLILFHGYTAQQ-------WPNECQLDQLNALEPSQVIQSEGGRIEVWDHHAPQLRCSGFAFERFVIEPQGLYLPTFMNAGKLTF
                    AtCRC    (1) MV--KLSNLLVATFGVLLVLNGCLARQSLGVPPQLQNECNLDNLDVLQATETIKSEAGQIEYWDHNHPQLRCVGVSVARYVIEQGGLYLPTFFTSPKISY
Csa10g014100.1_CsCruC-1-G2   (1) MV--KLSNLLVATFGVLLVLNGCLARQSLGVPPQLQNECNLDNLDVLEATETIKSEAGQIEYWDHNHPHLRCAGVSVARYVIEQGGLYLPTFFTSPKISY
Csa11g015240.1_CsCruC-1-G1   (1) MG--KLSNLLVATFGVLLVLNGCLARQSLGVPPQLQNECNLDNLDVLEATETIKSEAGQIEYWDHNHPHLRCAGVSVARYVIEQGGLYLPTFFTSPKISY
Csa12g021990.1_CsCruC-1-G3   (1) MV--KLSNLLVATFGVLLVLNGCFARQSLRVPPQLQNECNLDNLDVLEATETIKSEAGQIEYWDHNHPHLRCAGVSVARYVIEQGGLYLPTFFNSPKISY
Csa03g005060.1_CsCruD-1-G3   (1) MHKLLFSLLTVVSLSLLLFFHGGEARQQE---TPFPNACHFSQINSLAPAQATKFEAGQMEVWDHMSPELQCAGVTVVRITLQSNSIFLPAFFSPPALAY
Csa14g004970.1_CsCruD-1-G2   (1) MHKLLFSLLTVVSLSLLLFFHGGEARQQE---TPFPNACHFSQINSLAPAQATKFEAGQMEVWDHMSPELQCAGVTVVRITLQSNSIFLPAFFSPPALAY
Csa17g006960.1_CsCruD-1-G1   (1) MHKLLFSLLIVVSLSLLLFFHGGEARQQE---TPFPNACHFSQINSLAPAQATKFEAGQMEVWDHMSPELQCAGVTVVRITLQSNSIFLPAFFSPPALAY
                                 sssssssssssssssssssssssss???????????
                                                         HVR-I

                    AtCRA   (98) VAKGRGLMGKVIPGCAETFQDSSEFQPR--------------------------------FEGQG---QSQ------------------RFRDMHQKVEH
Csa11g070580.1_CsCruA-1-G1  (92) VAQGRGLMGKVIPGCAETFQDSSVFQPRQGRQ----------------------------FEGQGEEGQSQ------------------RFHDMHQKVEH
Csa11g070590.1_CsCruA-2-G1  (92) VAQGRGLMGKVIPGCAETFQDSSVFQPRQGRQ----------------------------FEGQGEEGQSQ------------------RFHDMHQKVEH
Csa18g009670.1_CsCruA-1-G2  (89) VAQGRGLMGKVIPGCAETFQDSSVFQPRQGRQ----------------------------FEGQGEEGQSQ------------------KFHDMHQKVEH
                    AtCRB   (92) VVHGRGLMGRVIPGCAETFMESPVFGEG---------------------------------QGQG-QSQG--------------------FRDMHQKVEH
Csa03g005050.1_CsCruB-1-G3  (92) VVHGTGLMGRVIPGCAETFIESPVFGES---------------------------------QGQG-QSQG--------------------FRDMHQKVEH
Csa14g004960.1_CsCruB-1-G2  (92) VVHGTGLMGRVVPGCAETFIESPVFGES---------------------------------QGQS-QGQG--------------------FRDMHQKVEH
Csa17g006950.1_CsCruB-1-G1  (92) VVHGTGLMGRVVPGCPETFIESPVFGES---------------------------------QGQG-QGQGQ------------------GFRDMHQKVEH
                    AtCRC   (99) VVQGTGISGRVVPGCAETFMDSQPMQGQQQGQPWQGRQGQQGQPWEGQGQQGQQGRQGQPWEGQGQQGQQGRQGQQGQPWEGQGQQGQQGFRDMHQKVEH
Csa10g014100.1_CsCruC-1-G2  (99) VVQGRGISGRVVPGCAETFMDSQPMQGEQQGQ--QGR------------------RKEQPWQGQGQQGQ----GQQGQ-------QQQQGFRDMHQKVEH
Csa11g015240.1_CsCruC-1-G1  (99) VVQGKGISGRVVPGCAETFMDSQPMQGEQQGQ--QGR------------------RQEQPWQGQGQQGQ----GQQGQ-------QQQQGFRDMHQKVEH
Csa12g021990.1_CsCruC-1-G3  (99) VVQGRGISGRVVPGCAETFMDSQPMQGEQQGQ--QGR------------------RQQQPWQGQGQQGE----GQQGQ-------QQQQGFRDMHQKVEH
Csa03g005060.1_CsCruD-1-G3  (98) VVQGEGVIGTIASGCPETYEDIEASGRGG---------------------------------GGGDRQRR--------------------FEDMHQKLEN
Csa14g004970.1_CsCruD-1-G2  (98) VVQGEGVMGTIASGCPETYEDIEASGRGG---------------------------------GGGDRRRR--------------------FEDMHQKLEN
Csa17g006960.1_CsCruD-1-G1  (98) VVQGEGVMGTIASGCPETYEDIEASDRGGE-------------------------------GGGGDRRRR--------------------FEDMHQKLEN
                                                   ????????????????????????????????????????????????????????????????????????
                                                   HVR-II

                    AtCRA  (145) IRSGDTIATTPGVAQWFYNDGQEPLVIVSVFDLASHQNQLDRNPRPFYLAGNNPQGQVWLQGREQQPQKNIFNGFGPEVIAQALKIDLQTAQQLQNQDDN
Csa11g070580.1_CsCruA-1-G1 (146) IRSGDTIATTPGVAQWFYNDGHQPLVIVSVFDLASHQNQLDRNPRPFYLAGNNPQGQVWLHGREQQPQKNIFSGFGPEVIAQALKIDLQTAQQLQNQDDN
Csa11g070590.1_CsCruA-2-G1 (146) IRSGDTIATTPGVAQWFYNDGQQPLVIVSVFDLASHQNQLDRNPRPFYLAGNNPQGQVWLHGREQQPQKNIFSGFGPEVIAQALKIDLQTAQQLQNQDDN
Csa18g009670.1_CsCruA-1-G2 (143) IRSGDTIATTPGVAQWFYNDGQQPLVIVSVFDLASHQNQLDRNPRPFYLAGNNPQGQVWLHEREQQPQKNIFSGFGPEVIAQALKIDLQTAQQLQNQDDN
                    AtCRB  (138) LRCGDTIATPSGVAQWFYNNGNEPLILVAAADLASNQNQLDRNLRPFLIAGNNPQGQEWLQGRKQQKQNNIFNGFAPEILAQAFKINVETAQQLQNQQDN
Csa03g005050.1_CsCruB-1-G3 (138) LRCGDTIATPSGVAQWFYNNGNEPLILVAAADLANNQNQLDRNLRPVLIAGNNPQGQEWLQGRQQQKQNNIFTGFAPEILAQAFKINVETAQQLQSHQDN
Csa14g004960.1_CsCruB-1-G2 (138) LRCGDTIATPSGVAQWFYNNGNEPLILVAAADLANNQNQLDRNLRPVLIAGNNPQGQEWLQGRQQQKQNNIFTGFAPEILAQAFKINVETAQQLQSHQDN
Csa17g006950.1_CsCruB-1-G1 (140) LRCGDTIATPSGVAQWFYNNGNEPLILVAAADLANHENQLDRNLRPVLIAGNNPQGQEWLQGRQQQKQNNIFTGFAPEILAQAFKINVETAQQLQSHQDN
                    AtCRC  (199) VRRGDVFANTPGSAHWIYNSGEQPLVIIALLDIANYQNQLDRNPRVFHLAGNNQQGG-FGGSQQQQEQKNLWSGFDAQVIAQALKIDVQLAQQLQNQQDS
Csa10g014100.1_CsCruC-1-G2 (168) VRQGDVFAITPGSAHWIYNSGEQPLVIIALLDIANYQNQLDRNPRVFRLAGNNQQGG-FGGSQQQQEQKNMWSGFDAQIIAQALKIDVQLAQELQNQQDS
Csa11g015240.1_CsCruC-1-G1 (168) VRQGDVFAITPGSAHWIYNSGEQPLVIIALLDIANYQNQLDRNPRVFRLAGNNQQGG-FGGSQQQQEQKNMWSGFDAQIIAQALKIDVQLAQELQNQQDS
Csa12g021990.1_CsCruC-1-G3 (168) VRQGDVFAITPGSAHWIYNTGEQPLVIIALIDIANYQNQLDRNPRVFHLAGNNQQGG-FGGSQQQQEQKNMWSSFDAQIIAQALKIDVQLAQELQNQQDS
Csa03g005060.1_CsCruD-1-G3 (145) FRRGDVFALLAGVSQWWYNSGDSDVVIVIVLDVTNRENQLDQVPRMFQLAGSRTQEEEQQQPLNWPSGNNAFSGFDANIIAEAFKIDIETAKQLQNQQDN
Csa14g004970.1_CsCruD-1-G2 (145) FRRGDVFASLAGVSQWWYNRGDSDVVIVIVLDVTNRENQLDQVPRMFQLAGSRTQEEEQQQPLNWPSGNNAFSGFDANIIAEAFKVDTETAKQLQNQQDN
Csa17g006960.1_CsCruD-1-G1 (147) FRRGDVFASLAGVSQWWYNRGDSDVVIVIVLDVTNRENQLDQVPRMFQLAGSRTQEEEQQQPLNWPSGNNAFSGFDANIIAEAFKIDIETAKQLQNQQDN
                                                                                        ??????????????
                                                                                        HVR-III
```

```
                     AtCRA  (245) RGNIVRVQGPFGVIRPPLR-GQRPQEEEEEEGRHGRHGNGLEETICSARCTDNLDDPSRADVYKPQLGYISTLNSYDLPILRFIRLSALRGSIRQNAMVL
Csa11g070580.1_CsCruA-1-G1  (246) RGNIVRVQGPFGVIRPPLR-GQRPQEEEEE-----RVGNGLEETICSARSVDNLDDPSRADVYKPQLGYISTLNSYDLPILRFIRLSALRGSIRQNAMVL
Csa11g070590.1_CsCruA-2-G1  (246) RGNIVRVQGPFGVIRPPLR-GQRPQEEEEE-----RVGNGLEETICSARSVDNLDDPSRADVYKPQLGYISTLNSYDLPILRFIRLSALRGSIRQNAMVL
Csa18g009670.1_CsCruA-1-G2  (243) RGNIVRVQGPFGVIRPPLR-GQRPQEEEEE-----RVGNGLEETICSARSVDNLDDPSRADVYKPQLGYISTLNSYDLPILRFIRLSALRGSIRQNAMVL
                     AtCRB  (238) RGNIVKVNGPFGVIRPPLRRGEGGQQPHEI-------ANGLEETLCTMRCTENLDDPSDADVYKPSLGYISTLNSYNLPILRLLRLSALRGSIRKNAMVL
Csa03g005050.1_CsCruB-1-G3  (238) RGNIVKVKGPFGVIRPPLRRGQGGQQPQEK-------ANGLEETLCTMRCTENLDDPSDADVYKPSLGYISTLNSYNLPILRLLRLSALRGSIRNNAMVL
Csa14g004960.1_CsCruB-1-G2  (238) RGNIVKVKGPFGVIRPPLRRGQGGQQPQEK-------ANGLEETLCTMRCTENLDDPSDADVYKPSLGYISTLNSYNLPILRLLRLSALRGSIRNNAMVL
Csa17g006950.1_CsCruB-1-G1  (240) RGNIVKVKGPFSVIRPPLRRGQGGQQPQEK-------ANGLEETLCTMRCTENLDDPSDADVYTPSLGYINTVNSYNLPILRLLHLSALRGSIRNNAMVL
                     AtCRC  (298) RGNIVRVKGPFQVVRPPLRQPYESEEWRHPR---SPQGNGLEETICSMRSHENIDDPARADVYKPSLGRVTSVNSYTLPILEYVRLSATRGVLQGNAMVL
Csa10g014100.1_CsCruC-1-G2  (267) RGNIVRVKGPFQVVRPPLRQPYESEERRRPR---GPQDNGLEETIYSMRSHENIDDPARADVYKPNIGRVTSVNSYTLPILQYIRLSATRGHIQGNAMVL
Csa11g015240.1_CsCruC-1-G1  (267) RGNIVRVKGPFQVVRPPLRQPYESEERRRPR---GPQDNGLEETICSMRSHENIDDPARADVYKPNIGRVTSVNSYTLPILQYIRLSATRGLIQGNAMVL
Csa12g021990.1_CsCruC-1-G3  (267) RGNIVRVKRPFQVVRPPLRQPYESEERRRPR---GPQDNGLEETIYSMRSHENIDDPARADVYKPNIGRVTSVNSYTLPILQYIRLSATRGVIQDNAMVL
Csa03g005060.1_CsCruD-1-G3  (245) RGNIVRANGPLHFVIPQPR-QWQQDGIA----------NGIEETYCTARLHENIEDPERSDLFSTRAGRMSTLNSLNLPVLRQVRLNAVRGYLYSGGMVL
Csa14g004970.1_CsCruD-1-G2  (245) RGNIVRANGPLHFVIPQPR-QWQQDGIA----------NGIEETYCTARLHENIDDPERSDLFSTRAGRMSTLNSLNLPVLRQVRLNAVRGYLYSGGMVL
Csa17g006960.1_CsCruD-1-G1  (247) RGNIVRANGPLHFVIPQPRQWQQQDGIA----------NGIEETYCTARLHENIDDPERSDLFSTRAGRISTLNSLNLPVLRLVRLNAVRGYLYSGGMVL
                                                     ???????????????????#
                                                     HVR-IV

                     AtCRA  (344) PQWNANANAILYVTDGEAQIQIVNDNGNRVFDGQVSQGQLIAVPQGFSVVKRATSNRFQWVEFKTNANAQINTLAGRTSVLRGLPLEVITNGFQISPEEA
Csa11g070580.1_CsCruA-1-G1  (340) PQWNANANSVLYVTDGEAQVQIVNDNGDRVFDGQVSQGQLIVVPQGFSVVKRATSDQFRWVEFKTNANAQINTLAGRTSVLRGLPLAVITNGFEISPEEA
Csa11g070590.1_CsCruA-2-G1  (340) PQWNANANAVLYVTDGEAQVQIVNDNGDRVFDGQVSQGQLIVVPQGFSVVKRATSDQFRWVEFKTNANAQINTLAGRTSVLRGLPLAVITNGFEISPEEA
Csa18g009670.1_CsCruA-1-G2  (337) PQWNANANSVLYVTDGEAQVQIVNDNGDRVFDGQVSQGQLIVVPQGFSVVKRATSDQFRWVEFKTNANAQINTLAGRTSVLRGLPLAVITNGFQISHEEA
                     AtCRB  (331) PQWNVNANAALYVTNGKAHIQMVNDNGERVFDQEISSGQLLVVPQGFSVMKHAIGEQFEWIEFKTNENAQVNTLAGRTSVMRGLPLEVITNGYQISPEEA
Csa03g005050.1_CsCruB-1-G3  (331) PQWNVNANSALYVTNGKAHIQMVNDNGDRVFDQEISNGQLLVVPQGFSVMKRATSEQFQWIEFKTNENAQVNSLAGRTSVMTGLPLEVITNGFQISPQDA
Csa14g004960.1_CsCruB-1-G2  (331) PQWNVNANSALYVTNGKAHIQMVNDNGDRVFDQEISNGQLLVVPQGFSVMKRATSEQFQWIEFKTNENAQVNSLAGRTSVMTGLPLEVITNGFQISPQDA
Csa17g006950.1_CsCruB-1-G1  (333) PQWNVNANSALYVTNGRAHIQMVNDNGDRVFDQEVSNGQLLIVPQGFSVMKRATSEQFQWIEFKTNENAQVNSLAGRTSVMTGLPLEVITNGFQISPQEA
                     AtCRC  (395) PKYNMNANEILYCTGGQGRIQVVNDNGQNVLDQQVQKGQLVVIPQGFAYVVQSHGNKFEWISFKTNENAMISTLAGRTSLLRALPLEVISNGFQISPEEA
Csa10g014100.1_CsCruC-1-G2  (364) PKYNMNANEILYCTGGQGRIQVVNDNGQNVLDQQVQKGQLVVIPQGFAYVVQSHGNNFEWISFKTNENAMISTLAGRTSLLRGLPLAVISNGFQVSPEEA
Csa11g015240.1_CsCruC-1-G1  (364) PKYNMNANEILYCTGGQGRIQVVNDNGQNVLDQQVQKGQLVVIPQGFAYVVQSHGNNFEWISFKTNENAMISTLAGRTSLLRGLPLAVISNGFQVSPEEA
Csa12g021990.1_CsCruC-1-G3  (364) PKYNMNANEILYCTGGQGRIQVVNDNGQNVLDQQLQKGQLVVIPQGFAYVVQSHGNNFEWISFKTNENAMISTLAGRTSLLRGLPLEVISNGFQVSPEEA
Csa03g005060.1_CsCruD-1-G3  (334) PQWTANAHTVLYVTGGQAKIQVVDDNGQSVFNEQVGQGQLLVIPQGFAVVKTAGETGFEWISFKTNDNAYINTLSGQTSYLRAVPLDVVKASYGVTEEEA
Csa14g004970.1_CsCruD-1-G2  (334) PQWTANAHTVLYVTGGQAKIQVVDDNGQSVFNEQMGQGQLLVIPQGFAVVKTAGETGFEWISFKTNDNAYINTLSGQTSYLRAVPLDVVKASYGVTEEEA
Csa17g006960.1_CsCruD-1-G1  (337) PQWTANAHTVLYVTGGQAKIQVVDDNGQSVFNEQVGQGQLLVIPQGFAVVKTAGETGFEWISFKTNDNAYINTLSGQTSYLRAVPLDVVKASYGVTEEEA

                     AtCRA  (444) RRVKFNTLETTLTHSSGPASYGRPRVAAA--
Csa11g070580.1_CsCruA-1-G1  (440) KRVKFNTLETTLTHSSGPASYGRPRVAAA--
Csa11g070590.1_CsCruA-2-G1  (440) KRVKFNTLETTLTHSSGPASYGRPRVAAA--
Csa18g009670.1_CsCruA-1-G2  (437) KRVKFNTLETTLTHSSGPASYGRPRVATA--
                     AtCRB  (431) KRVKFSTIETTLTHSS-PMSYGRPRA-----
Csa03g005050.1_CsCruB-1-G3  (431) KRVKFSTMETTLTHSSGPMSYGRPRV-----
Csa14g004960.1_CsCruB-1-G2  (431) KRVKFSTMETTLTHSSGPMSYGRPRV-----
Csa17g006950.1_CsCruB-1-G1  (433) KQVKFSTMETTLTHSSGPMSYGRPRV-----
                     AtCRC  (495) RKIKFNTLETTLTRAAGRQQQQLIEEIVEA-
Csa10g014100.1_CsCruC-1-G2  (464) RRIKFNTLETILTRSAGRQQ-----------
Csa11g015240.1_CsCruC-1-G1  (464) RRIKFNTLETTLTRSAGRQQQ-YIEEIVEA-
Csa12g021990.1_CsCruC-1-G3  (464) RRIKFNTLETTLTRSSGRQQQ-YIEEIVEA-
Csa03g005060.1_CsCruD-1-G3  (434) KRIKFSQQEAMLAMTPSSSS-----------
Csa14g004970.1_CsCruD-1-G2  (434) KRIKFSQQEAMLAMTPSSSS-----------
Csa17g006960.1_CsCruD-1-G1  (437) KRIKFSQQEAMLAMTPSSSS-----------
                                              ???????????????????
                                              HVR-V
```

S = signal peptide
? = hypervariable region (HVR)
# = protease cleavage site
Shading depicts blocks of identical (black) or conserve (gray) amino acids
S = predicted serine phosphorylation T = predicted threonine phosphorylation Y = predicted tyrosine phosphorylation
