# Supplementary material for: Genetic variation and structural diversity in major seed proteins among and within Camelina species
Source: Planta. 2022 Oct 6;256(5):93. doi: 10.1007/s00425-022-03998-w (PMC9537204; doi:10.1007/s00425-022-03998-w)
Supplement: Supplementary file 12 — Supplementary file12 (PDF 609 KB) [file 425_2022_3998_MOESM12_ESM.pdf]

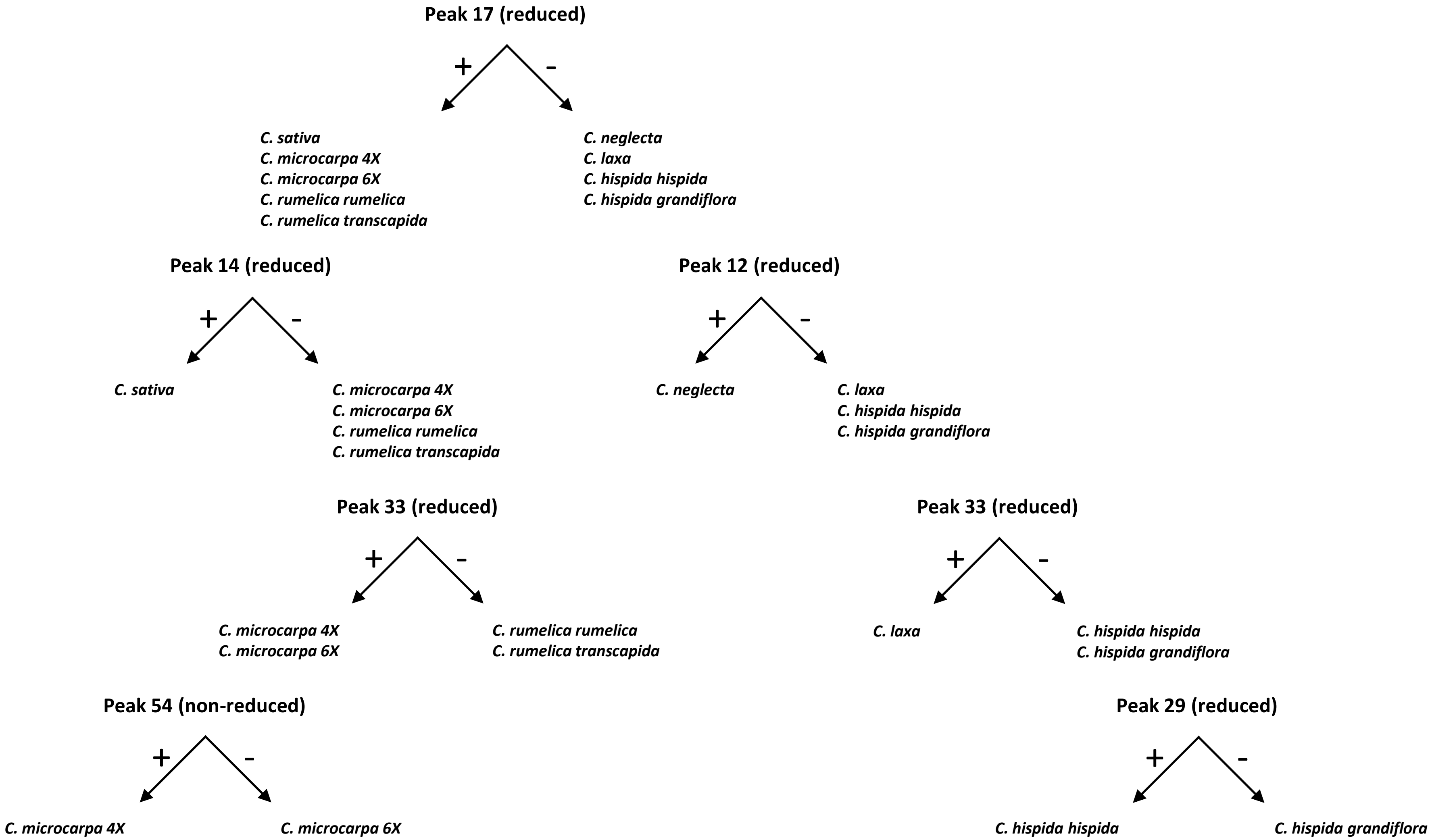

Supplemental Fig. S1. Differentiation of *Camelina* species by seed protein profile
