# Supplementary figures and images for: Genetic variation and structural diversity in major seed proteins among and within Camelina species
Source: Planta. 2022 Oct 6;256(5):93. doi: 10.1007/s00425-022-03998-w (PMC9537204; doi:10.1007/s00425-022-03998-w)

Supplemental Fig. S2a. *C. sativa* cruciferin phylogeny.

Nucleotide

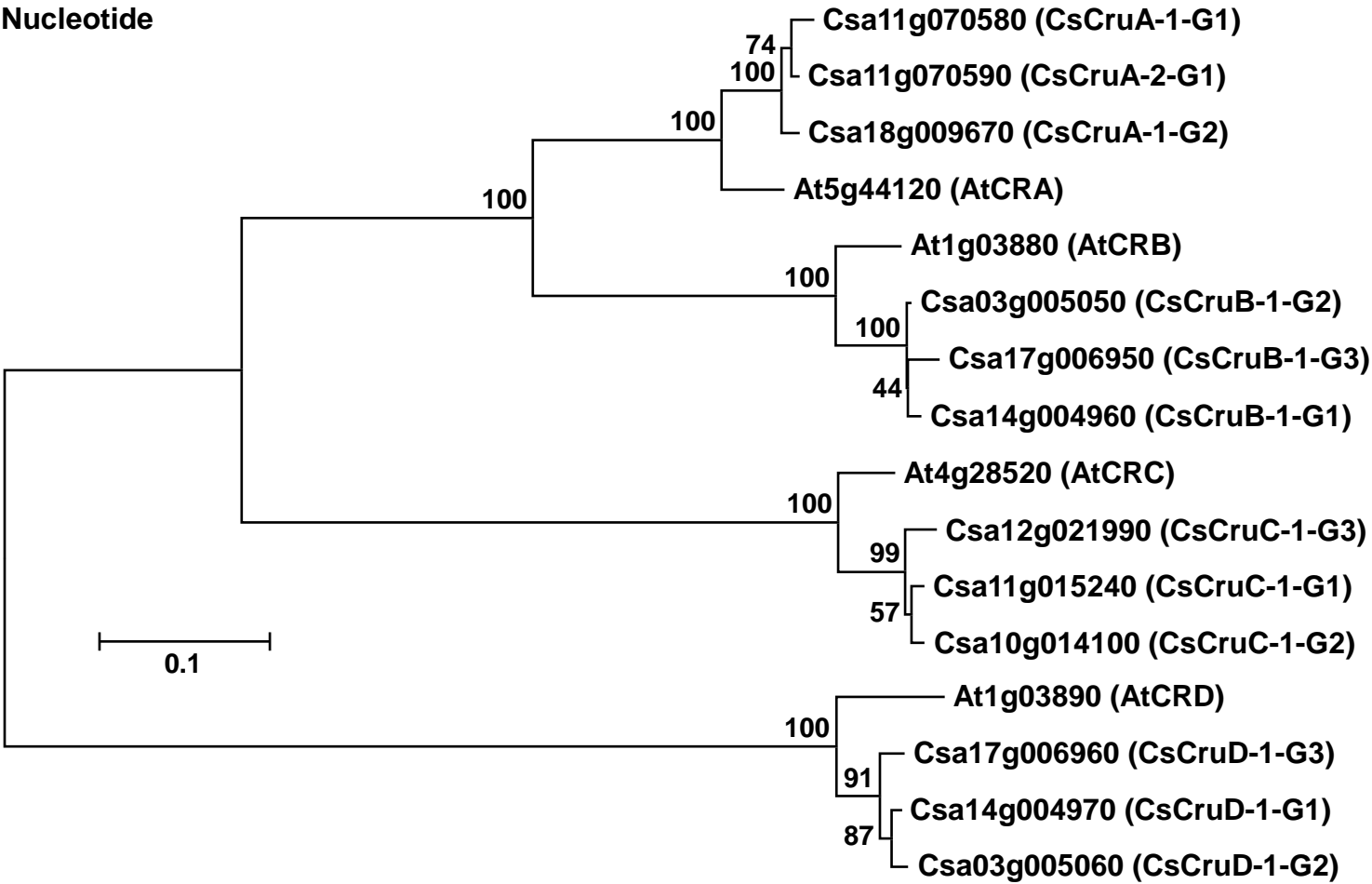

Protein

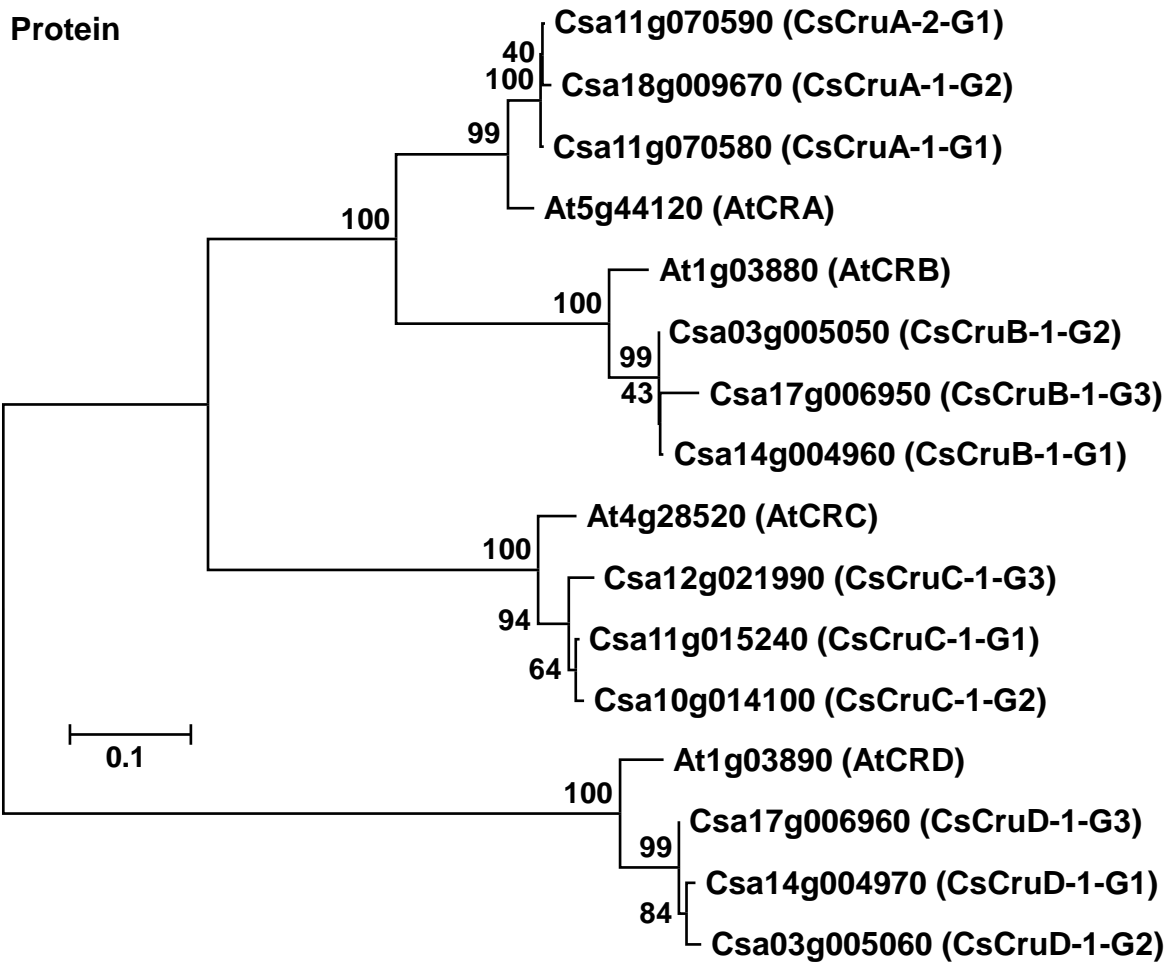

Supplement: Supplementary file 13 — Supplementary file13 (PDF 68 KB) [file 425_2022_3998_MOESM13_ESM.pdf]

Supplemental Fig. S2b. *C. sativa* vicilin phylogeny.

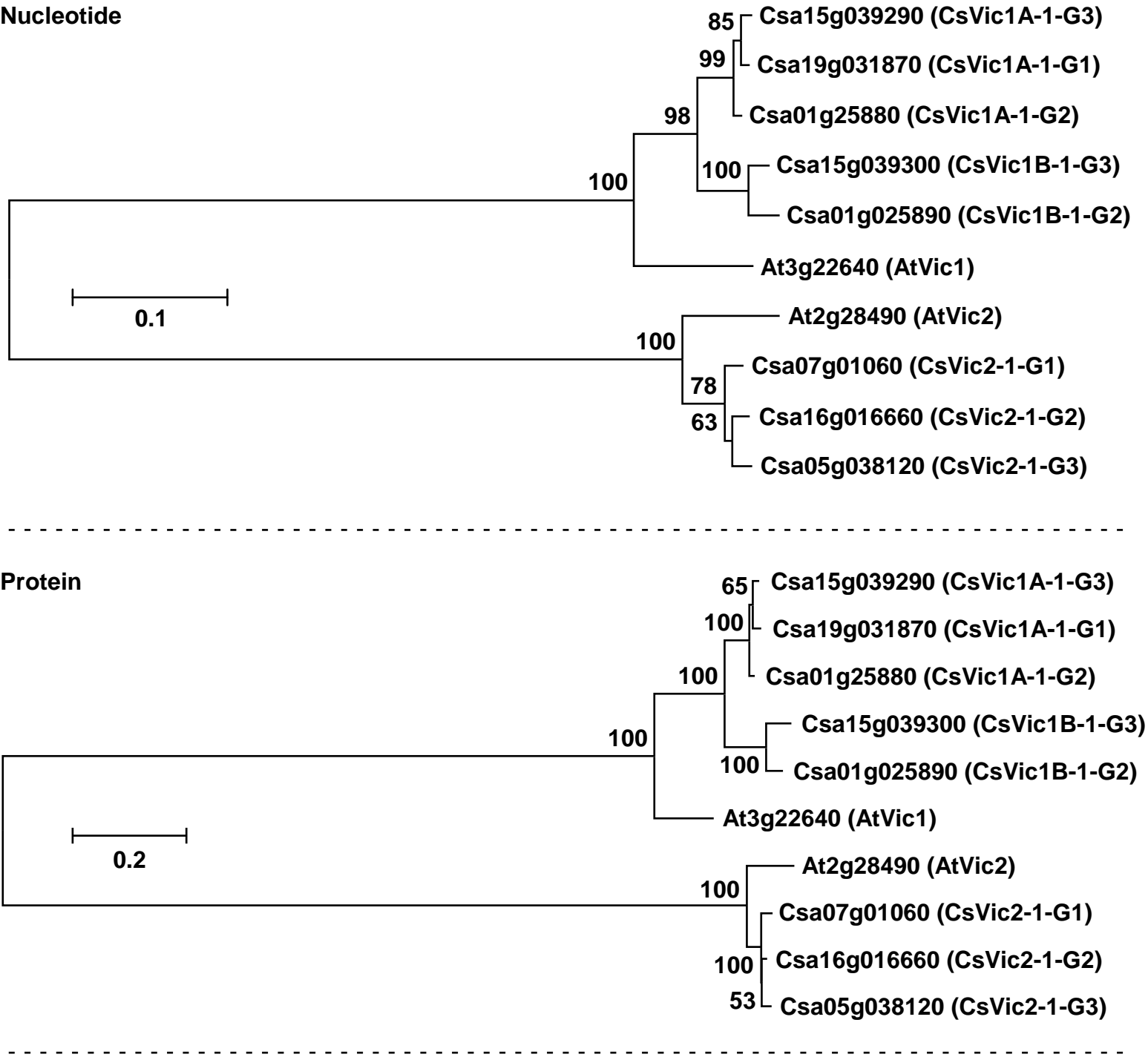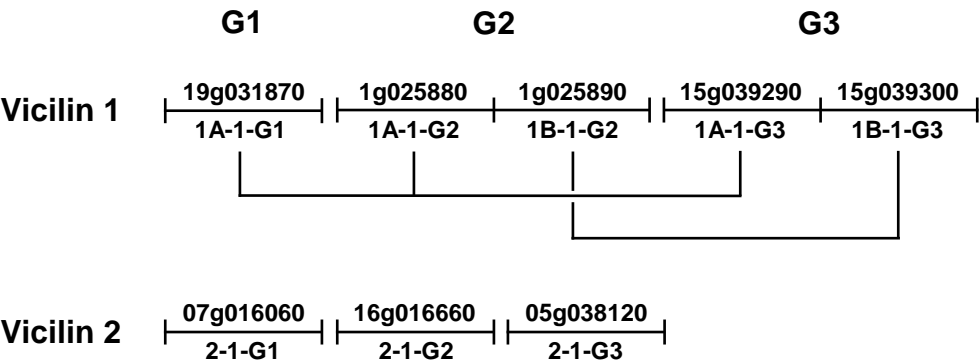

Supplement: Supplementary file 14 — Supplementary file14 (PDF 68 KB) [file 425_2022_3998_MOESM14_ESM.pdf]

Supplemental Fig. S2c. *C. sativa* napin phylogeny.

Nucleotide

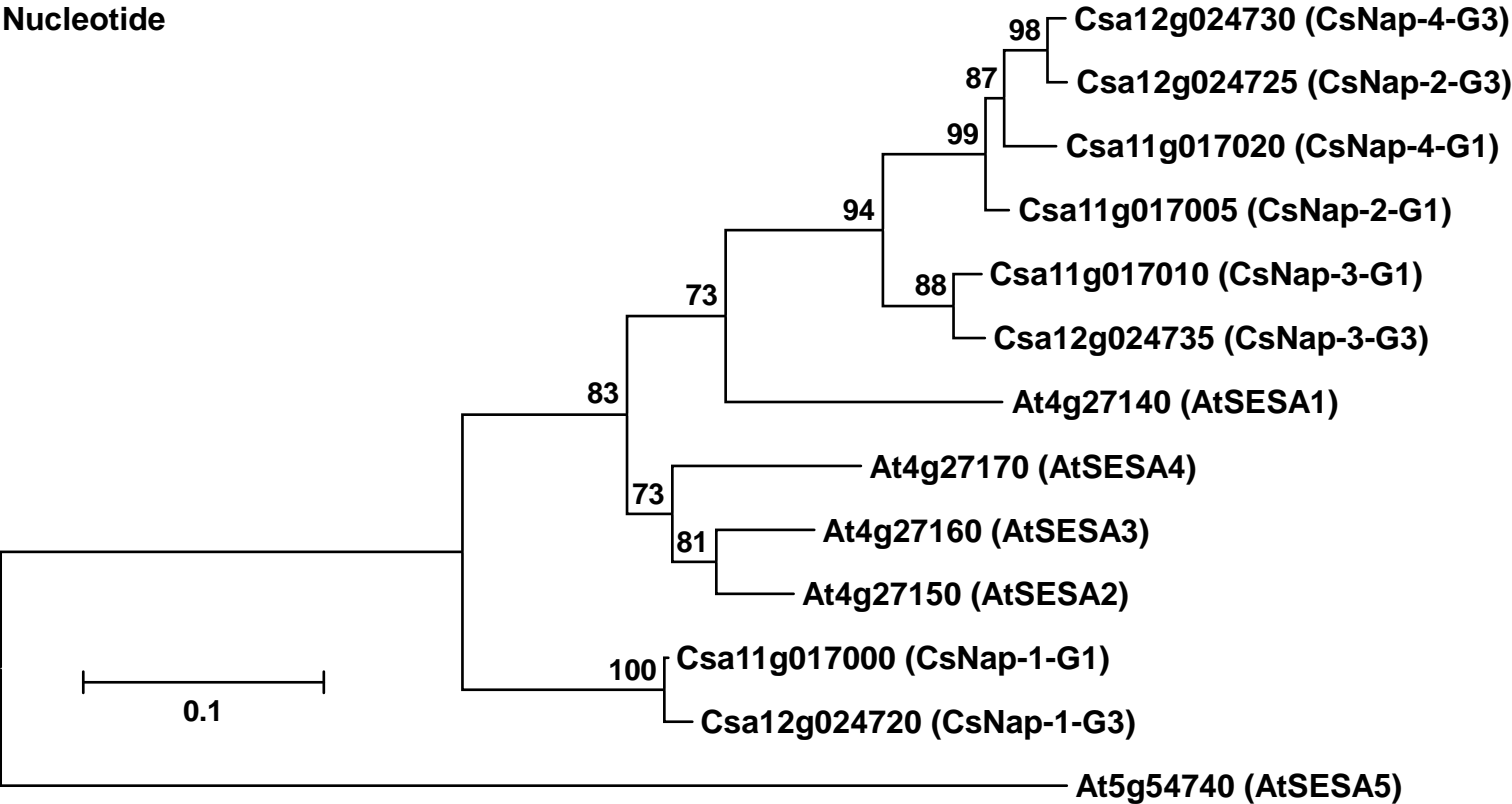

Protein

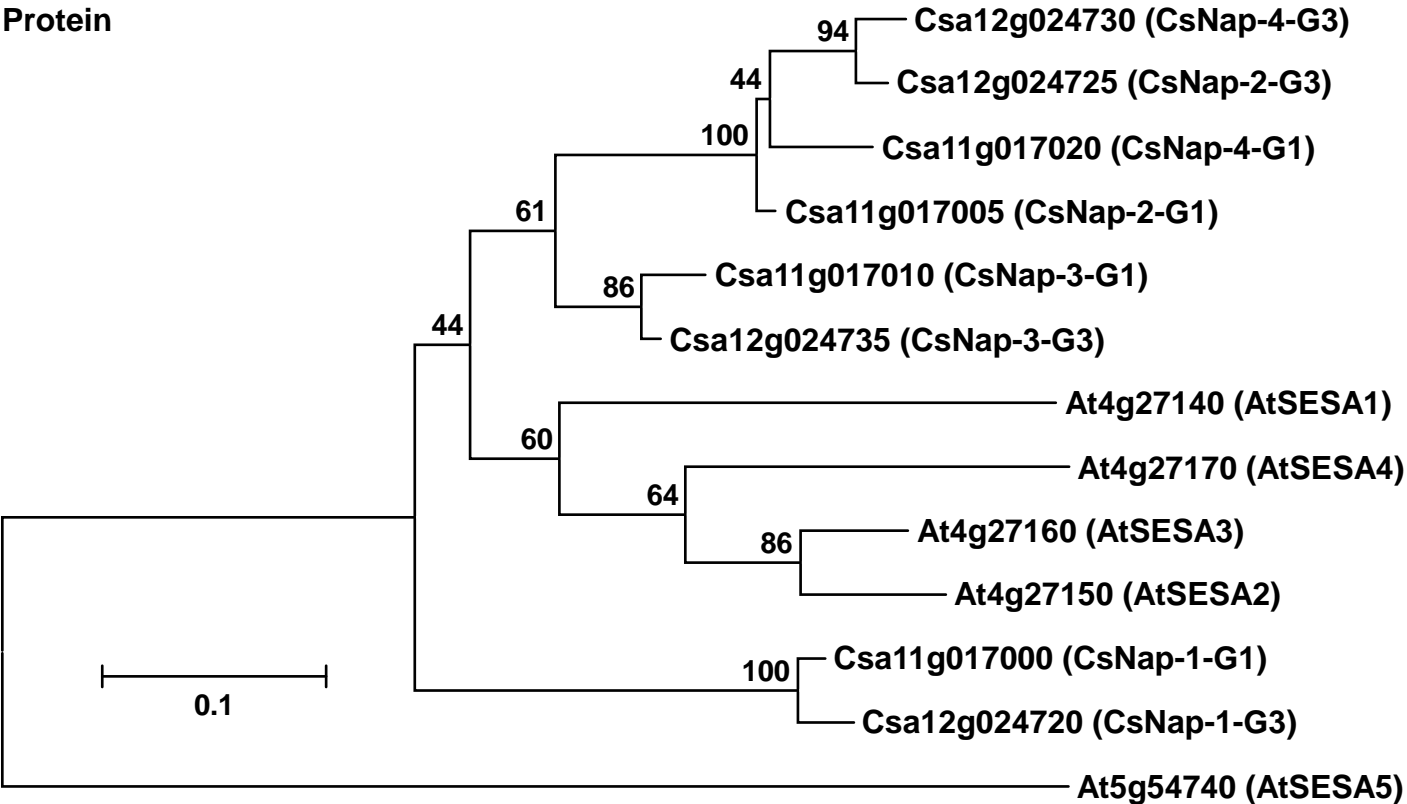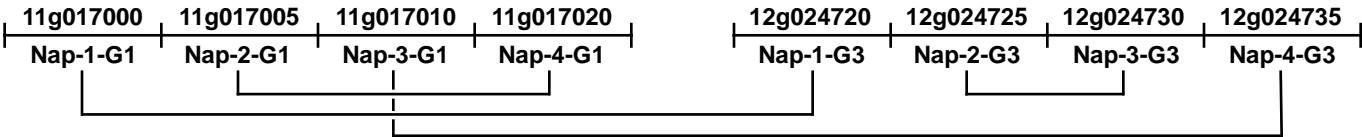

Supplement: Supplementary file 15 — Supplementary file15 (PDF 83 KB) [file 425_2022_3998_MOESM15_ESM.pdf]

**Supplemental Fig. S2d.** *C. sativa* oleosin phylogeny.

**Nucleotide**

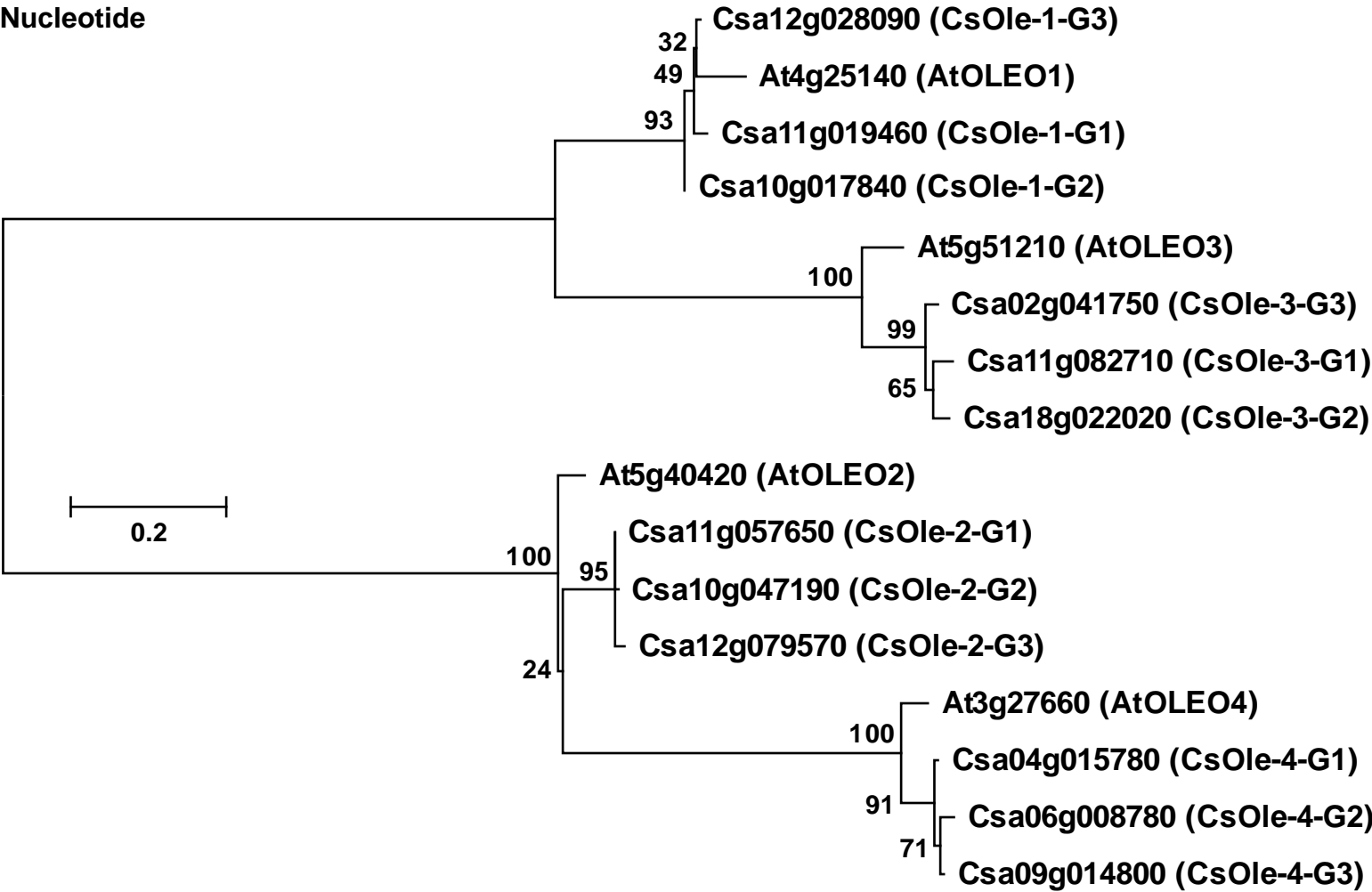

**Protein**

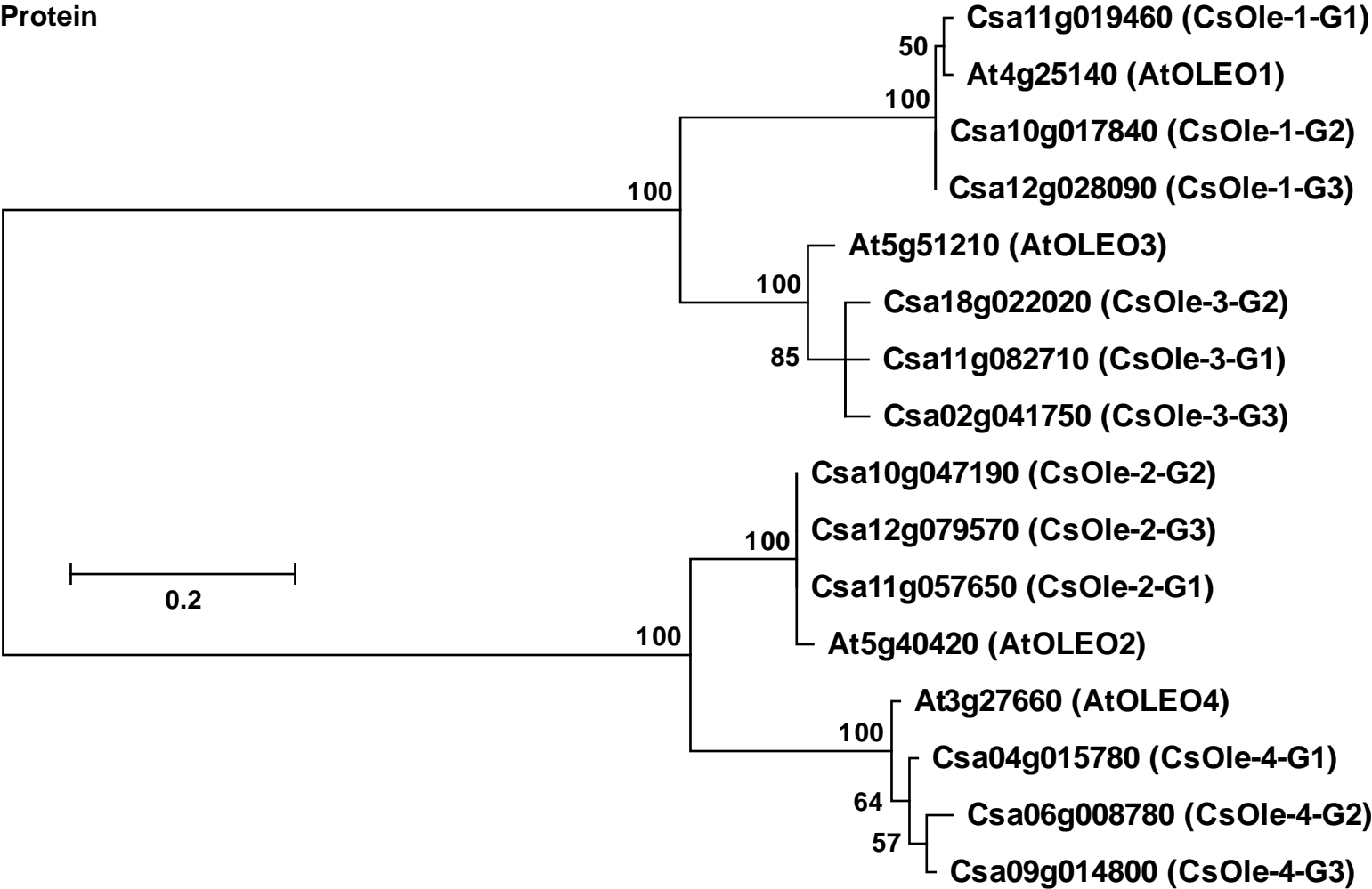

Supplement: Supplementary file 16 — Supplementary file16 (PDF 74 KB) [file 425_2022_3998_MOESM16_ESM.pdf]
