# Supplementary material for: Genetic variation and structural diversity in major seed proteins among and within Camelina species
Source: Planta. 2022 Oct 6;256(5):93. doi: 10.1007/s00425-022-03998-w (PMC9537204; doi:10.1007/s00425-022-03998-w)
Supplement: Supplementary file 17 — Supplementary file17 (PDF 347 KB) [file 425_2022_3998_MOESM17_ESM.pdf]

Supplemental Fig. S3. Alignment of *C. sativa* napins.

```
                                  1                                                                                                  100
Csa11g017000 (Cs2S-1-G1)    (1)   MANKLFLVSATLALCFLLTNASIYRTVVEFDEDDATNRQFRPQQKCQREFQQQQHLRACQSWMRLQTRQG-RGSGPALDDEFDFQDDIENPQQRHPVLQQ
CsSESA8 (JX191889)          (1)   MANKLFLVSATLALCFLLTNASIYRTVVEFDEDDATNRQFRPQQKCQREFQQQQHLRACQSWMRQQTRQG-RGSGPALDDEFDFQDDIENPQQRHPVLQQ
Csa12g024720 (Cs2S-1-G3)    (1)   MANKLFLVSATLALCFLLTNASIYRTVVEFDEDDATNRQFRPQQKCQREFQQQQHLRACQSWMRQQSRQG-RGSGPALDDEFDFQDDIENPQRRDPVLQQ
CsSESA7 (JX191888)          (1)   MANKLFLVSATLALCFLLTNASIYRTVVEFDEDDATNRQFRPQQKCQREFQQQQHLRACQSWMRQQSRQG-RGSGPALDDEFDFQDDIENPQRRDPVLQQ
Csa11g017010 (Cs2S-3-G1)    (1)   MANKLFLVGATLALCFLLTNASIYRTVVEFDED-ATN-PMGPSQRCQREFQQSQHLRACQQLMRSQMRQG-RGG-----DEYGLEDDMENPQRPQQILQQ
CsSESA4 (JX191885)          (1)   MANKLFLVGATLALCFLLTNASIYRTVVEFDED-ATN-PMGPSQRCQREFQQSQHLRACQQLMRSQMRQG-RGG-----DEYGLEDDMENPQRPQQILQQ
Csa12g024735 (Cs2S-4-G3)    (1)   MANKLFLVGATLALCFLLTNASIYRTVVEFDED-ATN-PMGPSQRCQREFQQSQHLRACQQLMRRQMRQGGRGD-----DEFDLEDDMENPQRPQQILQQ
CsSESA3 (JX191884)          (1)   MANKLFLVGATLALCFLLTNASIYRTVVEFDED-ATN-PMGPSQRCQREFQQSQHLRACQQLMRRQMRQGGRGD-----DEFDLEDDMENPQRPQQILQQ
Csa11g017005 (Cs2S-2-G1)    (1)   MANKIFLVCATLALCFLLTNASIYRTVVEFDED-ATN-PMGPRQRCQREFQQSQHLRACQQLMRRQMRQG-RG------DEYDWEEDMENPQRPQQLLQQ
CsSESA5 (JX191886)          (1)   MANKIFLVCATLALCFLLTNASIYRTVVEFDED-ATN-PMGPRQRCQREFQQSQHLRACQQLMRRQMRQG-RG------DEYDWEEDMENPQRPQQLLQQ
Csa11g017020 (Cs2S-4-G1)    (1)   MANKLFLVGATLALCFLLTNASIYRTVVEFDED-ATN-PMGPRQRCQREFQQSQHLRACQQFMRRQARQG-RGG-----DEYDWEEDMETSRRPQQLLQQ
CsSESA2 (JX191883)          (1)   MANKLFLVGATLALCFLLTNASIYRTVVEFDED-ATN-PMGPRQRCQREFQQSQHLRACQQFMRRQARQG-RGG-----DEYDWEEDMETSRRPQQLLQQ
Csa12g024725 (Cs2S-2-G3)    (1)   MANKLFLVCATLAVCFLLTNASIYRTVVEFDED-ATN-PMGPRQRCQREFQQSQHLRACQELMRRQMRQG-RGG-----DEYDWEEDMENPQRRQQVLEQ
CsSESA1 (JX191882)          (1)   MANKLFLVCATLAVCFLLTNASIYRTVVEFDED-ATN-PMGPRQRCQREFQQSQHLRACQELMRRQMRQG-RGG-----DEYDWEEDMENPQRRQQVLEQ
Csa12g024730 (Cs2S-3-G3)    (1)   MANKLFLVCATLALCFLLTNASIYRTVVEFDED-TTN-PMGPRQRCQREFQQSQHLRACQELMRRQARQGGRGG-----DEYDWEEDMENPQRRQQVLEQ
CsSESA6 (JX191887)          (1)   MANKLFLVCATLALCFLLTNASIYRTVVEFDEE-TTN-PMGPRQRCQREFQQSQHLRACQELMRRQARQGGRGG-----DEYDWEEDMENPQRRQQVLEQ
                                  sssssssssssssssssssssppppppppppppppppSSSSSSSSSSSSSSSSSSSSSSSSSSSSSSSSSSSSSSSSSSSppppppppppLLLLLLLLLL

                                  101                                                                  173
Csa11g017000 (Cs2S-1-G1)  (100)   CCNELQQEDPECVCPTLKQAARAVRLQG----QQGPYQASQILQTAKRLPNICNIPQVGVCPFRASP---YY-
CsSESA8 (JX191889)        (100)   CCNELQQEDPECVCPTLKQAARAVRLQG----QQGPYQASQILQTAKRLPNICNIPQVGVCPFRASP---YY-
Csa12g024720 (Cs2S-1-G3)  (100)   CCNELQQQDPECVCPTLKQAARAVRLQG----QQGPYQASQILQTAKRLPNICNIPQVGVCPFRASP---YY-
CsSESA7 (JX191888)        (100)   CCNELQQQDPECVCPTLKQAARAVRLQG----QQGPYQASQILQTAKRLPNICNIPQVGVCPFRASP---YY-
Csa11g017010 (Cs2S-3-G1)   (93)   CCNELRQEDPVCVCPTLRQAAQSVRLQG----QHGPFQARKIYQTAKHLPNVCNIPQVGVCPFQTIPFSPYY-
CsSESA4 (JX191885)         (93)   CCNELRQEDPVCVCPTLRQAAQSVRLQG----QHGPFQARKIYQTAKHLPNVCNIPQVGVCPFQTIPFSPYY-
Csa12g024735 (Cs2S-4-G3)   (94)   CCNELRQEDPVCVCPTLRQAAQSVRLQG----QHGPFQARKIYQTAKHLPNVCRIPQVGVCPFQTIPSSPYY-
CsSESA3 (JX191884)         (94)   CCNELRQEDPVCVCPTLRQAAQSVRLQG----QHGPFQARKIYQTAKHLPNVCRIPQVGVCPFQTIPSSPYY-
Csa11g017005 (Cs2S-2-G1)   (92)   CCNELRQEDPVCVCPTLKQAARSVSIQGQGQGQQRQSQARQIYQTAKHLPNVCNIPQVDVCPFQIPSGPSYY-
CsSESA5 (JX191886)         (92)   CCNELRQEDPVCVCPTLKQAARSVSIQGQGQGQQRQSQARQIYQTAKHLPNVCNIPQVDVCPFQIPSGPSYY-
Csa11g017020 (Cs2S-4-G1)   (93)   CCNELRQEDPVCVCPTLQQAARSVSIQGQGQGQQRQSQARQIYQTAKHLPNVCNIPQVDVCPFQIPSGPSYY-
CsSESA2 (JX191883)         (93)   CCNELRQEDPVCVCPTLQQAARSVSIQGQGQGQQRQSQARQIYQTAKHLPNVCNIPQVDVCPFQIPSGPSYY-
Csa12g024725 (Cs2S-2-G3)   (93)   CCNELRQEDPACVCPTLQQAARSVSIQGQGQGQQRQSQARQVYQTAKHLPNVCRIPQVDVCPFQIPSGPSYY-
CsSESA1 (JX191882)         (93)   CCNELRQEDPACVCPTLQQAARSVSIQGQGQGQQRQSQARQVYQTAKHLPNVCRIPQVDVCPFQIPSGPSYY-
Csa12g024730 (Cs2S-3-G3)   (94)   CCNELRQEDPACVCPTLQQAARSVSIQGQGQGQQRQSQARQIYQTAKHLPNVCRIQQVDVCPFQIPSGPSYY-
CsSESA6 (JX191887)         (94)   CCNELRQEDPACVCPTLQQAARSVSIQGQGQGQQRQSQARQIYQTAKHLPNVCRIQQVDVCPFQIPSGPSYY-
                                  LLLLLLLLLLLLLLLLLLLLLLLLLLLLLLLLLLLLLLLLLLLLLLLLLLLLLLLLLLLLLLLLLLLLLLpp
```

Colours indicate blocks of identical amino acids.
s = signal peptide
S = small subunit
L = large subunit
p = processed peptide
