# Supplementary material for: Genetic variation and structural diversity in major seed proteins among and within Camelina species
Source: Planta. 2022 Oct 6;256(5):93. doi: 10.1007/s00425-022-03998-w (PMC9537204; doi:10.1007/s00425-022-03998-w)
Supplement: Supplementary file 18 — Supplementary file18 (PDF 255 KB) [file 425_2022_3998_MOESM18_ESM.pdf]

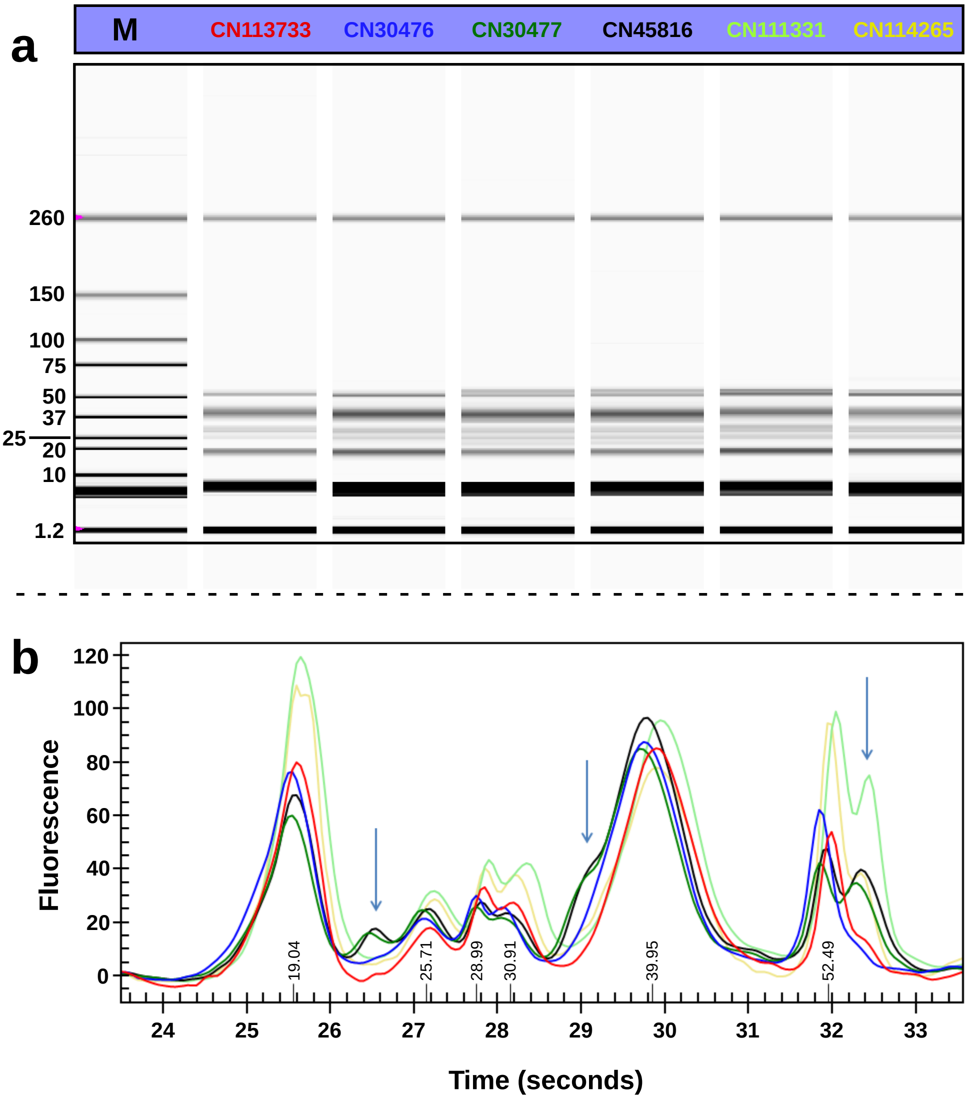

Supplemental Fig. S4. Seed protein profiles of *C. sativa* lines used for RNA-Seq analysis. **a** shows digital profiles based on the capillary electrophoresis electropherograms (**b**). M, molecular weight markers (kDa). Arrows indicate major differences between groups.
