# Supplementary material for: Genetic variation and structural diversity in major seed proteins among and within Camelina species
Source: Planta. 2022 Oct 6;256(5):93. doi: 10.1007/s00425-022-03998-w (PMC9537204; doi:10.1007/s00425-022-03998-w)
Supplement: Supplementary file 19 — Supplementary file19 (PDF 3533 KB) [file 425_2022_3998_MOESM19_ESM.pdf]

Supplemental Fig. S5. *B. napus*, *A. thaliana* and *C. sativa* cruciferin homology models.

3KGL assembly 1: Structural Template

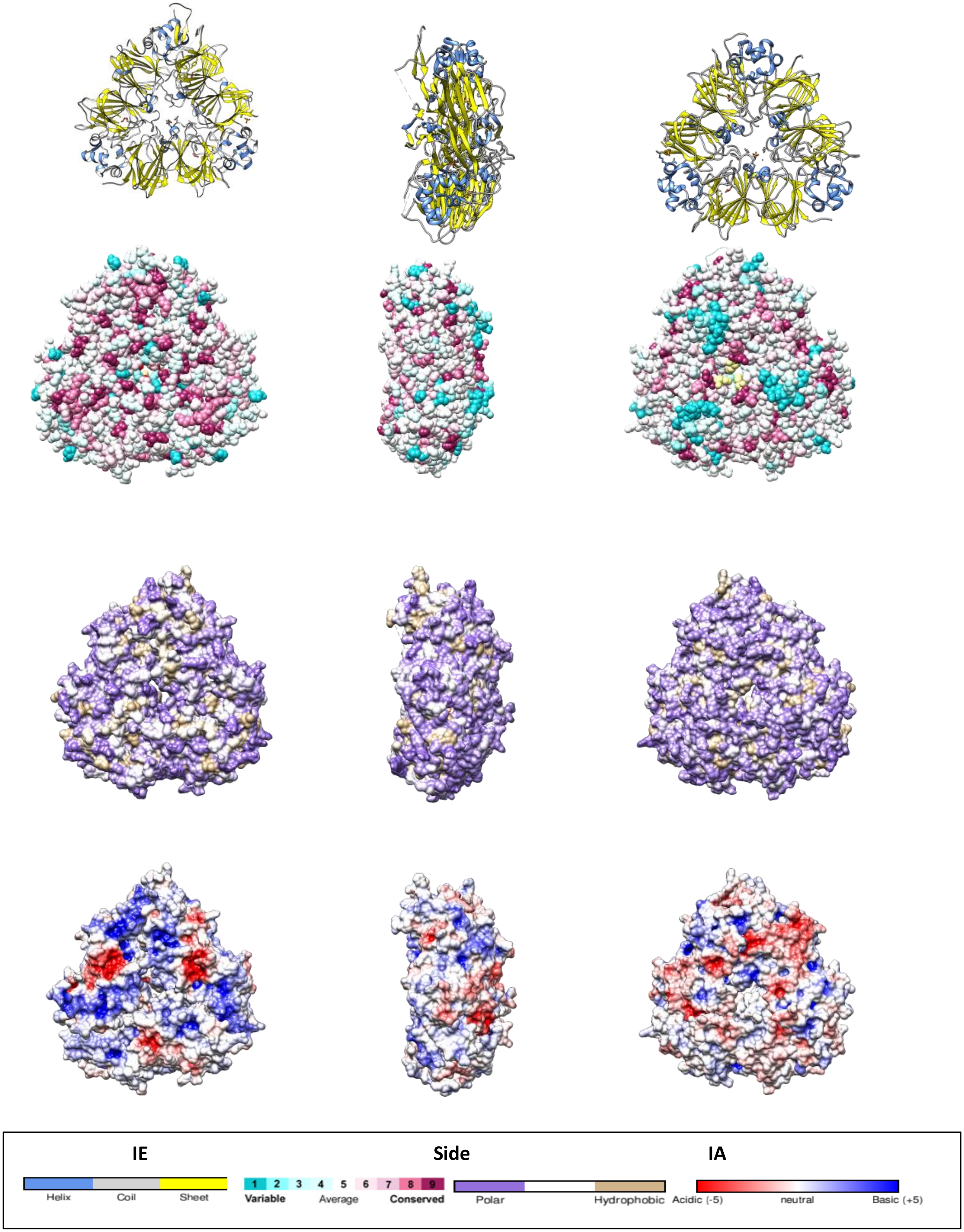

AtCruA

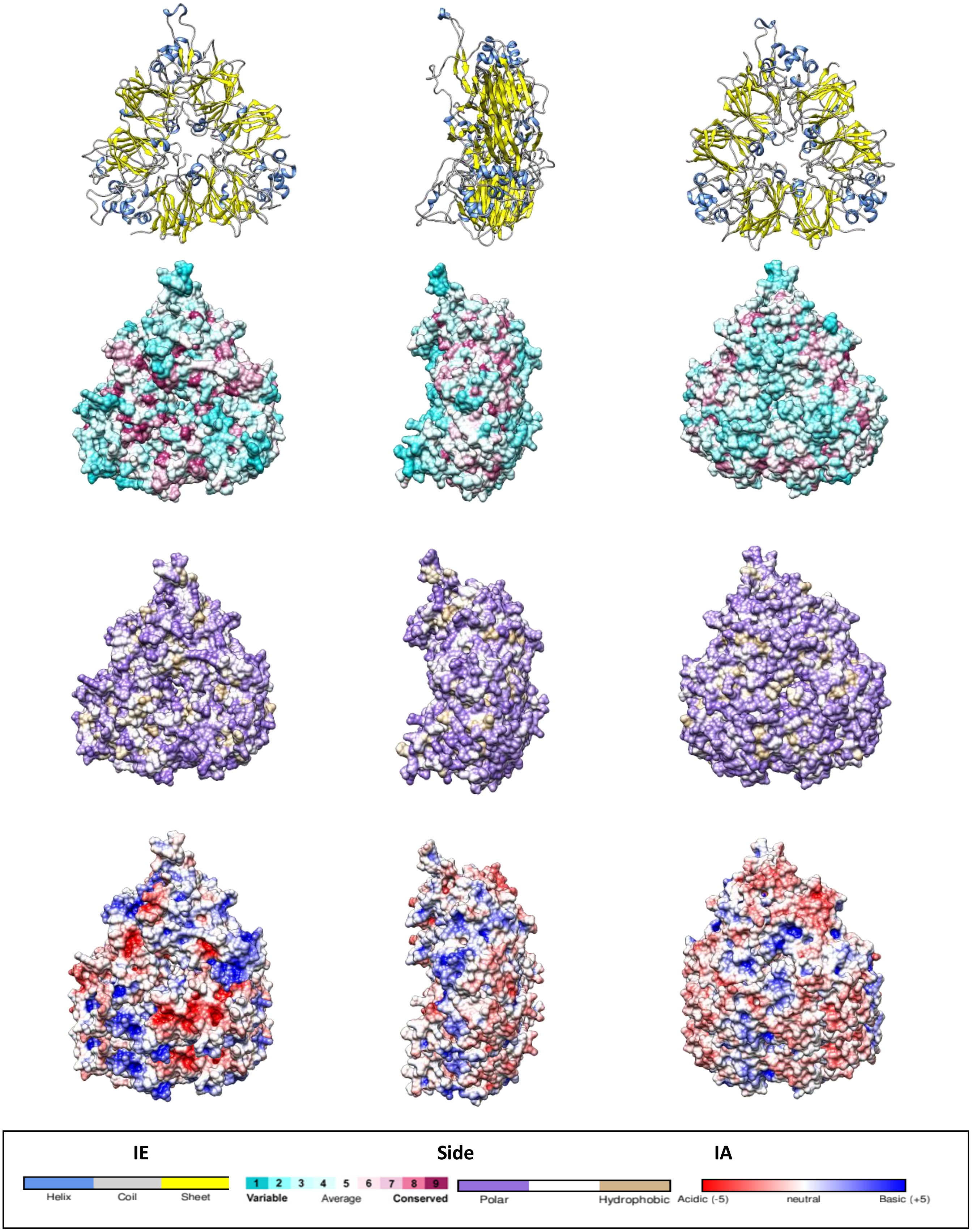
IE
Side
IA
Helix
Coil
Sheet
1 2 3 4 5 6 7 8 9
Variable
Average
Conserved
Polar
Hydrophobic
Acidic (-5)
neutral
Basic (+5)

AtCruB

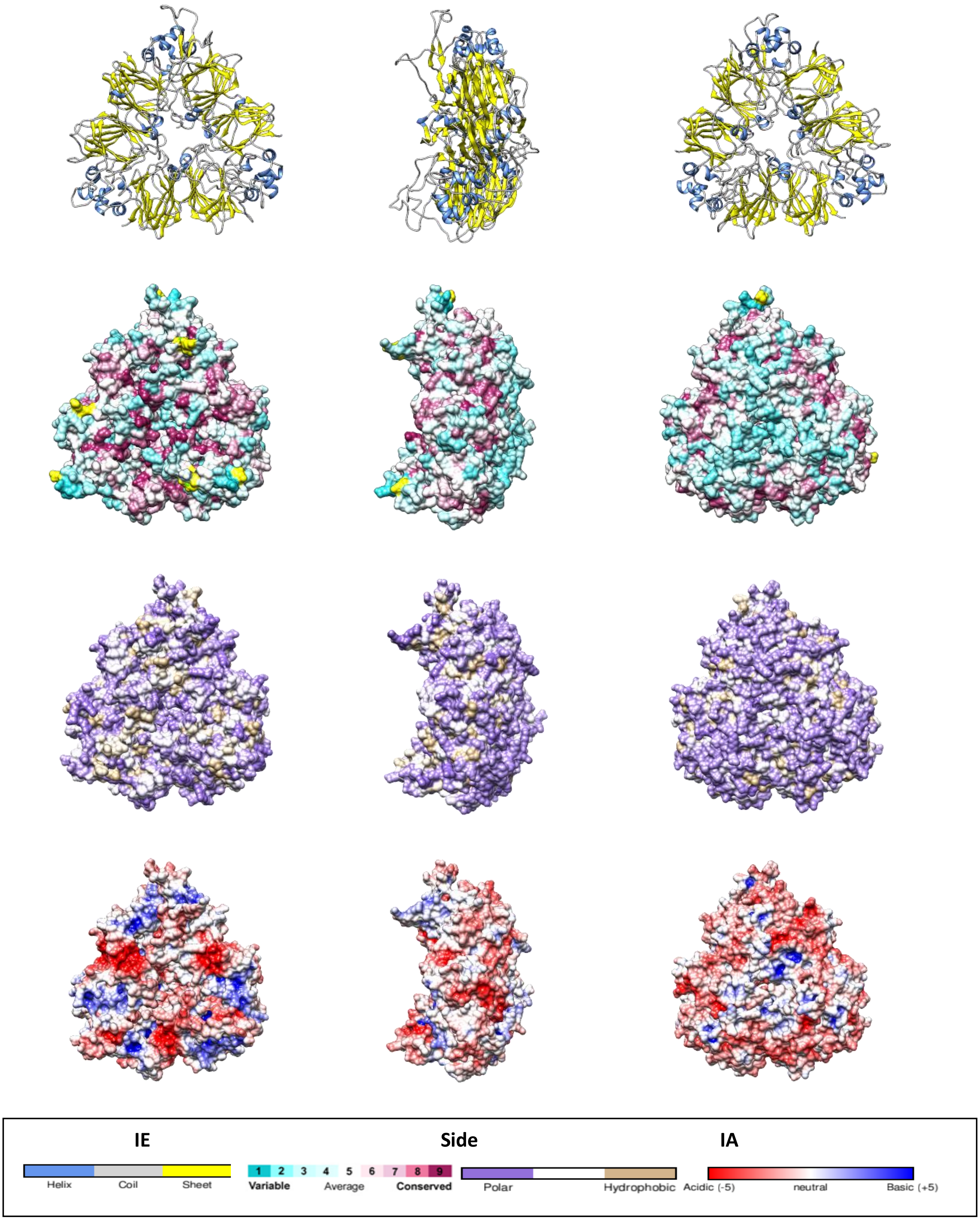
IE
Side
IA
Helix
Coil
Sheet
1 2 3 4 5 6 7 8 9
Variable
Average
Conserved
Polar
Hydrophobic
Acidic (-5)
neutral
Basic (+5)

AtCruC

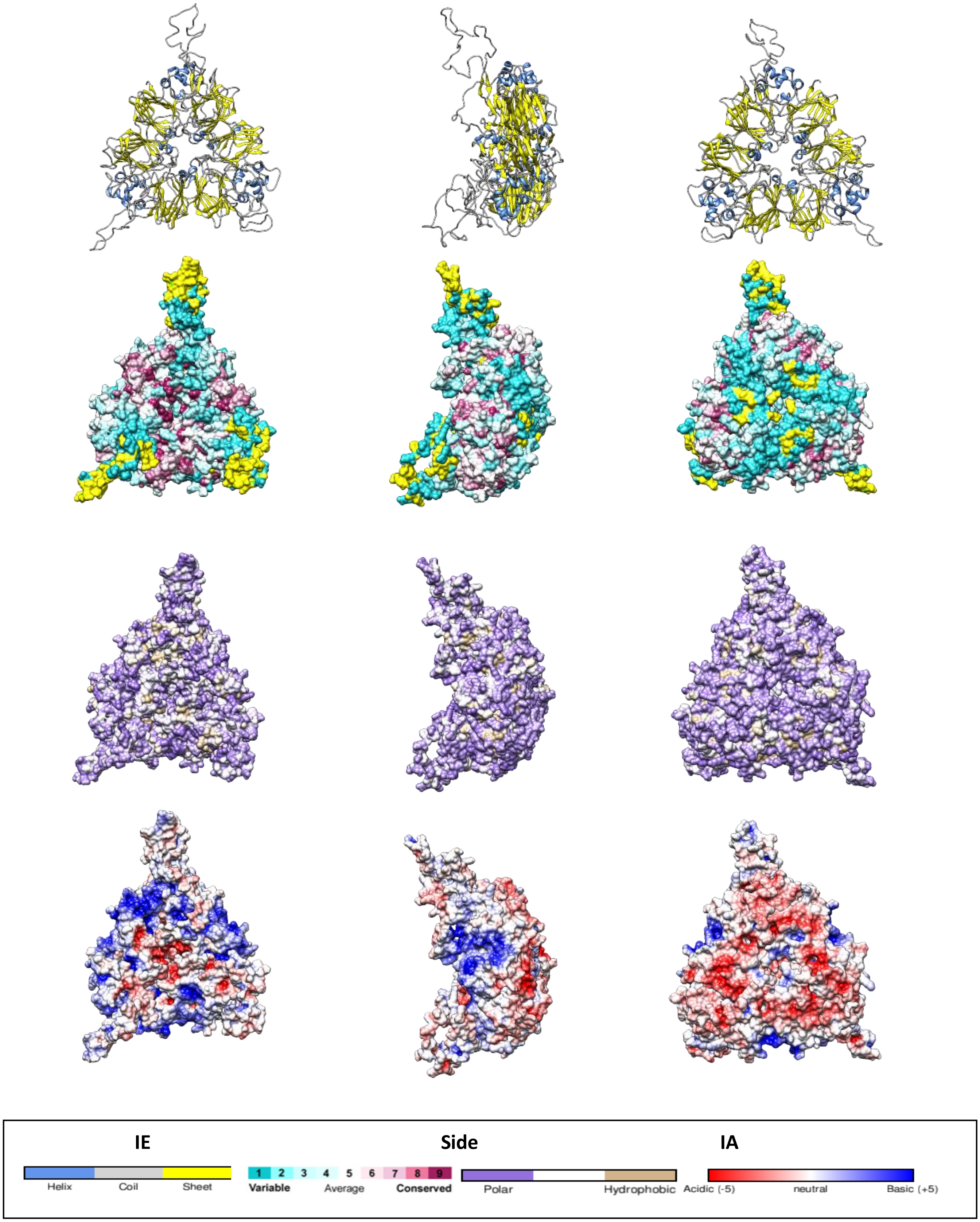
IE
Side
IA
Helix
Coil
Sheet
1 2 3 4 5 6 7 8 9
Variable
Average
Conserved
Polar
Hydrophobic
Acidic (-5)
neutral
Basic (+5)

CsCRA-1-G1

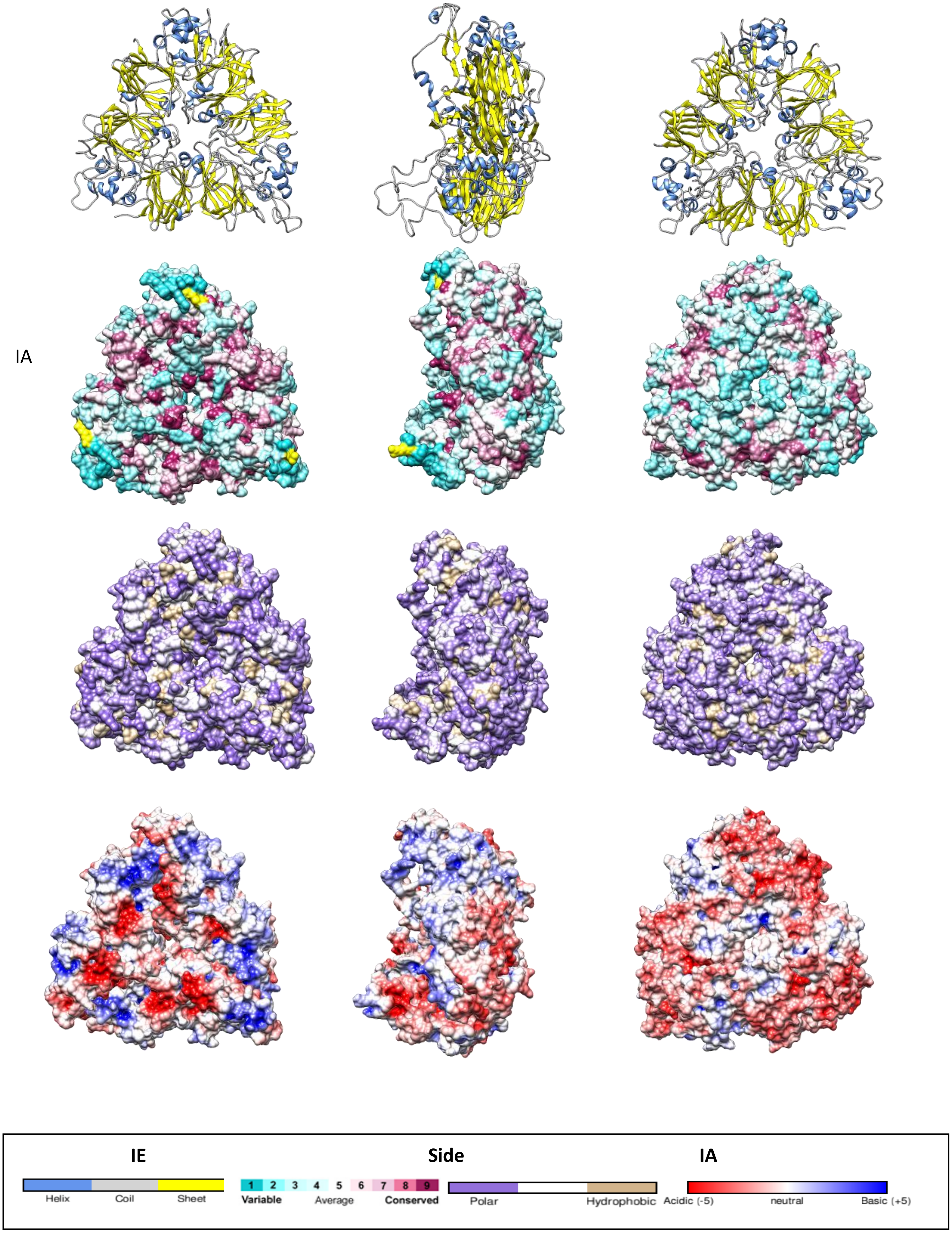
IA
IE
Side
IA
Helix
Coil
Sheet
1 2 3 4 5 6 7 8 9
Variable
Average
Conserved
Polar
Hydrophobic
Acidic (-5)
neutral
Basic (+5)

CsCRB-1-G1

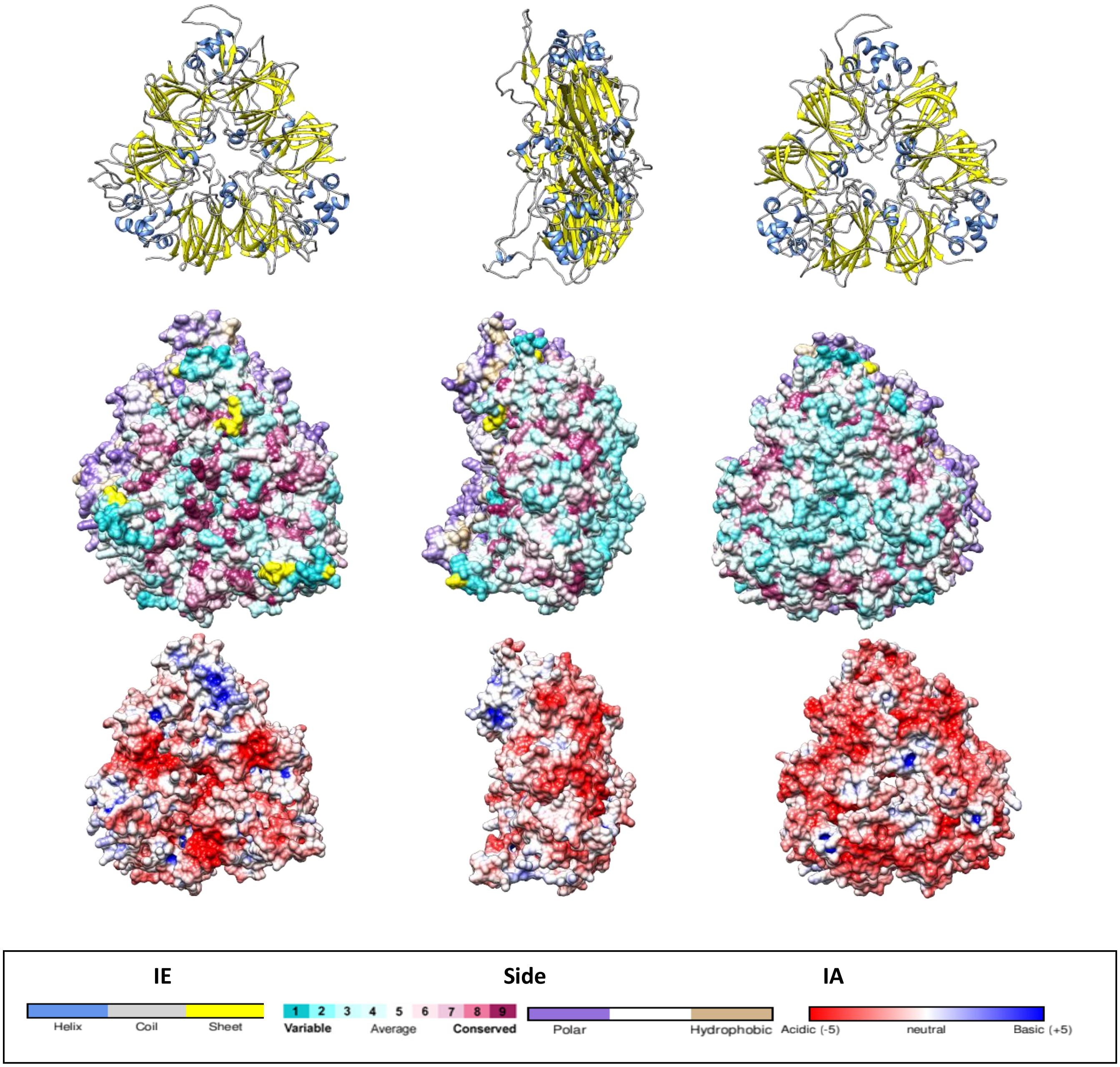

IE
Side
IA
Helix
Coil
Sheet
1
2
3
4
5
6
7
8
9
Variable
Average
Conserved
Polar
Hydrophobic
Acidic (-5)
neutral
Basic (+5)

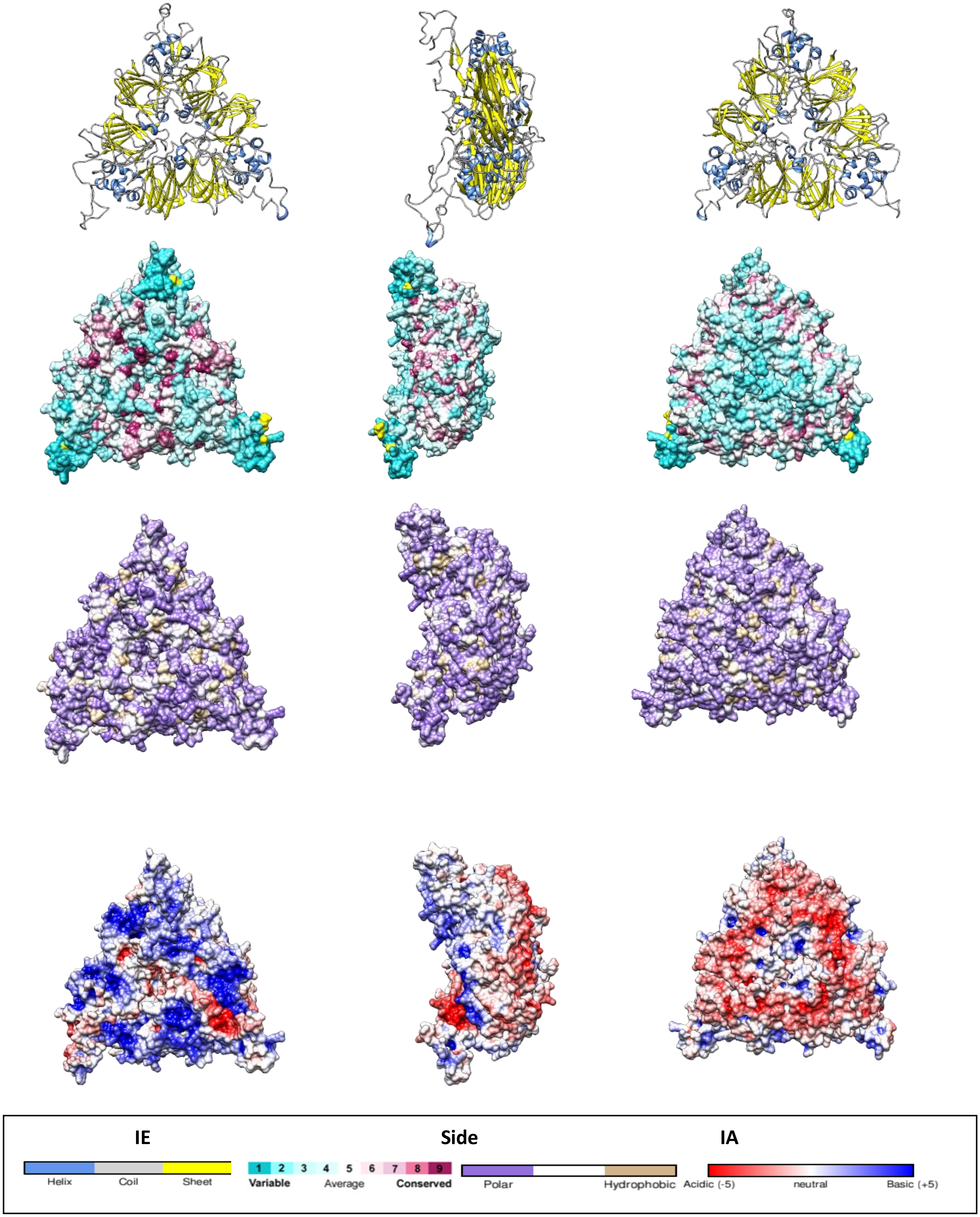

IE
Side
IA
Helix
Coil
Sheet
1 2 3 4 5 6 7 8 9
Variable
Average
Conserved
Polar
Hydrophobic
Acidic (-5)
neutral
Basic (+5)

CsCRD-1-G1

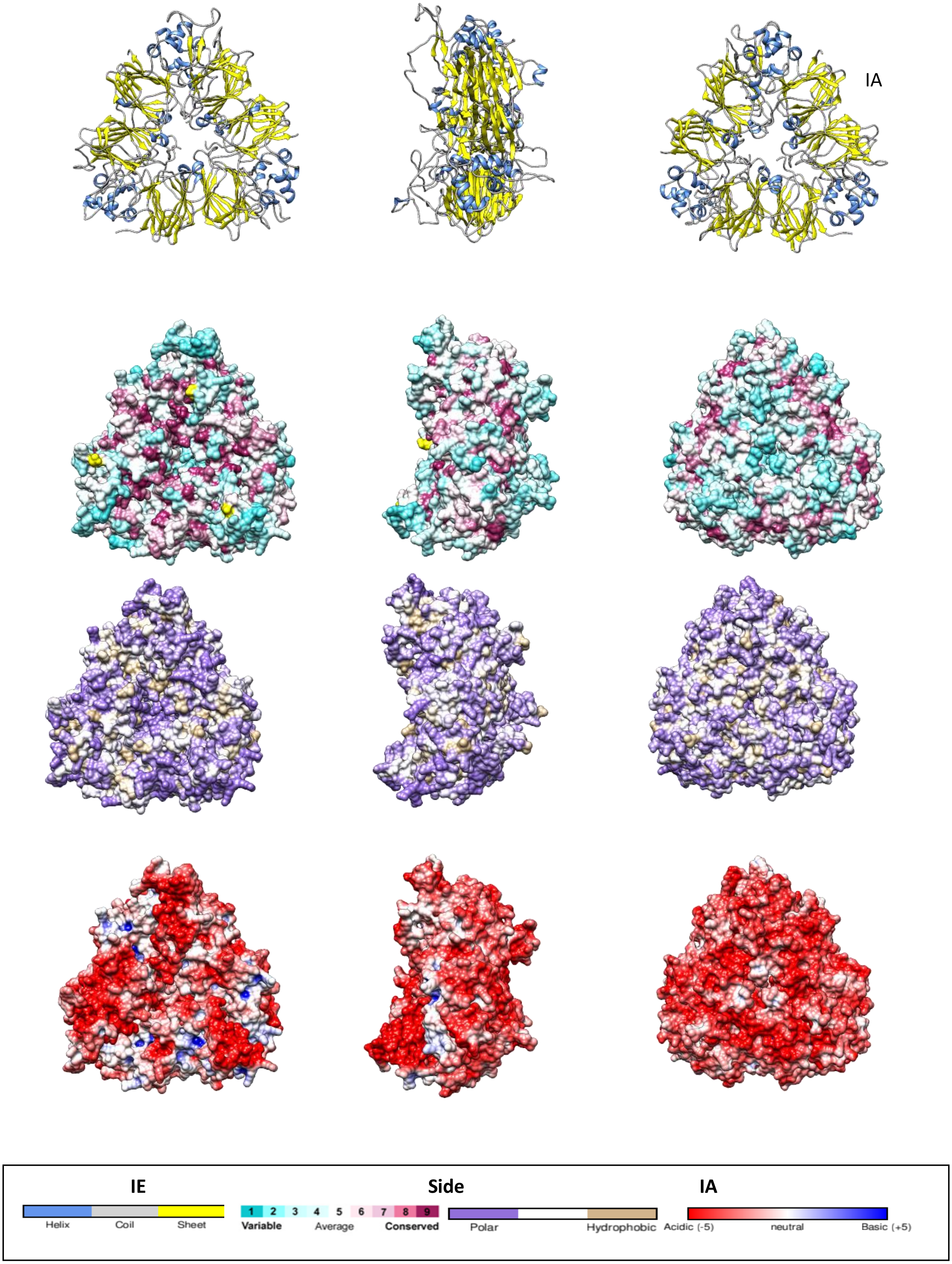
IA
IE
Side
IA
Helix
Coil
Sheet
1 2 3 4 5 6 7 8 9
Variable
Average
Conserved
Polar
Hydrophobic
Acidic (-5)
neutral
Basic (+5)
